# Supplementary material for: Clinical code usage in UK general practice: a cohort study exploring 18 conditions over 14 years
Source: BMJ Open. 2022 Jul 25;12(7):e051456. doi: 10.1136/bmjopen-2021-051456 (PMC9328099; doi:10.1136/bmjopen-2021-051456)
Supplement: Supplementary data [file bmjopen-2021-051456supp001.pdf]

BMJ Publishing Group Limited (BMJ) disclaims all liability and responsibility arising from any reliance placed on this supplemental material which has been supplied by the author(s)

Supplementary data to "Clinical code usage in UK general practice: a cohort study exploring 18 conditions over 14 years", Zghebi et al. 2021.

Supplementary data

Zghebi SS, *et al. BMJ Open* 2022; 12:e051456. doi: 10.1136/bmjopen-2021-051456

Supplementary data to "Clinical code usage in UK general practice: a cohort study exploring 18 conditions over 14 years", Zghebi et al. 2021.

Table S1 Diabetes Read code list

	Code	Coding system	Description
1.	66A3.00	Read	Diabetic on diet only
2.	66A4.00	Read	Diabetic on oral treatment
3.	66A5.00	Read	Diabetic on insulin
4.	66AI.00	Read	Diabetic - good control
5.	66AJ.00	Read	Diabetic - poor control
6.	66AJ100	Read	Brittle diabetes
7.	66AJ.11	Read	Unstable diabetes
8.	66AJz00	Read	Diabetic - poor control NOS
9.	66AK.00	Read	Diabetic - cooperative patient
10.	66AL.00	Read	Diabetic-uncooperative patient
11.	66AV.00	Read	Diabetic on insulin and oral treatment
12.	C10..00	Read	Diabetes mellitus
13.	C100.00	Read	Diabetes mellitus with no mention of complication
14.	C100000	Read	Diabetes mellitus; juvenile type; no mention of complication
15.	C100011	Read	Insulin dependent diabetes mellitus
16.	C100100	Read	Diabetes mellitus; adult onset; no mention of complication
17.	C100111	Read	Maturity onset diabetes
18.	C100112	Read	Non-insulin dependent diabetes mellitus
19.	C100z00	Read	Diabetes mellitus NOS with no mention of complication
20.	C101.00	Read	Diabetes mellitus with ketoacidosis
21.	C101000	Read	Diabetes mellitus; juvenile type; with ketoacidosis
22.	C101100	Read	Diabetes mellitus; adult onset; with ketoacidosis
23.	C101y00	Read	Other specified diabetes mellitus with ketoacidosis
24.	C101z00	Read	Diabetes mellitus NOS with ketoacidosis
25.	C102.00	Read	Diabetes mellitus with hyperosmolar coma
26.	C102000	Read	Diabetes mellitus; juvenile type; with hyperosmolar coma
27.	C102100	Read	Diabetes mellitus; adult onset; with hyperosmolar coma
28.	C102z00	Read	Diabetes mellitus NOS with hyperosmolar coma
29.	C103.00	Read	Diabetes mellitus with ketoacidotic coma
30.	C103000	Read	Diabetes mellitus; juvenile type; with ketoacidotic coma
31.	C103100	Read	Diabetes mellitus; adult onset; with ketoacidotic coma
32.	C103y00	Read	Other specified diabetes mellitus with coma
33.	C103z00	Read	Diabetes mellitus NOS with ketoacidotic coma
34.	C104.00	Read	Diabetes mellitus with renal manifestation
35.	C104000	Read	Diabetes mellitus; juvenile type; with renal manifestation
36.	C104100	Read	Diabetes mellitus; adult onset; with renal manifestation
37.	C104y00	Read	Other specified diabetes mellitus with renal complications
38.	C104z00	Read	Diabetes mellitis with nephropathy NOS
39.	C105.00	Read	Diabetes mellitus with ophthalmic manifestation
40.	C105000	Read	Diabetes mellitus; juvenile type; + ophthalmic manifestation
41.	C105100	Read	Diabetes mellitus; adult onset; + ophthalmic manifestation
42.	C105y00	Read	Other specified diabetes mellitus with ophthalmic complicatn
43.	C105z00	Read	Diabetes mellitus NOS with ophthalmic manifestation
44.	C106.00	Read	Diabetes mellitus with neurological manifestation
45.	C106000	Read	Diabetes mellitus; juvenile; + neurological manifestation
46.	C106100	Read	Diabetes mellitus; adult onset; + neurological manifestation
47.	C106.12	Read	Diabetes mellitus with neuropathy

Supplementary data to "Clinical code usage in UK general practice: a cohort study exploring 18 conditions over 14 years", Zghebi et al. 2021.

48.	C106.13	Read	Diabetes mellitus with polyneuropathy
49.	C106y00	Read	Other specified diabetes mellitus with neurological comps
50.	C106z00	Read	Diabetes mellitus NOS with neurological manifestation
51.	C107.00	Read	Diabetes mellitus with peripheral circulatory disorder
52.	C107000	Read	Diabetes mellitus; juvenile +peripheral circulatory disorder
53.	C107100	Read	Diabetes mellitus; adult; + peripheral circulatory disorder
54.	C107.11	Read	Diabetes mellitus with gangrene
55.	C107.12	Read	Diabetes with gangrene
56.	C107200	Read	Diabetes mellitus; adult with gangrene
57.	C107300	Read	IDDM with peripheral circulatory disorder
58.	C107400	Read	NIDDM with peripheral circulatory disorder
59.	C107z00	Read	Diabetes mellitus NOS with peripheral circulatory disorder
60.	C108.00	Read	Insulin dependent diabetes mellitus
61.	C108000	Read	Insulin-dependent diabetes mellitus with renal complications
62.	C108011	Read	Type I diabetes mellitus with renal complications
63.	C108012	Read	Type 1 diabetes mellitus with renal complications
64.	C108100	Read	Insulin-dependent diabetes mellitus with ophthalmic comps
65.	C108.11	Read	IDDM-Insulin dependent diabetes mellitus
66.	C108.12	Read	Type 1 diabetes mellitus
67.	C108.13	Read	Type I diabetes mellitus
68.	C108200	Read	Insulin-dependent diabetes mellitus with neurological comps
69.	C108211	Read	Type I diabetes mellitus with neurological complications
70.	C108212	Read	Type 1 diabetes mellitus with neurological complications
71.	C108300	Read	Insulin dependent diabetes mellitus with multiple complicatn
72.	C108400	Read	Unstable insulin dependent diabetes mellitus
73.	C108411	Read	Unstable type I diabetes mellitus
74.	C108500	Read	Insulin dependent diabetes mellitus with ulcer
75.	C108511	Read	Type I diabetes mellitus with ulcer
76.	C108600	Read	Insulin dependent diabetes mellitus with gangrene
77.	C108700	Read	Insulin dependent diabetes mellitus with retinopathy
78.	C108711	Read	Type I diabetes mellitus with retinopathy
79.	C108712	Read	Type 1 diabetes mellitus with retinopathy
80.	C108800	Read	Insulin dependent diabetes mellitus - poor control
81.	C108811	Read	Type I diabetes mellitus - poor control
82.	C108812	Read	Type 1 diabetes mellitus - poor control
83.	C108900	Read	Insulin dependent diabetes maturity onset
84.	C108911	Read	Type I diabetes mellitus maturity onset
85.	C108A00	Read	Insulin-dependent diabetes without complication
86.	C108B00	Read	Insulin dependent diabetes mellitus with mononeuropathy
87.	C108B11	Read	Type I diabetes mellitus with mononeuropathy
88.	C108C00	Read	Insulin dependent diabetes mellitus with polyneuropathy
89.	C108D00	Read	Insulin dependent diabetes mellitus with nephropathy
90.	C108D11	Read	Type I diabetes mellitus with nephropathy
91.	C108E00	Read	Insulin dependent diabetes mellitus with hypoglycaemic coma
92.	C108E11	Read	Type I diabetes mellitus with hypoglycaemic coma
93.	C108E12	Read	Type 1 diabetes mellitus with hypoglycaemic coma
94.	C108F00	Read	Insulin dependent diabetes mellitus with diabetic cataract
95.	C108F11	Read	Type I diabetes mellitus with diabetic cataract
96.	C108G00	Read	Insulin dependent diab mell with peripheral angiopathy
97.	C108H00	Read	Insulin dependent diabetes mellitus with arthropathy

Supplementary data to "Clinical code usage in UK general practice: a cohort study exploring 18 conditions over 14 years", Zghebi et al. 2021.

98.	C108H11	Read	Type I diabetes mellitus with arthropathy
99.	C108J00	Read	Insulin dependent diab mell with neuropathic arthropathy
100.	C108J12	Read	Type 1 diabetes mellitus with neuropathic arthropathy
101.	C108y00	Read	Other specified diabetes mellitus with multiple comps
102.	C108z00	Read	Unspecified diabetes mellitus with multiple complications
103.	C109.00	Read	Non-insulin-dependent diabetes mellitus
104.	C109000	Read	Non-insulin-dependent diabetes mellitus with renal comps
105.	C109011	Read	Type II diabetes mellitus with renal complications
106.	C109012	Read	Type 2 diabetes mellitus with renal complications
107.	C109100	Read	Non-insulin-dependent diabetes mellitus with ophthalm comps
108.	C109.11	Read	NIDDM - Non-insulin dependent diabetes mellitus
109.	C109111	Read	Type II diabetes mellitus with ophthalmic complications
110.	C109112	Read	Type 2 diabetes mellitus with ophthalmic complications
111.	C109.12	Read	Type 2 diabetes mellitus
112.	C109.13	Read	Type II diabetes mellitus
113.	C109200	Read	Non-insulin-dependent diabetes mellitus with neuro comps
114.	C109211	Read	Type II diabetes mellitus with neurological complications
115.	C109212	Read	Type 2 diabetes mellitus with neurological complications
116.	C109300	Read	Non-insulin-dependent diabetes mellitus with multiple comps
117.	C109400	Read	Non-insulin dependent diabetes mellitus with ulcer
118.	C109411	Read	Type II diabetes mellitus with ulcer
119.	C109412	Read	Type 2 diabetes mellitus with ulcer
120.	C109500	Read	Non-insulin dependent diabetes mellitus with gangrene
121.	C109511	Read	Type II diabetes mellitus with gangrene
122.	C109600	Read	Non-insulin-dependent diabetes mellitus with retinopathy
123.	C109611	Read	Type II diabetes mellitus with retinopathy
124.	C109612	Read	Type 2 diabetes mellitus with retinopathy
125.	C109700	Read	Non-insulin dependant diabetes mellitus - poor control
126.	C109711	Read	Type II diabetes mellitus - poor control
127.	C109712	Read	Type 2 diabetes mellitus - poor control
128.	C109900	Read	Non-insulin-dependent diabetes mellitus without complication
129.	C109A00	Read	Non-insulin dependent diabetes mellitus with mononeuropathy
130.	C109A11	Read	Type II diabetes mellitus with mononeuropathy
131.	C109B00	Read	Non-insulin dependent diabetes mellitus with polyneuropathy
132.	C109B11	Read	Type II diabetes mellitus with polyneuropathy
133.	C109C00	Read	Non-insulin dependent diabetes mellitus with nephropathy
134.	C109C11	Read	Type II diabetes mellitus with nephropathy
135.	C109C12	Read	Type 2 diabetes mellitus with nephropathy
136.	C109D00	Read	Non-insulin dependent diabetes mellitus with hypoglyca coma
137.	C109D11	Read	Type II diabetes mellitus with hypoglycaemic coma
138.	C109D12	Read	Type 2 diabetes mellitus with hypoglycaemic coma
139.	C109E00	Read	Non-insulin depend diabetes mellitus with diabetic cataract
140.	C109E11	Read	Type II diabetes mellitus with diabetic cataract
141.	C109E12	Read	Type 2 diabetes mellitus with diabetic cataract
142.	C109F00	Read	Non-insulin-dependent d m with peripheral angiopath
143.	C109F11	Read	Type II diabetes mellitus with peripheral angiopathy
144.	C109F12	Read	Type 2 diabetes mellitus with peripheral angiopathy
145.	C109G00	Read	Non-insulin dependent diabetes mellitus with arthropathy
146.	C109G11	Read	Type II diabetes mellitus with arthropathy
147.	C109G12	Read	Type 2 diabetes mellitus with arthropathy

Supplementary data to "Clinical code usage in UK general practice: a cohort study exploring 18 conditions over 14 years", Zghebi et al. 2021.

148.	C109H00	Read	Non-insulin dependent d m with neuropathic arthropathy
149.	C109H11	Read	Type II diabetes mellitus with neuropathic arthropathy
150.	C109H12	Read	Type 2 diabetes mellitus with neuropathic arthropathy
151.	C109J00	Read	Insulin treated Type 2 diabetes mellitus
152.	C109J11	Read	Insulin treated non-insulin dependent diabetes mellitus
153.	C109J12	Read	Insulin treated Type II diabetes mellitus
154.	C109K00	Read	Hyperosmolar non-ketotic state in type 2 diabetes mellitus
155.	C10C.00	Read	Diabetes mellitus autosomal dominant
156.	C10C.11	Read	Maturity onset diabetes in youth
157.	C10D.00	Read	Diabetes mellitus autosomal dominant type 2
158.	C10D.11	Read	Maturity onset diabetes in youth type 2
159.	C10E.00	Read	Type 1 diabetes mellitus
160.	C10E000	Read	Type 1 diabetes mellitus with renal complications
161.	C10E100	Read	Type 1 diabetes mellitus with ophthalmic complications
162.	C10E.11	Read	Type I diabetes mellitus
163.	C10E.12	Read	Insulin dependent diabetes mellitus
164.	C10E200	Read	Type 1 diabetes mellitus with neurological complications
165.	C10E300	Read	Type 1 diabetes mellitus with multiple complications
166.	C10E312	Read	Insulin dependent diabetes mellitus with multiple complicat
167.	C10E400	Read	Unstable type 1 diabetes mellitus
168.	C10E411	Read	Unstable type I diabetes mellitus
169.	C10E412	Read	Unstable insulin dependent diabetes mellitus
170.	C10E500	Read	Type 1 diabetes mellitus with ulcer
171.	C10E600	Read	Type 1 diabetes mellitus with gangrene
172.	C10E700	Read	Type 1 diabetes mellitus with retinopathy
173.	C10E800	Read	Type 1 diabetes mellitus - poor control
174.	C10E812	Read	Insulin dependent diabetes mellitus - poor control
175.	C10E900	Read	Type 1 diabetes mellitus maturity onset
176.	C10EA00	Read	Type 1 diabetes mellitus without complication
177.	C10EA11	Read	Type I diabetes mellitus without complication
178.	C10EB00	Read	Type 1 diabetes mellitus with mononeuropathy
179.	C10EC00	Read	Type 1 diabetes mellitus with polyneuropathy
180.	C10ED00	Read	Type 1 diabetes mellitus with nephropathy
181.	C10EE00	Read	Type 1 diabetes mellitus with hypoglycaemic coma
182.	C10EF00	Read	Type 1 diabetes mellitus with diabetic cataract
183.	C10EG00	Read	Type 1 diabetes mellitus with peripheral angiopathy
184.	C10EH00	Read	Type 1 diabetes mellitus with arthropathy
185.	C10EJ00	Read	Type 1 diabetes mellitus with neuropathic arthropathy
186.	C10EK00	Read	Type 1 diabetes mellitus with persistent proteinuria
187.	C10EL00	Read	Type 1 diabetes mellitus with persistent microalbuminuria
188.	C10EM00	Read	Type 1 diabetes mellitus with ketoacidosis
189.	C10EM11	Read	Type I diabetes mellitus with ketoacidosis
190.	C10EN00	Read	Type 1 diabetes mellitus with ketoacidotic coma
191.	C10EN11	Read	Type I diabetes mellitus with ketoacidotic coma
192.	C10EP00	Read	Type 1 diabetes mellitus with exudative maculopathy
193.	C10EQ00	Read	Type 1 diabetes mellitus with gastroparesis
194.	C10F.00	Read	Type 2 diabetes mellitus
195.	C10F000	Read	Type 2 diabetes mellitus with renal complications
196.	C10F011	Read	Type II diabetes mellitus with renal complications
197.	C10F100	Read	Type 2 diabetes mellitus with ophthalmic complications

Supplementary data to "Clinical code usage in UK general practice: a cohort study exploring 18 conditions over 14 years", Zghebi et al. 2021.

198.	C10F.11	Read	Type II diabetes mellitus
199.	C10F200	Read	Type 2 diabetes mellitus with neurological complications
200.	C10F300	Read	Type 2 diabetes mellitus with multiple complications
201.	C10F311	Read	Type II diabetes mellitus with multiple complications
202.	C10F400	Read	Type 2 diabetes mellitus with ulcer
203.	C10F500	Read	Type 2 diabetes mellitus with gangrene
204.	C10F511	Read	Type II diabetes mellitus with gangrene
205.	C10F600	Read	Type 2 diabetes mellitus with retinopathy
206.	C10F611	Read	Type II diabetes mellitus with retinopathy
207.	C10F700	Read	Type 2 diabetes mellitus - poor control
208.	C10F711	Read	Type II diabetes mellitus - poor control
209.	C10F900	Read	Type 2 diabetes mellitus without complication
210.	C10F911	Read	Type II diabetes mellitus without complication
211.	C10FA00	Read	Type 2 diabetes mellitus with mononeuropathy
212.	C10FB00	Read	Type 2 diabetes mellitus with polyneuropathy
213.	C10FB11	Read	Type II diabetes mellitus with polyneuropathy
214.	C10FC00	Read	Type 2 diabetes mellitus with nephropathy
215.	C10FC11	Read	Type II diabetes mellitus with nephropathy
216.	C10FD00	Read	Type 2 diabetes mellitus with hypoglycaemic coma
217.	C10FE00	Read	Type 2 diabetes mellitus with diabetic cataract
218.	C10FF00	Read	Type 2 diabetes mellitus with peripheral angiopathy
219.	C10FG00	Read	Type 2 diabetes mellitus with arthropathy
220.	C10FH00	Read	Type 2 diabetes mellitus with neuropathic arthropathy
221.	C10FJ00	Read	Insulin treated Type 2 diabetes mellitus
222.	C10FJ11	Read	Insulin treated Type II diabetes mellitus
223.	C10FK00	Read	Hyperosmolar non-ketotic state in type 2 diabetes mellitus
224.	C10FL00	Read	Type 2 diabetes mellitus with persistent proteinuria
225.	C10FL11	Read	Type II diabetes mellitus with persistent proteinuria
226.	C10FM00	Read	Type 2 diabetes mellitus with persistent microalbuminuria
227.	C10FN00	Read	Type 2 diabetes mellitus with ketoacidosis
228.	C10FP00	Read	Type 2 diabetes mellitus with ketoacidotic coma
229.	C10FQ00	Read	Type 2 diabetes mellitus with exudative maculopathy
230.	C10FR00	Read	Type 2 diabetes mellitus with gastroparesis
231.	C10G.00	Read	Secondary pancreatic diabetes mellitus
232.	C10G000	Read	Secondary pancreatic diabetes mellitus without complication
233.	C10y.00	Read	Diabetes mellitus with other specified manifestation
234.	C10y100	Read	Diabetes mellitus; adult; + other specified manifestation
235.	C10yy00	Read	Other specified diabetes mellitus with other spec comps
236.	C10yz00	Read	Diabetes mellitus NOS with other specified manifestation
237.	C10z.00	Read	Diabetes mellitus with unspecified complication
238.	C10z000	Read	Diabetes mellitus; juvenile type; + unspecified complication
239.	C10z100	Read	Diabetes mellitus; adult onset; + unspecified complication
240.	C10zy00	Read	Other specified diabetes mellitus with unspecified comps
241.	C10zz00	Read	Diabetes mellitus NOS with unspecified complication
242.	Cyu2.00	Read	[X]Diabetes mellitus
243.	Cyu2000	Read	[X]Other specified diabetes mellitus
244.	Cyu2300	Read	[X]Unspecified diabetes mellitus with renal complications
245.	L180500	Read	Pre-existing diabetes mellitus; insulin-dependent
246.	L180600	Read	Pre-existing diabetes mellitus; non-insulin-dependent
247.	L180X00	Read	Pre-existing diabetes mellitus; unspecified

Zghebi SS, *et al. BMJ Open* 2022; 12:e051456. doi: 10.1136/bmjopen-2021-051456

Supplementary data to "Clinical code usage in UK general practice: a cohort study exploring 18 conditions over 14 years", Zghebi et al. 2021.

Table S2 R code

1, 3, 5, year Jackknife correlations (Figures 2, S3, S4)

```
library(dplyr)
library(tidyr)
library(ggplot2)
library(stringr)

setwd("Q:/code_usage")
rm(list=ls())
conditions <- c("asthma", "atrial_fibrillation", "cancer", "chd", "ckd",
            "copd", "dementia", "depression", "dm", "epilepsy", "hf", "ht","hypothyroidism" ,
            "learning_disability", "osteoarthritis", "osteoporosis", "smi", "stroke")


jack_incidence <- new.env()
jack_total <- new.env()

for(condition in conditions){
   if(file.exists(myfile <- paste0("data/", condition, "_year_cors.rda"))){
      message("Loading ", myfile)
      load(myfile, envir = jack_incidence)
   }
   if(file.exists(myfile <- paste0("data/", condition, "_total_cors.rda"))){
      message("Loading ", myfile)
      load(myfile, envir = jack_total)
   }

}

incid_data <- bind_rows(lapply(ls(jack_incidence), function(condition){
   bind_rows(jack_incidence[[condition]]) %>%
      mutate(condition = str_replace(condition, "_year_cors", ""),
           type = "incident codes")
}))

total_data <- bind_rows(lapply(ls(jack_total), function(condition){
   bind_rows(jack_total[[condition]]) %>%
      mutate(condition = str_replace(condition, "_total_cors", ""),
           type = "all codes")
}))

jack_data <- bind_rows(incid_data, total_data) %>%
   mutate(year1 = as.numeric(year1))


## Year-on-year correlations:
p <- ggplot(jack_data %>% filter(num < 14),
         aes(x = year1, y = cor, colour = type))
p + geom_point() +
   geom_line() +
```

Supplementary data to "Clinical code usage in UK general practice: a cohort study exploring 18 conditions over 14 years", Zghebi et al. 2021.

```
   geom_errorbar(aes(ymin = lower, ymax = upper), width = 0.25) +
   facet_wrap( ~ condition) +
   theme_bw() +
   scale_x_continuous("Year", breaks = c(2000, 2002, 2004, 2006, 2008, 2010, 2012)) +
   scale_y_continuous("1st cannonical correlation", limits = c(0,1)) +
   theme(axis.text.x = element_text(angle = 45, hjust = 1)) +
   #labs(title = "")
ggsave("analysis/1-jack_corr_1yearwindow.pdf")


## Bi-yearly correlations:
p <- ggplot(jack_data %>% filter(num >= 14, num < 24),
          aes(x = year1, y = cor, colour = type))
p + geom_point() +
   geom_line() +
   geom_errorbar(aes(ymin = lower, ymax = upper), width = 0.25) +
   facet_wrap( ~ condition) +
   theme_bw() +
   scale_x_continuous("Year", breaks = c(2000, 2002, 2004, 2006, 2008, 2010, 2012)) +
   scale_y_continuous("1st cannonical correlation",
                 limits = c(0,1)) +
   theme(axis.text.x = element_text(angle = 45, hjust = 1)) +
   #labs(title = "")
ggsave("analysis/2-jack_corr_3yearwindow.pdf")

## 5-yearly correlations:
p <- ggplot(jack_data %>% filter(num >= 14, num < 24),
          aes(x = year1, y = cor, colour = type))
p + geom_point() +
   geom_line() +
   geom_errorbar(aes(ymin = lower, ymax = upper), width = 0.25) +
   facet_wrap( ~ condition) +
   theme_bw() +
   scale_x_continuous("Year", breaks = c(2000, 2002, 2004, 2006, 2008, 2010, 2012)) +
   scale_y_continuous("1st cannonical correlation", limits = c(0,1)) +
   #labs(title = "")
ggsave("analysis/3-jack_corr_5yearwindow.pdf")
```

Canonical_correlations_5-year windows (for Figure S2)

```
library(rEHR)
library(dplyr)
library(tidyr)
library(ggplot2)

rm(list=ls())
conditions <- c("asthma", "atrial_fibrillation", "cancer", "chd", "ckd",
             "copd", "dementia", "depression", "dm", "epilepsy", "hf", "ht","hypothyroidism" ,
             "learning_disability", "osteoarthritis", "osteoporosis", "smi", "stroke")
correlations <- new.env()
```

Supplementary data to "Clinical code usage in UK general practice: a cohort study exploring 18 conditions over 14 years", Zghebi et al. 2021.

```
for(condition in conditions){
   load(paste0("data/", condition, "_year_cors.rda"), envir = correlations)
   my_corr <- get(paste0(condition, "_year_cors"),  envir = correlations)
   my_corr <-  lapply(my_corr, function(x) {
        x$condition <- condition
        x
   })
   assign(paste0(condition, "_year_cors"), my_corr, envir = correlations)
}
rm(my_corr)

all_corrs <- bind_rows(lapply(correlations, function(corr){
   data_frame(year = 2005:2013,
           cor = unlist(lapply(corr, function(x) x$cor)),
           condition = corr[[1]]$condition)
}))


p <- ggplot(all_corrs, aes(x = year, y = cor))
p + geom_line() +
   facet_wrap( ~ condition) +
#    geom_vline(x = 2004, linetype  = "longdash", colour = "red", width = 4) +
   theme_bw() +
   labs(title  = "Correlations of clinical code usage (Five year windows)") +
   scale_y_continuous("Spearman's canonical correlation coefficient") +
   scale_x_continuous(breaks = c(2004, 2006, 2008, 2010, 2012)) +
   theme(axis.text.x = element_text(angle = 45, hjust = 1))

ggsave("analysis/canonical_correlations_5yearwindows.pdf")
```

Three diversity measures (Figures 3, 4, 5)

```
library(rEHR)
library(dplyr)
library(tidyr)
library(parallel)
library(ggplot2)
library(bootstrap)

setwd("Q:/code_usage")
#' calculates the Shannon index of diversity/entropy
shannon <- function(x)
{
   x <- drop(as.matrix(x))
   if (length(dim(x)) > 1) {
      total <- apply(x, 1, sum)
      x <- sweep(x, 1, total, "/")
   }
   else {
      x <- x/sum(x)
   }
```

Supplementary data to "Clinical code usage in UK general practice: a cohort study exploring 18 conditions over 14 years", Zghebi et al. 2021.

```
  x <- -x * log(x)

  if (length(dim(x)) > 1)
    H <- apply(x, sum, na.rm = TRUE)
  else H <- sum(x, na.rm = TRUE)
  H
}

evenness_fn <- function(x){
  H <- shannon(x)
  R <- length(x)
  H / log(R)
}


jack_stats <- function(x, thetahat){
  n <- length(x)
  bias <- (n - 1) * (mean(x) - thetahat)
  se <- sqrt(((n - 1)/n) * sum((x - mean(x))^2))
  list(param = thetahat, bias = bias, se = se)
}

conditions <- c("asthma", "atrial_fibrillation", "blood_pressure", "cancer", "chd", "ckd",
        "copd", "dementia", "depression", "dm", "epilepsy", "hf", "ht","hypothyroidism" ,
        "learning_disability", "osteoarthritis", "osteoporosis", "smi", "stroke")
frequencies <- new.env()

for(condition in conditions){
  load(paste0("data/", condition, "_frequencies.rda"), envir = frequencies)
  incid <- get(paste0(condition, "_incidence_freqs"),  envir = frequencies)
  incid$type <- "incident codes"
  assign(paste0(condition, "_incidence_freqs"), incid, envir = frequencies)

  total <- get(paste0(condition, "_total_freqs"),  envir = frequencies)
  total$type <- "all codes"
  assign(paste0(condition, "_total_freqs"), total, envir = frequencies)

}

all_freqs <- bind_rows(lapply(frequencies, function(x) x))

years <- 2000:2013
jacks <- bind_rows(lapply(frequencies, function(condition){
  bind_rows(mclapply(years, function(this_year){
    dat <- condition %>%
      filter(year == this_year)
    H <- shannon(dat$freq)
    H_jack <- jackknife(dat$freq, theta = shannon)
    richness <- nrow(dat)
    evenness <- evenness_fn(dat$freq)
    evenness_jack <- jackknife(dat$freq, theta = evenness_fn)
    data_frame(year = rep(this_year, 3),
         condition = rep(dat$condition[1], 3),
```

Supplementary data to "Clinical code usage in UK general practice: a cohort study exploring 18 conditions over 14 years", Zghebi et al. 2021.

```
                type = rep(dat$type[1], 3),
                index = c("H", "richness", "evenness"),
                value = c(H, richness, evenness),
                upper = c(H + 1.96 * H_jack$jack.se,
                        NA,
                        evenness + 1.96 * evenness_jack$jack.se),
                lower = c(H - 1.96 * H_jack$jack.se,
                        NA,
                        evenness - 1.96 * evenness_jack$jack.se),
                bias = c(H_jack$jack.bias, NA, evenness_jack$jack.bias))

    }, mc.cores = 1))
}))

p <- ggplot(jacks %>% filter(index == "H", condition != "blood_pressure"),
         aes(x = year, y = value, colour = type))
p + geom_line() +
   facet_wrap( ~ condition) +
   geom_errorbar(aes(ymin = lower, ymax = upper), width = 0.25) +
   #geom_vline(x = 2004, linetype  = "longdash", colour = "red", width = 4) +
   theme_bw() +
   labs(title  = "Entropy of clinical code usage") +
   scale_y_continuous("H, Entropy (diversity) of code useage")
ggsave("analysis/4-entropy.pdf")
ggsave("analysis/4-entropy.png")

p <- ggplot(jacks %>% filter(index == "richness", condition != "blood_pressure"),
         aes(x = year, y = value, colour = type))
p + geom_line() +
   facet_wrap( ~ condition) +
   geom_errorbar(aes(ymin = lower, ymax = upper), width = 0.25) +
   #geom_vline(x = 2004, linetype  = "longdash", colour = "red", width = 4) +
   theme_bw() +
   labs(title  = "Richness of clinical code usage") +
   scale_y_continuous("Total number of codes used")
ggsave("analysis/5-richness.pdf")
ggsave("analysis/5-richness.png")


p <- ggplot(jacks %>% filter(index == "evenness", condition != "blood_pressure"),
         aes(x = year, y = value, colour = type))
p + geom_line() +
   facet_wrap( ~ condition) +
   geom_errorbar(aes(ymin = lower, ymax = upper), width = 0.25) +
   #geom_vline(x = 2004, linetype  = "longdash", colour = "red", width = 4) +
   theme_bw() +
   labs(title  = "Evenness of clinical code usage") +
   scale_y_continuous("J, Evenness of code useage")
ggsave("analysis/6-evenness.pdf")
ggsave("analysis/6-evenness.png")

save(jacks, file = "data/jackknife_entropy.rda")
```

Supplementary data to "Clinical code usage in UK general practice: a cohort study exploring 18 conditions over 14 years", Zghebi et al. 2021.

Figure S1 Canonical correlation using 1-year window of clinical code usage for 18 mental and physical conditions

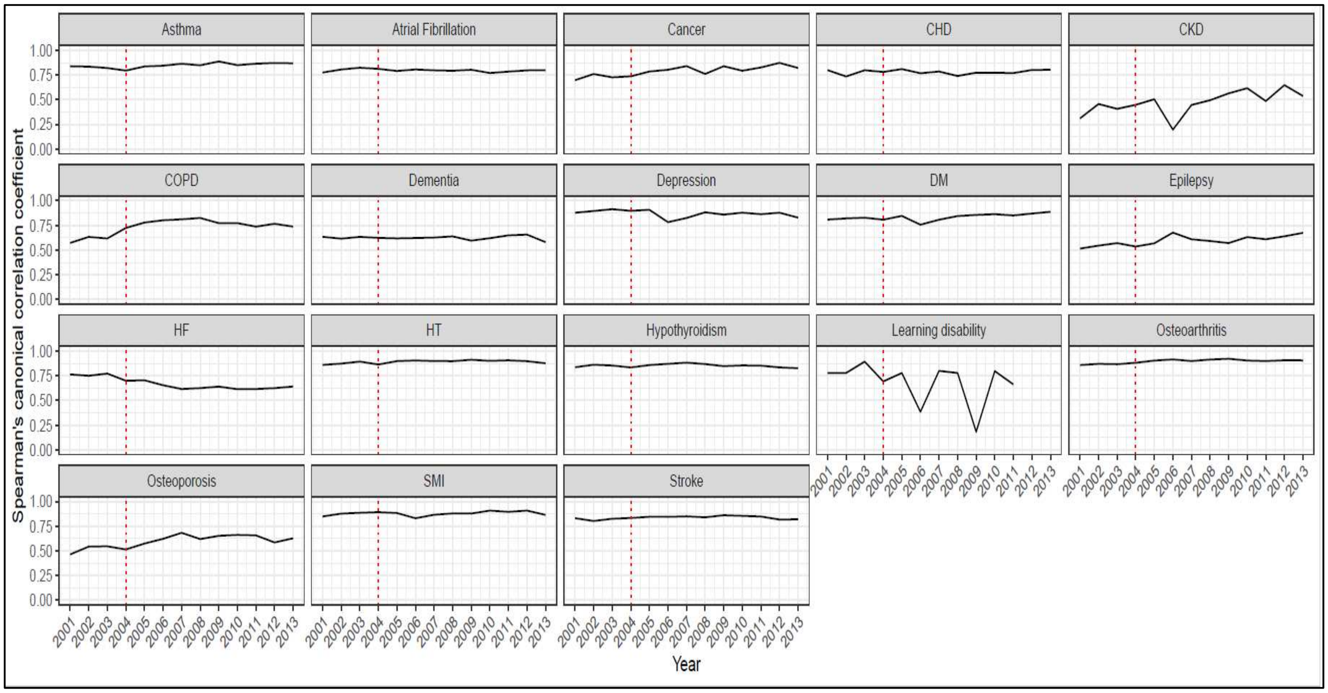

CHD, coronary heart disease; CKD, chronic kidney disease; COPD, chronic obstructive pulmonary disease; DM, diabetes mellitus; HF, heart failure; HT, hypertension; SMI, severe mental illness. The red line represents the launching year of the QOF in 2004.

Zghebi SS, *et al. BMJ Open* 2022; 12:e051456. doi: 10.1136/bmjopen-2021-051456

Supplementary data to "Clinical code usage in UK general practice: a cohort study exploring 18 conditions over 14 years", Zghebi et al. 2021.

Figure S2 Canonical correlation using 5-year window of clinical code usage for 18 mental and physical conditions

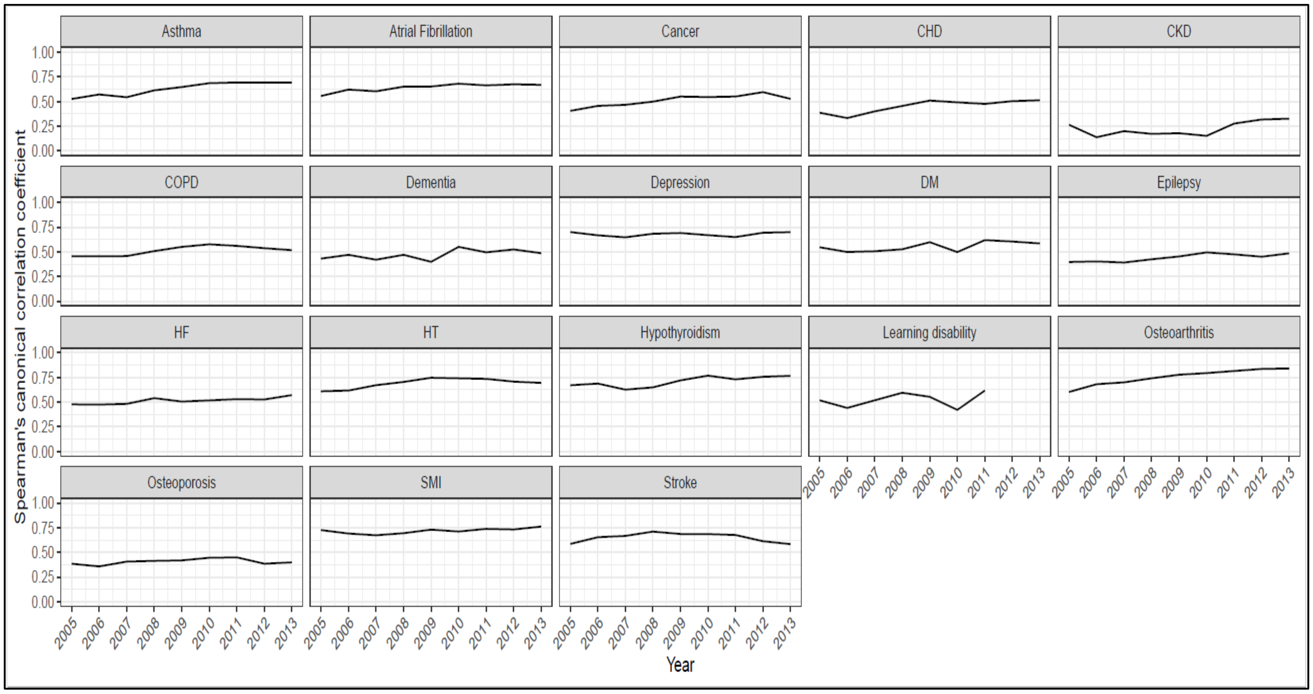

CHD, coronary heart disease; CKD, chronic kidney disease; COPD, chronic obstructive pulmonary disease; DM, diabetes mellitus; HF, heart failure; HT, hypertension; SMI, severe mental illness. The red line represents the launching year of the QOF in 2004.

Supplementary data to "Clinical code usage in UK general practice: a cohort study exploring 18 conditions over 14 years", Zghebi et al. 2021.

Figure S3 Bias-corrected canonical correlations (95%CI) using 1-year window for incident clinical code usage for 18 mental and physical conditions

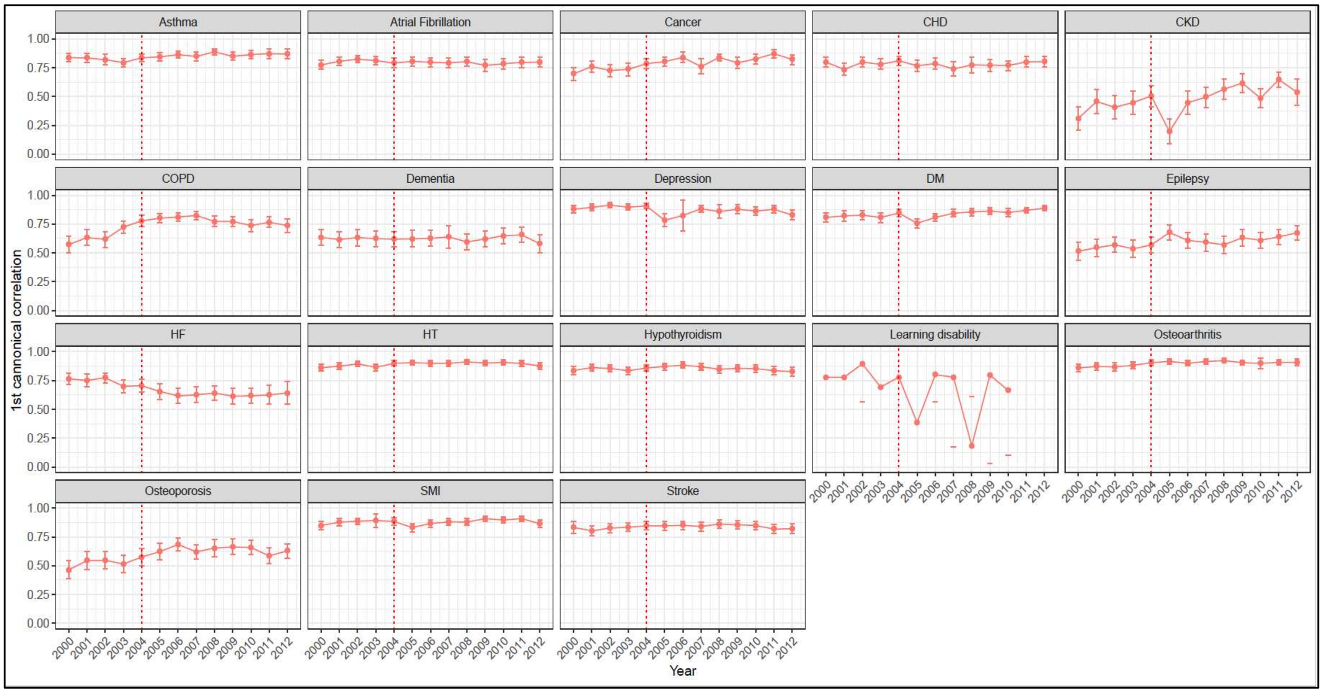

CHD, coronary heart disease; CKD, chronic kidney disease; COPD, chronic obstructive pulmonary disease; DM, diabetes mellitus; HF, heart failure; HT, hypertension; SMI, severe mental illness.

Incident code: a clinical code indicating new (incident) cases. The red line represents the launching year of the QOF in 2004.

Supplementary data to "Clinical code usage in UK general practice: a cohort study exploring 18 conditions over 14 years", Zghebi et al. 2021.

Figure S4 Bias-corrected canonical correlations using 5-year window for incident clinical code usage for 18 mental and physical conditions

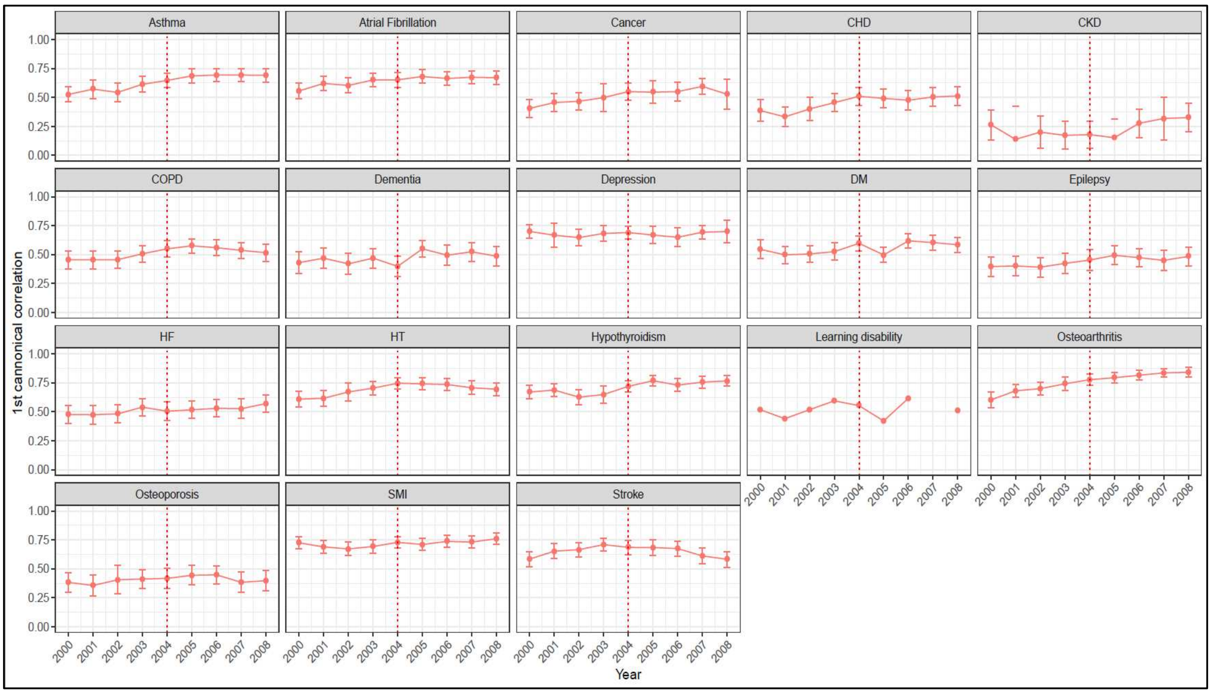

CHD, coronary heart disease; CKD, chronic kidney disease; COPD, chronic obstructive pulmonary disease; DM, diabetes mellitus; HF, heart failure; HT, hypertension; SMI, severe mental illness.

Incident code: a clinical code indicating new (incident) cases. The red line represents the launching year of the QOF in 2004.

Zghebi SS, *et al. BMJ Open* 2022; 12:e051456. doi: 10.1136/bmjopen-2021-051456
