# Supplementary material for: Clinical code usage in UK general practice: a cohort study exploring 18 conditions over 14 years
Source: BMJ Open. 2022 Jul 25;12(7):e051456. doi: 10.1136/bmjopen-2021-051456 (PMC9328099; doi:10.1136/bmjopen-2021-051456)
Supplement: Author's manuscript [file bmjopen-2021-051456.draft_revisions.pdf]

BMJ Open

BMJ Open is committed to open peer review. As part of this commitment we make the peer review history of every article we publish publicly available.

When an article is published we post the peer reviewers' comments and the authors' responses online. We also post the versions of the paper that were used during peer review. These are the versions that the peer review comments apply to.

The versions of the paper that follow are the versions that were submitted during the peer review process. They are not the versions of record or the final published versions. They should not be cited or distributed as the published version of this manuscript.

BMJ Open is an open access journal and the full, final, typeset and author-corrected version of record of the manuscript is available on our site with no access controls, subscription charges or pay-per-view fees (http://bmjopen.bmj.com).

If you have any questions on BMJ Open's open peer review process please email info.bmjopen@bmj.com

BMJ Open

Clinical code usage in UK general practice: a cohort study exploring 18 conditions over 14 years

Journal:	*BMJ Open*
Manuscript ID	bmjopen-2021-051456
Article Type:	Original research
Date Submitted by the Author:	19-Mar-2021

Complete List of Authors:	Zghebi, Salwa; The University of Manchester, NIHR School for Primary Care Research, Centre for Primary Care and Health Services Research, Manchester Academic Health Science Centre (MAHSC); The University of Manchester, Division of Population Health, Health Services Research and Primary Care, School of Health Sciences, Faculty of Biology, Medicine and Health, Manchester Academic Health Science Centre (MAHSC)<br>Reeves, David; The University of Manchester, NIHR School for Primary Care Research, Centre for Primary Care and Health Services Research, Manchester Academic Health Science Centre (MAHSC); The University of Manchester, Centre for Biostatistics, School of Health Sciences, Faculty of Biology, Medicine and Health, Manchester Academic Health Science Centre (MAHSC)<br>Grigoroglou, Christos; The University of Manchester, Manchester Centre for Health Economics, Division of Population Health, Health Services Research and Primary Care, Manchester Academic Health Science Centre (MAHSC)<br>McMillan, Brian; The University of Manchester, NIHR School for Primary Care Research, Centre for Primary Care and Health Services Research, Manchester Academic Health Science Centre (MAHSC); The University of Manchester, Division of Population Health, Health Services Research and Primary Care, School of Health Sciences, Faculty of Biology, Medicine and Health, Manchester Academic Health Science Centre (MAHSC)<br>Ashcroft, Darren; The University of Manchester, NIHR School for Primary Care Research, Centre for Primary Care and Health Services Research, Manchester Academic Health Science Centre (MAHSC); The University of Manchester, Centre for Pharmacoepidemiology and Drug Safety, School of Health Sciences, Faculty of Biology, Medicine and Health, Manchester Academic Health Science Centre (MAHSC)<br>Parisi, Rosa; The University of Manchester, NIHR School for Primary Care Research, Centre for Primary Care and Health Services Research, Manchester Academic Health Science Centre (MAHSC); The University of Manchester, Division of Informatics, Imaging, and Data Sciences, School of Health Sciences, Faculty of Biology, Medicine and Health, Manchester Academic Health Science Centre (MAHSC)<br>Kontopantelis, Evangelos; The University of Manchester, NIHR School for Primary Care Research, Centre for Primary Care and Health Services Research, Manchester Academic Health Science Centre (MAHSC); The University of Manchester, Division of Informatics, Imaging, and Data Sciences, School of Health Sciences, Faculty of Biology, Medicine and Health, Manchester Academic Health Science Centre (MAHSC)

Keywords:	PRIMARY CARE, PUBLIC HEALTH, Change management < HEALTH SERVICES ADMINISTRATION & MANAGEMENT

SCHOLARONE™
Manuscripts

I, the Submitting Author has the right to grant and does grant on behalf of all authors of the Work (as defined in the below author licence), an exclusive licence and/or a non-exclusive licence for contributions from authors who are: i) UK Crown employees; ii) where BMJ has agreed a CC-BY licence shall apply, and/or iii) in accordance with the terms applicable for US Federal Government officers or employees acting as part of their official duties; on a worldwide, perpetual, irrevocable, royalty-free basis to BMJ Publishing Group Ltd ("BMJ") its licensees and where the relevant Journal is co-owned by BMJ to the co-owners of the Journal, to publish the Work in this journal and any other BMJ products and to exploit all rights, as set out in our licence.

The Submitting Author accepts and understands that any supply made under these terms is made by BMJ to the Submitting Author unless you are acting as an employee on behalf of your employer or a postgraduate student of an affiliated institution which is paying any applicable article publishing charge ("APC") for Open Access articles. Where the Submitting Author wishes to make the Work available on an Open Access basis (and intends to pay the relevant APC), the terms of reuse of such Open Access shall be governed by a Creative Commons licence – details of these licences and which Creative Commons licence will apply to this Work are set out in our licence referred to above.

Other than as permitted in any relevant BMJ Author's Self Archiving Policies, I confirm this Work has not been accepted for publication elsewhere, is not being considered for publication elsewhere and does not duplicate material already published. I confirm all authors consent to publication of this Work and authorise the granting of this licence.

For peer review only

Clinical code usage in UK general practice: a cohort study exploring 18 conditions over 14 years

Salwa S Zghebi,[1,2*] David Reeves,[1,2,3] Christos Grigoroglou,[4] Brian McMillan,[1,2] Darren M Ashcroft,[1,5] Rosa Parisi,[1,6] Evangelos Kontopantelis[1,2,6]

1 NIHR School for Primary Care Research, Centre for Primary Care and Health Services Research, Manchester Academic Health Science Centre (MAHSC), The University of Manchester, Manchester, UK.

2 Division of Population Health, Health Services Research and Primary Care, School of Health Sciences, Faculty of Biology, Medicine and Health, Manchester Academic Health Science Centre (MAHSC), The University of Manchester, Manchester, UK.

3 Centre for Biostatistics, School of Health Sciences, Faculty of Biology, Medicine and Health, Manchester Academic Health Science Centre (MAHSC), The University of Manchester, Manchester, UK.

4 Manchester Centre for Health Economics, Division of Population Health, Health Services Research and Primary Care, Manchester Academic Health Science Centre (MAHSC), The University of Manchester, Manchester, UK.

5 Centre for Pharmacoepidemiology and Drug Safety , School of Health Sciences, Faculty of Biology, Medicine and Health, Manchester Academic Health Science Centre (MAHSC), The University of Manchester, Manchester, UK.

6 Division of Informatics, Imaging, and Data Sciences, School of Health Sciences, Faculty of Biology, Medicine and Health, Manchester Academic Health Science Centre (MAHSC), The University of Manchester, Manchester, UK.

*Correspondence to:
Dr Salwa Zghebi, NIHR School for Primary Care Research, Centre for Primary Care and Health Services Research, Williamson Building, University of Manchester, Manchester M13 9PL, UK. E-mail: salwa.zghebi@manchester.ac.uk

Number of words (Abstract): 291
Number of words (Main text): 4,000
Number of Figures: 5

For peer review only

Abstract

Objectives

To assess the diagnostic Read code usage for 18 conditions by examining their frequency and diversity in UK primary care between 2000 and 2013.

Design

Population-based cohort study

Setting

684 UK general practices contributing data to the Clinical Practice Research Datalink (CPRD) GOLD.

Participants

Patients with clinical codes for at least one of: asthma, chronic obstructive pulmonary disease (COPD), diabetes, hypertension, coronary heart disease, atrial fibrillation, heart failure, stroke, hypothyroidism, chronic kidney disease, learning disability (LD), depression, dementia, epilepsy, severe mental illness (SMI), osteoarthritis, osteoporosis, and cancer.

Primary and secondary outcome measures

For the frequency ranking of clinical codes, canonical correlation analysis was applied to 1-, 3-, and 5-year correlations of clinical code usage. Three measures of diversity (Shannon entropy index of diversity, richness, and evenness) were used to quantify changes in incident and total clinical codes.

Results

Overall, all examined conditions except LD, showed positive monotonic correlation. Hypertension, hypothyroidism, osteoarthritis, and SMI codes' usage had high 5-year correlation. The codes' usage diversity remained stable overall throughout the study period. Cancer, diabetes, and SMI had the highest richness (code lists need time to define) unlike atrial fibrillation, hypothyroidism, and LD. SMI and hypothyroidism (high vs. low richness, respectively) can last for 5 years, whereas, cancer/diabetes and LD (high vs. low richness) only last for 2 years.

Conclusions

This is an underreported research area and the findings suggest the codes' usage diversity for most conditions remained overall stable throughout the study period. Generated mental health code lists can last for a long time unlike cardiometabolic conditions and cancer. Adopting more consistent and less diverse coding would help improve data quality in primary care. Future research is needed following the transfer to the Systematized Nomenclature of Medicine Clinical Terms (SNOMED CT) coding.

Keywords

Primary care, clinical codes, electronic health records, QOF, Quality and Outcomes Framework.

For peer review only - http://bmjopen.bmj.com/site/about/guidelines.xhtml

Strengths and limitations of this study

- Our study presents a contemporary longitudinal analysis of clinical code usage in UK primary care, addressing an underreported research area.
- Our findings are relevant to clinical practice as we examined 18 physical and mental conditions as recorded in primary care over 14 years, using data from a large nationally representative database.
- Given the design of the recorded electronic health records, we may have missed some patients with these 18 conditions (such as patients not registered with general practices) which may have affected the observed patterns of clinical code usage.
- Our analysis used CPRD GOLD data, which are obtained from clinical practices with the VISION clinical system, and EMIS and SystmOne practices will be using somewhat different diagnostic codes.

For peer review only

For peer review only - http://bmjopen.bmj.com/site/about/guidelines.xhtml

Introduction

The use of electronic health records (EHRs) has increased rapidly over the last three decades.[1] This has enabled researchers from various disciplines to examine cross-sectional and longitudinal trends of large population medical records to address many clinical research questions. EHRs are increasingly used for clinical management, clinical audits and research with real-world data, applying cross-sectional to longitudinal study designs to address descriptive epidemiology, pharmacoepidemiology, interventions evaluation, and risk prediction modelling.[2,3] The available routinely collected data are far from perfect, but they provide a wealth of high-quality information on patients' clinical conditions, referrals, and medication usage,[4] informing important components of clinical practice such as clinical decision-making.

Since the beginning of medical computing systems usage from early 1970s,[5,6] the UK's primary care systems became fully computerised by 2003.[7,8] This transition was facilitated by Read codes, a comprehensive computerised semi-hierarchical clinical classification system designed for use in EHRs, which are still in use in the UK.[9] These were originally developed by a clinician, Dr James Read, in the early 1980's and became the main coding system for clinical data in the UK from the mid-1990s, succeeding the Oxford Medical Information System (OXMIS) codes that were the most widely used system throughout the 1980s.[10-12] However, the Systematized Nomenclature of Medicine Clinical Terms (SNOMED CT), a systematically organised collection of medical terms, is being rolled out in general practices in a phased approach from April 2018 to replace Read codes, and it includes symptoms, diagnoses, procedures, family history, allergies, and devices.[13,14] With evident increasing complexity of most healthcare disciplines,[15] such clinical terminologies make collated patient records more manageable in clinical practice settings.[16,17] To support users, national standards and guidelines are available on the use of clinical coding.[14,18] Several UK primary care electronic databases exist and are managed by different and varying computer software systems (EMIS, Vision and SystmOne), with Read codes still being the most common system through which to capture primary care clinical information. In the UK, the largest primary care databases available for research purposes include the Clinical Practice Research Datalink (CPRD), The Health Improvement Network (THIN), ResearchOne, and QResearch.[2,8]

For peer review only - http://bmjopen.bmj.com/site/about/guidelines.xhtml

In April 2004, the UK National Health Service (NHS) introduced the Quality and Outcomes Framework (QOF), a voluntary reward and incentive programme to reward UK general practices providing high-quality care based on a range of evidence-based clinical indicators, for example, management of common chronic conditions such as diabetes and asthma.[19,20,21]

Despite the fact that clinical coding is a key point in the daily functionality of routine clinical practice, studies investigating their usage in real-world electronic databases are limited, although the observed variation in coding practice between clinicians.[22] The use of codes is a fundamental aspect of analyses of EHRs, involving a considerable amount of work, through which researchers extract a final dataset to analyse. Clinical codes are commonly used and disseminated in the form of code lists which are compiled according to the purpose, such as diagnostic codes, or family history codes (Table S1). Accurate (high specificity and sensitivity) code lists are imperative in obtaining reliable data on exposures, covariates, and outcomes. Previous systematic reviews have reported overall high accuracy of discharge coding (completed by clinical and/or administrative staff) in UK EHRs data that is improving over time, where in one review accuracy was defined as the agreement between the codes allocated after independently assessing clinical notes (acting as a 'gold' standard) and those recorded on EHRs.[23,24] However, clinical practice changes over time, at varying degrees for different conditions, which is reflected in coding practice with new codes being introduced and others made redundant.

Thus, examining and quantifying the changes in clinical code usage over time is important, since alterations in usage that have not been considered, can have important implications for the analysis of EHRs, resource allocation and may inform public health policy. An example for EHR analysis implication, the use of a two-year old code-list for a given medical condition may or may not be a problem, depending on how much clinical practice has changed over time for that condition. This change in clinical practice may be driven by policy changes, such as better reimbursement for keeping a register of certain conditions. A study examining the variation in clinical code use in UK primary care using six clinical terms, found that searches for the same clinical term across four different computer systems resulted in different results e.g. the mean number of codes/list ranging between 12.7-35.2 codes.[25] This highlighted the need for a more consistent system of code usage, with a recommendation to replace primary care code lists with shorter lists and fewer number of coding choices.[25] In this study, we used data from

For peer review only - http://bmjopen.bmj.com/site/about/guidelines.xhtml

the UK CPRD GOLD database to examine the: 1) frequency ranking of diagnostic clinical codes for 18 physical and mental health conditions; 2) changes in the usage of individual clinical codes (incident vs. total codes) for these conditions between 2000-2013 covering the period before and after the launch of the QOF.

Methods

Data source and study design

We used data from the GOLD database of the UK Clinical Practice Research Datalink (CPRD), which comprises of data from contributing anonymised general practices using the VISION clinical computer system.[26] The CPRD is one of the world's largest longitudinal electronic medical databases providing anonymised data from primary care, and is broadly representative of the UK population.[8,27] The CPRD is structured to provide data on clinical information, referrals, consultations, immunisation, tests and prescribed therapies. Up to July 2013, the CPRD held data for 11.3 million patients registered in 674 general practices. Of these, 4.4 million were active patients (representing 6.9% of the total UK population), and 6.9 million records represent inactive patients (people who have died or are no longer registered with a participating general practice).[27]

Using financial year intervals between 01/04/2000-31/03/2013, we examined the changes in the use of diagnostic clinical codes for 18 exemplar medical conditions in UK practices: asthma, chronic obstructive pulmonary disease (COPD), diabetes (DM) both types, hypertension (HT), coronary heart disease (CHD), atrial fibrillation (AF), heart failure (HF), stroke, hypothyroidism, chronic kidney disease (CKD), learning disability (LD), depression, dementia, epilepsy, severe mental illness (SMI), osteoarthritis, osteoporosis, and cancer. The diabetes codes included those with complications if clearly linked to diabetes, such as 'type 2 diabetes mellitus with nephropathy' (Table S1). The selected conditions, apart from osteoarthritis, were included in the QOF scheme from 2004, whereas AF, CKD, dementia, depression, and LD were incentivised from 2006, and osteoporosis incentivised from 2012. This allowed us to examine and compare QOF conditions (incentivised at different stages) plus a condition not part of the QOF (osteoarthritis).

For peer review only - http://bmjopen.bmj.com/site/about/guidelines.xhtml

The clinical codes used to define the examined conditions are listed in the Clinical Codes online repository.[28] Each condition was examined as an incident code (using codes to identify new cases) and total codes (incident and prevalent cases) for each year during the study period.

Data analysis

To examine the consistency of clinical code use across time, we applied canonical correlation analysis (CAA) to estimate 1-year (e.g. 2006 to 2007), 3-year (e.g. 2006 to 2009), and 5-yearly canonical correlations (e.g. 2006 to 2011) for clinical code usage. CCA is a descriptive multivariable method that provides a measure of the canonical correlation (CC) between two groups of variables. CCA finds the best linear combinations maximizing the correlation (γ_1) between p variables in group 1 and q variables in group 2, where the variables are measured across a common set of units (e.g. general practices): [29]

$$Y^1 = (Y_1^1, \cdots, Y_p^1) \text{ (1)}$$

$$Y^2 = (Y_1^2, \cdots, Y_q^2) \text{ (2)}$$

Where Y^1 represents the set of p outcomes in group 1, and Y^2 the set of q outcomes in group 2. Consider the two linear combinations $\alpha' Y^1$ and $b' Y^2$, where α' is a $p\ x$ 1 vector of weighting coefficients and b' is likewise a $q\ x$ 1 vector; the CC (γ_1) is given by the choice of α' and b' that maximises the correlation between $\alpha' Y^1$ and $b' Y^2$: [29]

$$\gamma_1 = \max_{\text{a,b}} Corr(a'Y^1, b'Y^2) = \max_{\text{a,b}} \frac{a'\Sigma_{12}b}{\sqrt{a'\Sigma_{11}ab'\Sigma_{22}b}} \text{ (3)}$$

Where Σ_{11} = $Cov(Y^{(1)}, Y^{(1)})$; Σ_{12} = $Cov(Y^{(1)}, Y^{(2)})$; Σ_{21} = Σ_{12}'; and Σ_{22} = $Cov(Y^{(2)}, Y^{(2)})$.

In the present study, for a given practice the Y's represent the relative use of each clinical codes for a specific disease condition, expressed as a percentage of the total use across all codes for that condition. For example, for the 2006-2007 year-on-year diabetes correlation, group 1 would be the percentage frequency use of each clinical code for diabetes recorded in year 2006 and group 2 would be the corresponding percentage frequencies for each corresponding code recorded in year 2007, at the general practice level. The same applies for the 3-year and 5-year correlations.

For peer review only - http://bmjopen.bmj.com/site/about/guidelines.xhtml

We analysed percentage frequencies rather than frequency counts so as to remove any effects of variations in practice size or disease prevalence from the estimated CCs. CCs were calculated using the R statistical software ccaPP package[30] with the "Spearman" method, by which the weighted linear combinations $\alpha' Y^1$ and $b' Y^2$ for each year are ranked across practices prior to computation of the correlation. This method produces estimates that are more robust against model misspecification.[31]

Numbers of incident clinical codes could be small for some conditions and practices, which can lead to biased estimates of the CCs. To adjust for this, we applied the jackknife bias correction to the estimation of CCs for the incidence of clinical codes.[29]

For each of the 18 conditions, we also quantified changes in incident and total clinical code usage applying three measures of diversity. First, the Shannon entropy index of diversity (H), an equitability index which takes into account two dimensions of diversity (richness and evenness). The Shannon entropy index (H) was calculated as:

$$H = -\sum_i (p_i \, ln \, p_i)$$

Where p_i is the proportion of a clinical code *i* usage in a given year.

Second, we examined the richness (S) of clinical code usage by calculating the annual total number of incident and all codes used in a given year. Third, we estimated the evenness (J) of incident and total codes' usage, a measure of the relative usage of codes within a given year, i.e. evenness will be high if all codes have a similar distribution. J ranges between zero and one, with $J = 0$ indicating no evenness, and $J = 1$ indicating complete evenness. Evenness was calculated annually by dividing Shannon index (H) over the natural logarithm of richness (S).

$$J = \frac{H}{\ln(S)}$$

To simplify what these diversity measures imply, for example, if diabetes was represented using three diagnostic codes: code A (used 100 times), code B (used 175 times), and code C (used 350 times), then the proportions of codes would be 0.16, 0.28, and 0.56, respectively.

For peer review only - http://bmjopen.bmj.com/site/about/guidelines.xhtml

Shannon's entropy index (H) will be = -1*((0.16*ln0.16) + (0.28*ln0.28) + (0.56*ln0.56)) = 0.97; richness (S) = 3; and evenness (J) = 0.97/ln (3) = 0.88. All analyses were conducted using R software,[32] and were visualised using the ggplots2 package.

Patient and Public Involvement

No patients or members of the public were involved in this study.

Results

Clinical code frequency ranking

Correlation of code usage over a 3-year period showed a positive association for most conditions (Figure 1). Strong, overall positive, and monotonic correlation (CC >0.7) was observed for depression, hypertension, hypothyroidism, osteoarthritis, SMI, and stroke. Positive, monotonic but weaker associations were observed for CKD, epilepsy, and osteoporosis. Learning disability (LD) showed a non-monotonic function with fluctuations ranging between 0.4-1.0, and a notable decline after 2004 before increasing again from 2007. The 1-year and 5-year windows correlations showed similar overall trends, but the association was slightly decreasing as the window increases. Clinical conditions with the highest correlation levels were asthma, AF, cancer, CHD, depression, diabetes, hypertension, hypothyroidism, osteoarthritis, SMI, and stroke for the 1-year window (Figure S1). For the 5-year window, hypertension, hypothyroidism, osteoarthritis, and SMI codes' usage was overall highly correlated mainly in recent years (Figure S2). On the other hand, conditions with the lowest correlations (CC ≤0.6) were CKD and LD (for most years) for the 1-year window; and cancer, CHD, CKD, COPD, dementia, diabetes, epilepsy, HF, LD, and osteoporosis for the 5-year window.

Over a 3-year window, strong correlation for incident code usage (Jackknife bias corrected CC≥0.6) were observed for all examined conditions except CKD, epilepsy, and osteoporosis (Figure 2). Similarly, the 1-year and 5-year windows correlations showed similar trends but lower coefficients with longer windows (Figures S3 and S4, respectively).

For peer review only - http://bmjopen.bmj.com/site/about/guidelines.xhtml

Clinical code usage diversity

Data from 684 UK general practices contributing to the Clinical Practice Research Datalink (CPRD) GOLD were used. Overall, the diversity indices of code usage were stable over the study period for most conditions, but with wide confidence intervals. Higher entropy (H) indices were observed with cancer, diabetes, and SMI (H between 2-4), while the lowest levels were observed with LD and osteoporosis (H between 0-2) (Figure 3). Over time, the entropy index of code usage remained stable for most conditions, but increased gradually for asthma, COPD, diabetes, heart failure, and osteoporosis (primarily incident codes). Fluctuations and/or a separation between the incident and total codes trends were observed around 2006, mainly for AF, dementia, depression, CKD, and LD. The Shannon index (H) for incident codes had a similar trend to that for total codes for most conditions over time, except for cardiovascular disease (CVD) and diabetes where it exceeded total codes.

Across the examined conditions, the richness (S) of incident and total code usage (number of codes used) was the highest for cancer (>500 codes), diabetes and SMI (≥250 codes each) and the lowest for AF, hypothyroidism, and LD (S <100) (Figure 4). The trends however remained stable throughout the study period, except a small decrease for SMI codes and a decrease in cancer after a brief rise between 2000-2005. The difference between the number of incident and total codes for SMI, diabetes and cancer were evident (total codes more than incident codes), unlike in the other conditions where the S index was similar for both code categories.

The evenness (J) of both incident and total codes was overall stable and almost identical at least up to 2006, before total codes surpassed incident codes for most conditions except for depression and dementia where the J index for incident codes exceeded that of total codes (Figure 5). The two exceptions to this observation were LD and CKD. For LD, evenness was stable ~0.75 between 2000-2003, declined in 2004 before re-increasing from 2007 and returning to pre-2004 levels from 2011 onwards. For CKD, evenness dipped briefly around 2006-2007 and started to increase again from 2008 until the end of the study period (2013). Given the calculation formula, it is worth noting that the trends of entropy were similar to that of evenness for conditions with low richness, namely for AF, dementia, heart failure, hypertension, hypothyroidism, LD, osteoarthritis, and osteoporosis.

For peer review only - http://bmjopen.bmj.com/site/about/guidelines.xhtml

Discussion

Main findings

We assessed the clinical code usage for 18 conditions recorded in a large nationally representative UK EHR between 2000-2013. The results show overall strong positive monotonic correlation for all examined conditions except LD, that showed a fluctuating pattern during the study period. The CCs diminished over longer windows (5-year vs. 1-year window). Hypertension, hypothyroidism, osteoarthritis, and SMI had the highest 5-year correlation, mainly in later years of the study period.

The codes' usage entropy and evenness diversity measures remained overall stable throughout the study period for most conditions, except gradual increases over time for respiratory conditions, diabetes, HF, and osteoporosis. This increase in diversity may be partially due to the regular addition of new diagnostic codes and domains over time. For example, major revisions were introduced to the QOF in April 2006 resulting in the addition of new clinical areas and indicators.[33] As a consequence, CKD is among the conditions that it has been acknowledged to have benefited from these revisions as the CKD domain was added in 2006 reflected in improved recording in primary care from that year onwards.[34] For most conditions (except LD), evenness (indicating the abundance of codes in a sample) was overall ≥0.5 suggesting a uniform distribution of the codes. Cancer, diabetes, and SMI had the highest richness indices among all examined conditions.

Comparison with previous studies

Observational studies examining variations in clinical code usage are limited. A recent study examined the code usage of CVD between 2001-2015 in primary and secondary care records in England.[35] The study aimed to examine if temporal variability methods can identify changes in CVD recording by quantifying the differences of monthly distributions of the variables of interest: CVD status and sociodemographic variables. The study found variability in the frequency of CVD codes across time, potentially due to non-medical causes such as changes in coding used and coding guidelines e.g. changes in ICD (international classification of disease) coding in hospital records. Despite relevance, their approach (examining the

For peer review only - http://bmjopen.bmj.com/site/about/guidelines.xhtml

For peer review only

prevalence of CVD stratified by patient demographic variables) differs from our methods and hence the results are not directly comparable. In addition, we examined code usage for 18 conditions, including CVD, from UK general practices.

A study by Tai et al. examined the diversity of data entry screens in four clinical computer systems available in UK general practices and assessed its impact on the variation and quality of recorded clinical data for six exemplar conditions.[25] These were sore throat, tired all the time, depression, cystitis, type 2 diabetes, and myocardial infarction. The study concluded that the systems may contribute towards a diverse coding in primary care, suggesting the need to standardise clinical coding across systems and to adopt shorter and more restricted code lists to help improve data quality. This is an important issue for UK primary care, since the semi-structured and dynamic nature of Read codes often results in diverse and long clinical code lists. Additionally, some GP systems use CTV3 clinical codes and not Read codes resulting in the availability of two versions of clinical codes. SNOMED CT system, which is gradually being implemented across UK primary care from 2018, aimed to provide a single clinical terminology for effective and consistent exchange of clinical data across all NHS settings to help improve patient care and data analysis.[36] Being an international clinical terminology, SNOMED will allow the UK to participate in global health care research.

Our results showed that diabetes codes usage (types 1 and 2) had one of the highest richness index levels (number of codes used), while the diversity entropy index was steadily increasing over the study period, highlighting the increasing variety of diabetes codes used in primary care over time. This observation agrees with a previous study that examined the Read codes used to identify diabetes management in people with diabetes registered with 17 general practices in one locality in London.[11] That study concluded that a wide range of diabetes codes were used and that the number of people assigned each code differed across practices. This again indicates that an approach is required to standardise clinical code lists and thereby coding usage as much as possible, minimise clinical recording errors, and improve research robustness.

For peer review only - http://bmjopen.bmj.com/site/about/guidelines.xhtml

Implication of findings

Our findings shed additional light on the use of clinical codes in research. We found that hypertension, hypothyroidism, osteoarthritis, and SMI codes' usage are highly correlated over the 5-year window (i.e. the codes' usage was similar across years), whereas cancer, CHD, CKD, COPD, dementia, diabetes, epilepsy, HF, LD, and osteoporosis had lowest correlation over the same window. In terms of clinical code lists' size required to define a condition (richness), we found that conditions with the highest richness across the study period were cancer, diabetes, and SMI (between 250-875 codes), whereas AF, hypothyroidism, and LD had the lowest richness (<100 codes). Collectively, these findings indicate that diabetes, cancer, and SMI codes have high richness and need to be defined carefully and then they can either last for 5 years (SMI), or only to 2 years (diabetes and cancer). Whereas hypothyroidism has low code usage richness and can last for 5 years. This might be due to that diabetes is often a target of government initiatives, unlike hypothyroidism which is rarely a focus of such interventions.

The results suggest that defining cohorts of people with mental health conditions (SMI, depression) over time was less sensitive to the changes of code usage (up to 5 years old) compared with most cardiometabolic conditions and cancer.

The observed findings also suggest the need to adopt a more consistent and less diverse coding in primary care, as this will help improve data quality. Inconsistent use of clinical coding may result in people with the same condition not being flagged as having the condition,[22] which may have implications on searching and identifying these people for clinical and research purposes, or to identify people for shielding measures or those who are a priority for a vaccination as in the current COVID-19 pandemic. While acknowledging that SNOMED CT is gradually replacing Read codes in general practice care since April 2018, our findings are still relevant in documenting the clinical code usage over a long period where Read codes were the main UK coding system.

Also, the rapidly increasing complexity of healthcare systems[15] might play a role on the observed trends in code usage over time. In other words, code usage practices (e.g. the tendency of data enterer to use easily accessed and well-known codes) may be partially driven by personal and work factors in the complex healthcare systems such as limited time and organisational factors.

For peer review only - http://bmjopen.bmj.com/site/about/guidelines.xhtml

Strengths and limitations of the study

Our study has several strengths. Using a range of frequency and diversity measures, we present a contemporary longitudinal analysis of clinical code usage in UK primary care, while only a few existing studies have addressed this research area. We used data from a large nationally representative database, where the validity of recorded diagnostic coding has been acknowledged previously.[37] Additionally, the data quality is assumed to be high, as it is based on QOF clinical codes lists (except osteoarthritis). Our findings are relevant to clinical practice as we examined a broad range of prevalent physical and mental illnesses as recorded in primary care and considered the clinical implications of variations in clinical coding over 14 years.

Our study has also several limitations. Given the design of the recorded EHRs, we may have missed some patients with the examined conditions due to some unusual circumstances or settings, such as patients not registered with general practices (e.g. homeless people), which may have affected the observed patterns of clinical code usage. Also, analyses were not extended to examine ICD-10 clinical codes in secondary care setting (only available in England), as our aim was to focus on the usage of Read codes recorded in UK primary care visits as the main point of clinical care. CCA provides a single multivariate measure of correlation, thus simplifying interpretation compared to analysing each clinical code separately. However, the measure represents the maximum possible correlation between code use at two different time points and does not account for the code set being the same at both times, hence may over-represent actual agreement to some degree. Finally, we used CPRD GOLD which collects data from general practices using the VISION clinical system, and code usage will vary to some extent in general practices using EMIS or SystmOne. However, we would expect such variation to be low in chronic conditions incentivised through the QOF, with specific common code lists used by practices to ensure remuneration eligibility.

Conclusions

The code usage in UK primary care was overall stable for most of the examined chronic conditions managed in general practice between 2000-2013, but the changes were higher over longer time windows. Diabetes, cancer, and SMI codes need to be defined carefully but

For peer review only

SMI codes can last up to 5 years, whereas hypothyroidism has low richness and can last up for 5 years. Our study addresses an underreported research area and the findings suggest the need to adopt a more consistent and less diverse coding in primary care to help improve data quality and the use of recent codes for cardiometabolic conditions and cancer. More research is needed in this area following the full transfer to the SNOMED CT coding and to examine the code usage in secondary care settings.

Acknowledgments

The authors would like to thank Dr David A. Springate for extracting and analysing the data.

Funding

This study is funded by the National Institute for Health Research (NIHR) School for Primary Care Research (grant number 211). The views expressed are those of the authors and not necessarily those of the NIHR or the Department of Health and Social Care. The funder of the study had no role in study design, data collection, data analysis, data interpretation, or writing of the report. The lead author had full access to the data in the study, takes responsibility for its integrity and the data analysis, and had final responsibility for the decision to submit for publication.

Competing interests

DMA reports research grants from Abbvie, Almirall, Celgene, Eli Lilly, Novartis, UCB and the Leo Foundation. Other co-authors declare no competing interests.

Author contribution

EK and DAS designed the study. DAS extracted the data from all sources and performed the initial analyses. RP and SSZ validated and expanded the analyses. SSZ wrote the manuscript and EK, RP, DR, and CG critically edited the initial drafts. All authors contributed to interpretation of data and revised the paper for important intellectual content and agreed on the final version of the paper before submission. SSZ is the guarantor of this work and, as such, had full access to all the data in the study and takes responsibility for the integrity of the data and the accuracy of the data analysis.

For peer review only

For peer review only - http://bmjopen.bmj.com/site/about/guidelines.xhtml

Patient consent

Not applicable.

Data sharing

Clinical code lists are available from clinicalcodes.org. Electronic health records are, by definition, considered sensitive data in the UK by the Data Protection Act and cannot be shared via public deposition because of information governance restriction in place to protect patient confidentiality. Access to data is available only once approval has been obtained through the individual constituent entities controlling access to the data. The data can be requested via application to the Clinical Practice Research Datalink.

Transparency declaration

SSZ affirms that the manuscript is an honest, accurate, and transparent account of the study being reported; that no important aspects of the study have been omitted; and that any discrepancies from the study as planned (and, if relevant, registered) have been explained.

Ethical approval

This study is based on data from Clinical Practice Research Datalink (CPRD) obtained under licence from the UK Medicines and Healthcare products Regulatory Agency. The study was approved by the Independent Scientific Advisory Committee (ISAC) for MHRA Database Research (protocol number: 16_115). The data are provided by patients and collected by the NHS as part of their care and support. Generic ethical approval for observational research using CPRD with approval from ISAC has been granted by a Health Research Authority (HRA) Research Ethics Committee (East Midlands—Derby, REC reference number 05/MRE04/87).

Exclusive licence

The Corresponding Author has the right to grant on behalf of all authors and does grant on behalf of all authors, a worldwide licence (http://www.bmj.com/sites/default/files/BMJ%20Author%20Licence%20March%202013.doc) to the Publishers and its licensees in perpetuity, in all forms, formats and media (whether known now or created in the future).

For peer review only - http://bmjopen.bmj.com/site/about/guidelines.xhtml

References

1. Shephard E, Stapley S, Hamilton W. The use of electronic databases in primary care research. Family Practice 2011;**28**:352-54 doi: doi:10.1093/fampra/cmr039.
2. Casey JA, Schwartz BS, Stewart WF, et al. Using Electronic Health Records for Population Health Research: A Review of Methods and Applications. Annu Rev Public Health 2016;**37**:61–81 doi: 10.1146/annurev-publhealth-032315-021353.
3. Hippisley-Cox J, Coupland C, Brindle P. Development and validation of QRISK3 risk prediction algorithms to estimate future risk of cardiovascular disease: prospective cohort study. BMJ 2017;**357**:j2099 doi: doi: https://doi.org/10.1136/bmj.j2099
4. West-Strum D. Introduction to Pharmacoepidemiology. In: Yang Y, West-Strum D, eds. Understanding Pharmacoepidemiology. New York: McGraw Hill, 2011:7.
5. McMillan B ER, Brown B, Fitton R, Dickinson D. Primary Care Patient Records in the United Kingdom: Past, Present, and Future Research Priorities. J Med Internet Res 2018;**20**(12):e11293 doi: 10.2196/11293.
6. Benson T. Why general practitioners use computers and hospital doctors do not—Part 1: incentives. BMJ 2002;**325** (7372):1086–9 doi: 10.1136/bmj.325.7372.1086.
7. Millman A, Lee N, Brooke A. ABC of Medical Computing: Computers in general practice--I. BMJ 1995;**311**(800) doi: doi: https://doi.org/10.1136/bmj.311.7008.800
8. Kontopantelis E, Stevens R, Helms P, et al. Spatial distribution of clinical computer systems in primary care in England in 2016 and implications for primary care electronic medical record databases: a cross sectional population study. BMJ Open 2018;**8**:e020738 doi: 10.1136/bmjopen-2017-020738.
9. NHS Digital. Read Coded Clinical Terms. Secondary Read Coded Clinical Terms. 30 May 2019. https://www.datadictionary.nhs.uk/web_site_content/supporting_information/clinical_coding/read_coded_clinical_terms.asp?shownav=1.
10. Booth N. What are the Read Codes? Health Libraries Review 1994;**11**(3):177-82.
11. Gray J, Orr D, Majeed A. Use of Read codes in diabetes management in a south London primary care group: implications for establishing disease registers. BMJ 2003;**326**(1130) doi: https://doi.org/10.1136/bmj.326.7399.1130.
12. Benson T. The history of the Read codes: the inaugural James Read Memorial Lecture 2011. Informatics in Primary Care 2011;**19**:173–82.
13. NHS Digital. SNOMED CT. Secondary SNOMED CT 17 January 2020 2020. https://digital.nhs.uk/services/terminology-and-classifications/snomed-ct.
14. NHS Digital. Terminology and Classifications. Secondary Terminology and Classifications. 23 May 2019. https://digital.nhs.uk/services/terminology-and-classifications.
15. Plsek PE, Greenhalgh T. The challenge of complexity in health care. BMJ 2001;**323**(625).
16. Williams R, Brown B, Kontopantelis E, et al. Term sets: A transparent and reproducible representation of clinical code sets. Plos One 2019;**14**(2):e0212291 doi: https://doi.org/10.1371/journal.pone.0212291.
17. Watson N. Using clinical coding systems to best effect in electronic records. Secondary Using clinical coding systems to best effect in electronic records. 2001.

For peer review only - http://bmjopen.bmj.com/site/about/guidelines.xhtml

https://www.guidelinesinpractice.co.uk/using-clinical-coding-systems-to-best-effect-in-electronic-records/305085.article.
18. The Joint Computing Group of the General Practitioners Committee and the Royal College of General Practitioners. Good practice guidelines for general practice electronic patient records (version 3) - Guidance for GPs, 2003.
19. Lester H, Campbell S. Developing Quality and Outcomes Framework (QOF) indicators and the concept of 'QOFability'. Quality in Primary Care 2010;**18**:103-9.
20. NHS Digital. Quality and Outcome Framework business rules. Secondary Quality and Outcome Framework business rules 31 May 2019. https://digital.nhs.uk/data-and-information/data-collections-and-data-sets/data-collections/quality-and-outcomes-framework-qof.
21. Campbell SM, Reeves D, Kontopantelis E, et al. Effects of Pay for Performance on the Quality of Primary Care in England. NEJM 2009;**361**:368-78 doi: DOI: 10.1056/NEJMsa0807651
22. Tate AR, Dungey S, Glew S, et al. Quality of recording of diabetes in the UK: how does the GP's method of coding clinical data affect incidence estimates? Cross-sectional study using the CPRD database. BMJ Open 2017;**7**:e012905 doi: doi: 10.1136/bmjopen-2016-012905.
23. Burns EM, Rigby E, Mamidanna R, et al. Systematic review of discharge coding accuracy. Journal of Public Health 2012;**34**(1):138–48.
24. Campbell SE, Campbell MK, Grimshaw JM, et al. Systematic review of discharge coding accuracy. Journal of Public Health 2001;**23**(3):205–11.
25. Tai TW, Anandarajah S, Dhoul N, et al. Variation in clinical coding lists in UK general practice: a barrier to consistent data entry? Informatics in Primary Care 2007;**15**:143–50.
26. Medicines & Healthcare products Regulatory Agency (MHRA). Clinical Practice Research Datalink. Secondary Clinical Practice Research Datalink 2021. https://www.cprd.com/.
27. Herrett E, Gallagher AM, Bhaskaran K, et al. Data Resource Profile: Clinical Practice Research Datalink (CPRD). International Journal of Epidemiology 2015;**44**(3):827-36 doi: 10.1093/ije/dyv098.
28. Springate DA, Kontopantelis E, Ashcroft DM, et al. ClinicalCodes: An Online Clinical Codes Repository to Improve the Validity and Reproducibility of Research Using Electronic Medical Records. PLoS ONE 2014;**9**(6):e99825 doi: DOI: 10.1371/journal.pone.0099825.
29. Lee H-S. Canonical Correlation Analysis Using Small Number of Samples. Communications in Statistics - Simulation and Computation 2007;**36**(5):973-85 doi: 10.1080/03610910701539443.
30. Alfons A, Croux C, Filzmoser P. Robust maximum association between data sets: The R Package ccaPP. Austrian Journal of Statistics 2016;**45**(1):71–79 doi: https://doi.org/10.17713/ajs.v45i1.90.
31. Alfons A, Croux C, Filzmoser P. Robust Maximum Association Estimators. 2017;**112**(515):436-45 doi: https://doi.org/10.1080/01621459.2016.1148609.
32. R Core Team. R: A language and environment for statistical computing. R Foundation for Statistical Computing. Secondary R: A language and environment for statistical computing. R Foundation for Statistical Computing 2017. https://www.r-project.org/.
33. Calvert M, Shankar A, McManus RJ, et al. Effect of the quality and outcomes framework on diabetes care in the United Kingdom: retrospective cohort study. BMJ 2009;**338**:b1870.
34. Nihat A, de Lusignan S, Thomas N, et al. What drives quality improvement in chronic kidney disease (CKD) in primary care: process evaluation of the Quality Improvement in Chronic Kidney Disease (QICKD) trial. BMJ Open 2016;**6**(e008480) doi: 10.1136/bmjopen-2015-008480.

For peer review only - http://bmjopen.bmj.com/site/about/guidelines.xhtml

35. Rockenschaub P, Nguyen V, Aldridge RW, et al. Data-driven discovery of changes in clinical code usage over time: a case-study on changes in cardiovascular disease recording in two English electronic health records databases (2001–2015). BMJ Open 2020;**10**(e034396) doi: doi:10.1136/bmjopen-2019-034396.
36. NHS Digital. SNOMED CT implementation in primary care. Secondary SNOMED CT implementation in primary care 21/03/2019 2019. https://digital.nhs.uk/services/terminology-and-classifications/snomed-ct/snomed-ct-implementation-in-primary-care.
37. Khan NF, Harrison SE, Rose PW. Validity of diagnostic coding within the General Practice Research Database: a systematic review. British Journal of General Practice 2010;**60**(572):e128-36 doi: 10.3399/bjgp10X483562.

For peer review only

For peer review only - http://bmjopen.bmj.com/site/about/guidelines.xhtml

Figure legends

Figure 1 Canonical correlations using 3-year window of clinical code usage for 18 mental and physical conditions

CHD, coronary heart disease; CKD, chronic kidney disease; COPD, chronic obstructive pulmonary disease; DM, diabetes mellitus; HF, heart failure; HT, hypertension; SMI, severe mental illness.

Figure 2 Bias-corrected canonical correlations (95%CI) using 3-year window (incident codes) for 18 mental and physical conditions

CHD, coronary heart disease; CKD, chronic kidney disease; COPD, chronic obstructive pulmonary disease; DM, diabetes mellitus; HF, heart failure; HT, hypertension; SMI, severe mental illness.
Incident code: a clinical code indicating new (incident) cases.

Figure 3 Entropy (95% CI) of incident and all clinical codes usage for 18 mental and physical conditions

CHD, coronary heart disease; CKD, chronic kidney disease; COPD, chronic obstructive pulmonary disease; DM, diabetes mellitus; HF, heart failure; HT, hypertension; SMI, severe mental illness.
Incident code: a clinical code indicating new (incident) cases. All codes: any diagnostic clinical code for the condition incident and prevalent cases).

Figure 4 Richness of incident and all clinical codes usage for 18 mental and physical conditions

CHD, coronary heart disease; CKD, chronic kidney disease; COPD, chronic obstructive pulmonary disease; DM, diabetes mellitus; HF, heart failure; HT, hypertension; SMI, severe mental illness.
Incident code: a clinical code indicating new (incident) cases. All codes: any diagnostic clinical code for the condition incident and prevalent cases).

Figure 5 Evenness (95% CI) of incident and all clinical codes usage for 18 mental and physical conditions

CHD, coronary heart disease; CKD, chronic kidney disease; COPD, chronic obstructive pulmonary disease; DM, diabetes mellitus; HF, heart failure; HT, hypertension; SMI, severe mental illness.
Incident code: a clinical code indicating new (incident) cases. All codes: any diagnostic clinical code for the condition incident and prevalent cases).

For peer review only

For peer review only - http://bmjopen.bmj.com/site/about/guidelines.xhtml

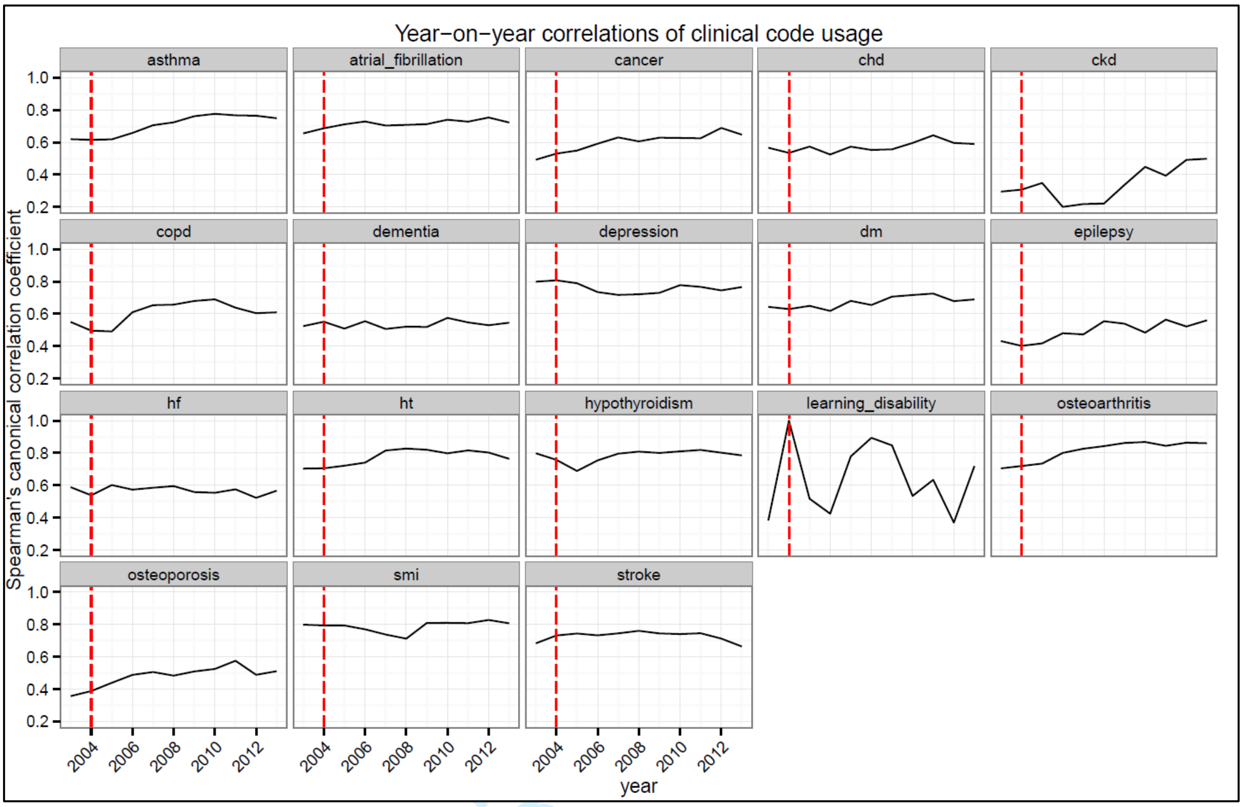

For peer review only

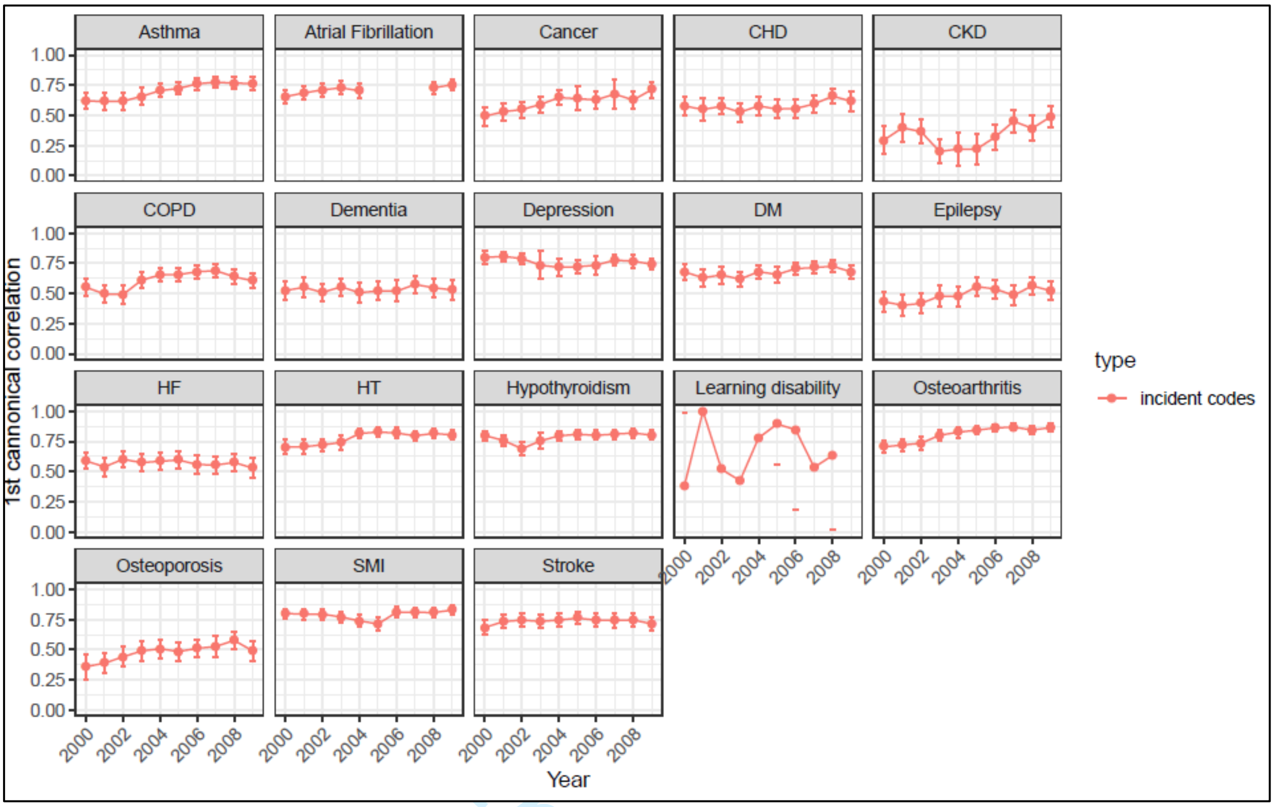

For peer review only

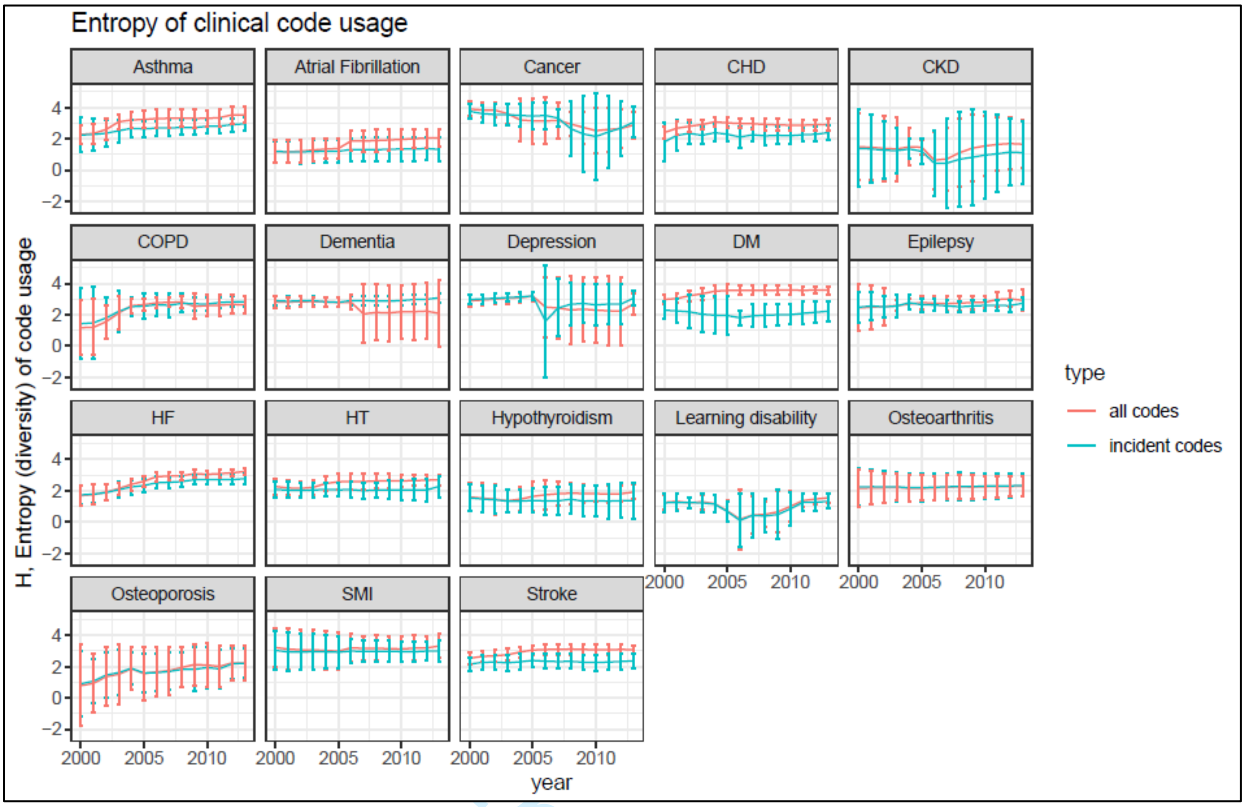

For peer review only

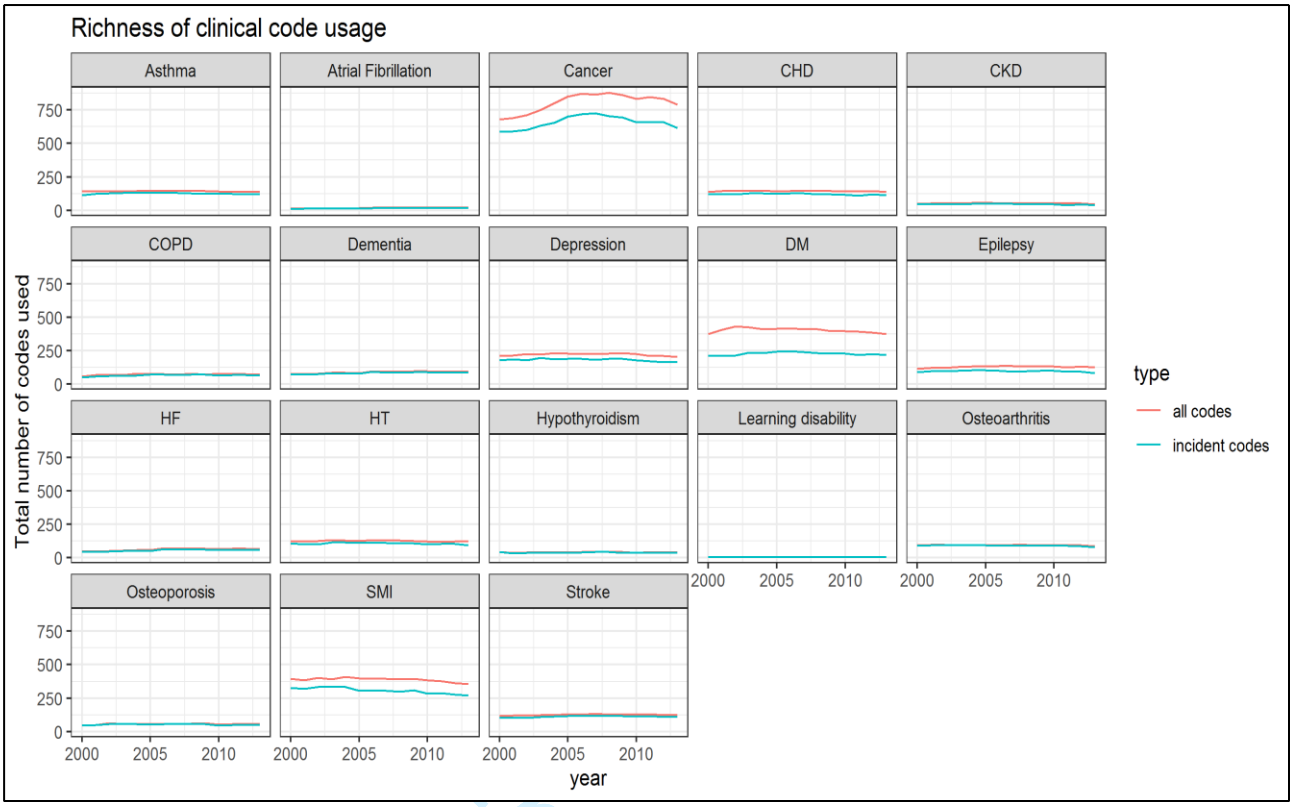
Richness of clinical code usage
Asthma
Atrial Fibrillation
Cancer
CHD
CKD
COPD
Dementia
Depression
DM
Epilepsy
HF
HT
Hypothyroidism
Learning disability
Osteoarthritis
Osteoporosis
SMI
Stroke
Total number of codes used
0
250
500
750
2000
2005
2010
year
type
all codes
incident codes

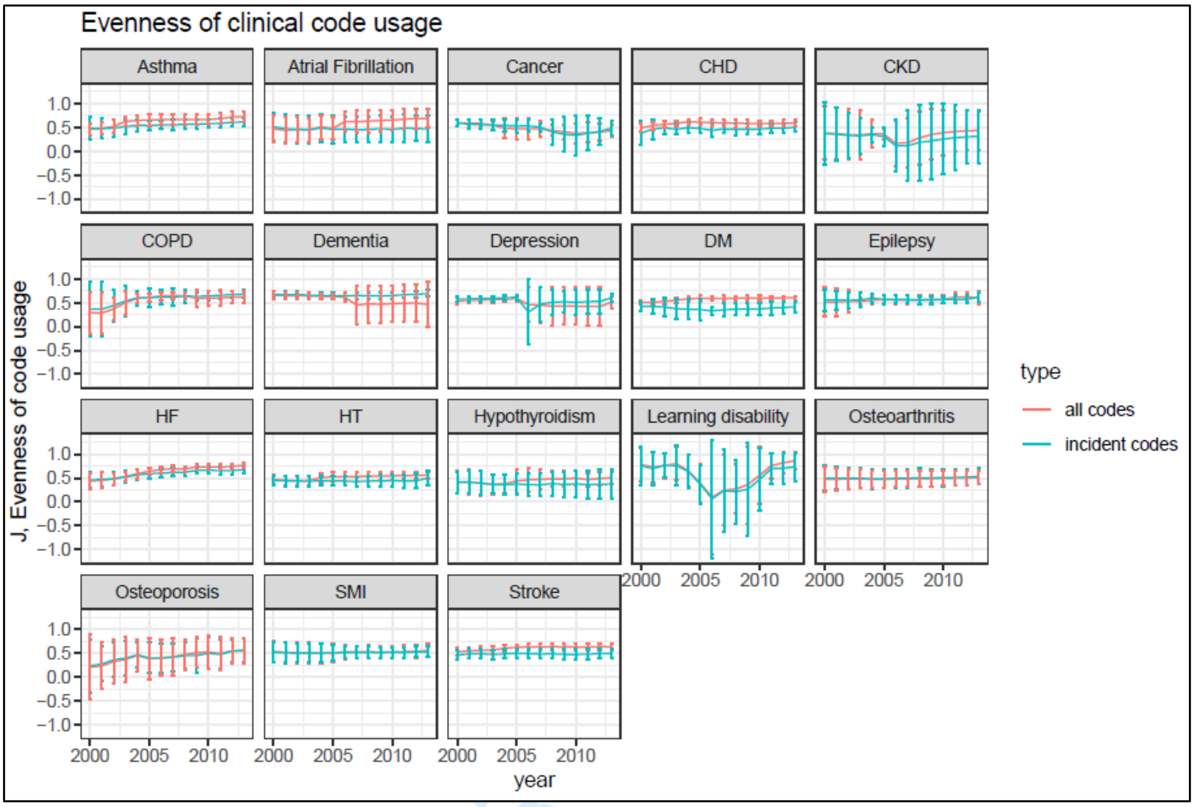
Evenness of clinical code usage
Asthma
Atrial Fibrillation
Cancer
CHD
CKD
COPD
Dementia
Depression
DM
Epilepsy
HF
HT
Hypothyroidism
Learning disability
Osteoarthritis
Osteoporosis
SMI
Stroke
1.0
0.5
0.0
−0.5
−1.0
J, Evenness of code usage
2000
2005
2010
year
type
all codes
incident codes

Supplementary data to "Clinical code usage in UK general practice: a cohort study exploring 18 conditions over 14 years", Zghebi et al. 2021.

Supplementary data

For peer review only

For peer review only - http://bmjopen.bmj.com/site/about/guidelines.xhtml

Supplementary data to "Clinical code usage in UK general practice: a cohort study exploring 18 conditions over 14 years", Zghebi et al. 2021.

Table S1 Diabetes Read code list

	Code	Coding system	Description
1.	66A3.00	Read	Diabetic on diet only
2.	66A4.00	Read	Diabetic on oral treatment
3.	66A5.00	Read	Diabetic on insulin
4.	66AI.00	Read	Diabetic - good control
5.	66AJ.00	Read	Diabetic - poor control
6.	66AJ100	Read	Brittle diabetes
7.	66AJ.11	Read	Unstable diabetes
8.	66AJz00	Read	Diabetic - poor control NOS
9.	66AK.00	Read	Diabetic - cooperative patient
10.	66AL.00	Read	Diabetic-uncooperative patient
11.	66AV.00	Read	Diabetic on insulin and oral treatment
12.	C10..00	Read	Diabetes mellitus
13.	C100.00	Read	Diabetes mellitus with no mention of complication
14.	C100000	Read	Diabetes mellitus; juvenile type; no mention of complication
15.	C100011	Read	Insulin dependent diabetes mellitus
16.	C100100	Read	Diabetes mellitus; adult onset; no mention of complication
17.	C100111	Read	Maturity onset diabetes
18.	C100112	Read	Non-insulin dependent diabetes mellitus
19.	C100z00	Read	Diabetes mellitus NOS with no mention of complication
20.	C101.00	Read	Diabetes mellitus with ketoacidosis
21.	C101000	Read	Diabetes mellitus; juvenile type; with ketoacidosis
22.	C101100	Read	Diabetes mellitus; adult onset; with ketoacidosis
23.	C101y00	Read	Other specified diabetes mellitus with ketoacidosis
24.	C101z00	Read	Diabetes mellitus NOS with ketoacidosis
25.	C102.00	Read	Diabetes mellitus with hyperosmolar coma
26.	C102000	Read	Diabetes mellitus; juvenile type; with hyperosmolar coma
27.	C102100	Read	Diabetes mellitus; adult onset; with hyperosmolar coma
28.	C102z00	Read	Diabetes mellitus NOS with hyperosmolar coma
29.	C103.00	Read	Diabetes mellitus with ketoacidotic coma
30.	C103000	Read	Diabetes mellitus; juvenile type; with ketoacidotic coma
31.	C103100	Read	Diabetes mellitus; adult onset; with ketoacidotic coma
32.	C103y00	Read	Other specified diabetes mellitus with coma
33.	C103z00	Read	Diabetes mellitus NOS with ketoacidotic coma
34.	C104.00	Read	Diabetes mellitus with renal manifestation
35.	C104000	Read	Diabetes mellitus; juvenile type; with renal manifestation
36.	C104100	Read	Diabetes mellitus; adult onset; with renal manifestation
37.	C104y00	Read	Other specified diabetes mellitus with renal complications
38.	C104z00	Read	Diabetes mellitis with nephropathy NOS
39.	C105.00	Read	Diabetes mellitus with ophthalmic manifestation
40.	C105000	Read	Diabetes mellitus; juvenile type; + ophthalmic manifestation
41.	C105100	Read	Diabetes mellitus; adult onset; + ophthalmic manifestation
42.	C105y00	Read	Other specified diabetes mellitus with ophthalmic complicatn
43.	C105z00	Read	Diabetes mellitus NOS with ophthalmic manifestation
44.	C106.00	Read	Diabetes mellitus with neurological manifestation
45.	C106000	Read	Diabetes mellitus; juvenile; + neurological manifestation
46.	C106100	Read	Diabetes mellitus; adult onset; + neurological manifestation
47.	C106.12	Read	Diabetes mellitus with neuropathy

For peer review only - http://bmjopen.bmj.com/site/about/guidelines.xhtml

Supplementary data to "Clinical code usage in UK general practice: a cohort study exploring 18 conditions over 14 years", Zghebi et al. 2021.

48.	C106.13	Read	Diabetes mellitus with polyneuropathy
49.	C106y00	Read	Other specified diabetes mellitus with neurological comps
50.	C106z00	Read	Diabetes mellitus NOS with neurological manifestation
51.	C107.00	Read	Diabetes mellitus with peripheral circulatory disorder
52.	C107000	Read	Diabetes mellitus; juvenile +peripheral circulatory disorder
53.	C107100	Read	Diabetes mellitus; adult; + peripheral circulatory disorder
54.	C107.11	Read	Diabetes mellitus with gangrene
55.	C107.12	Read	Diabetes with gangrene
56.	C107200	Read	Diabetes mellitus; adult with gangrene
57.	C107300	Read	IDDM with peripheral circulatory disorder
58.	C107400	Read	NIDDM with peripheral circulatory disorder
59.	C107z00	Read	Diabetes mellitus NOS with peripheral circulatory disorder
60.	C108.00	Read	Insulin dependent diabetes mellitus
61.	C108000	Read	Insulin-dependent diabetes mellitus with renal complications
62.	C108011	Read	Type I diabetes mellitus with renal complications
63.	C108012	Read	Type 1 diabetes mellitus with renal complications
64.	C108100	Read	Insulin-dependent diabetes mellitus with ophthalmic comps
65.	C108.11	Read	IDDM-Insulin dependent diabetes mellitus
66.	C108.12	Read	Type 1 diabetes mellitus
67.	C108.13	Read	Type I diabetes mellitus
68.	C108200	Read	Insulin-dependent diabetes mellitus with neurological comps
69.	C108211	Read	Type I diabetes mellitus with neurological complications
70.	C108212	Read	Type 1 diabetes mellitus with neurological complications
71.	C108300	Read	Insulin dependent diabetes mellitus with multiple complicatn
72.	C108400	Read	Unstable insulin dependent diabetes mellitus
73.	C108411	Read	Unstable type I diabetes mellitus
74.	C108500	Read	Insulin dependent diabetes mellitus with ulcer
75.	C108511	Read	Type I diabetes mellitus with ulcer
76.	C108600	Read	Insulin dependent diabetes mellitus with gangrene
77.	C108700	Read	Insulin dependent diabetes mellitus with retinopathy
78.	C108711	Read	Type I diabetes mellitus with retinopathy
79.	C108712	Read	Type 1 diabetes mellitus with retinopathy
80.	C108800	Read	Insulin dependent diabetes mellitus - poor control
81.	C108811	Read	Type I diabetes mellitus - poor control
82.	C108812	Read	Type 1 diabetes mellitus - poor control
83.	C108900	Read	Insulin dependent diabetes maturity onset
84.	C108911	Read	Type I diabetes mellitus maturity onset
85.	C108A00	Read	Insulin-dependent diabetes without complication
86.	C108B00	Read	Insulin dependent diabetes mellitus with mononeuropathy
87.	C108B11	Read	Type I diabetes mellitus with mononeuropathy
88.	C108C00	Read	Insulin dependent diabetes mellitus with polyneuropathy
89.	C108D00	Read	Insulin dependent diabetes mellitus with nephropathy
90.	C108D11	Read	Type I diabetes mellitus with nephropathy
91.	C108E00	Read	Insulin dependent diabetes mellitus with hypoglycaemic coma
92.	C108E11	Read	Type I diabetes mellitus with hypoglycaemic coma
93.	C108E12	Read	Type 1 diabetes mellitus with hypoglycaemic coma
94.	C108F00	Read	Insulin dependent diabetes mellitus with diabetic cataract
95.	C108F11	Read	Type I diabetes mellitus with diabetic cataract
96.	C108G00	Read	Insulin dependent diab mell with peripheral angiopathy
97.	C108H00	Read	Insulin dependent diabetes mellitus with arthropathy

For peer review only - http://bmjopen.bmj.com/site/about/guidelines.xhtml

Supplementary data to "Clinical code usage in UK general practice: a cohort study exploring 18 conditions over 14 years", Zghebi et al. 2021.

98.	C108H11	Read	Type I diabetes mellitus with arthropathy
99.	C108J00	Read	Insulin dependent diab mell with neuropathic arthropathy
100.	C108J12	Read	Type 1 diabetes mellitus with neuropathic arthropathy
101.	C108y00	Read	Other specified diabetes mellitus with multiple comps
102.	C108z00	Read	Unspecified diabetes mellitus with multiple complications
103.	C109.00	Read	Non-insulin-dependent diabetes mellitus
104.	C109000	Read	Non-insulin-dependent diabetes mellitus with renal comps
105.	C109011	Read	Type II diabetes mellitus with renal complications
106.	C109012	Read	Type 2 diabetes mellitus with renal complications
107.	C109100	Read	Non-insulin-dependent diabetes mellitus with ophthalm comps
108.	C109.11	Read	NIDDM - Non-insulin dependent diabetes mellitus
109.	C109111	Read	Type II diabetes mellitus with ophthalmic complications
110.	C109112	Read	Type 2 diabetes mellitus with ophthalmic complications
111.	C109.12	Read	Type 2 diabetes mellitus
112.	C109.13	Read	Type II diabetes mellitus
113.	C109200	Read	Non-insulin-dependent diabetes mellitus with neuro comps
114.	C109211	Read	Type II diabetes mellitus with neurological complications
115.	C109212	Read	Type 2 diabetes mellitus with neurological complications
116.	C109300	Read	Non-insulin-dependent diabetes mellitus with multiple comps
117.	C109400	Read	Non-insulin dependent diabetes mellitus with ulcer
118.	C109411	Read	Type II diabetes mellitus with ulcer
119.	C109412	Read	Type 2 diabetes mellitus with ulcer
120.	C109500	Read	Non-insulin dependent diabetes mellitus with gangrene
121.	C109511	Read	Type II diabetes mellitus with gangrene
122.	C109600	Read	Non-insulin-dependent diabetes mellitus with retinopathy
123.	C109611	Read	Type II diabetes mellitus with retinopathy
124.	C109612	Read	Type 2 diabetes mellitus with retinopathy
125.	C109700	Read	Non-insulin dependant diabetes mellitus - poor control
126.	C109711	Read	Type II diabetes mellitus - poor control
127.	C109712	Read	Type 2 diabetes mellitus - poor control
128.	C109900	Read	Non-insulin-dependent diabetes mellitus without complication
129.	C109A00	Read	Non-insulin dependent diabetes mellitus with mononeuropathy
130.	C109A11	Read	Type II diabetes mellitus with mononeuropathy
131.	C109B00	Read	Non-insulin dependent diabetes mellitus with polyneuropathy
132.	C109B11	Read	Type II diabetes mellitus with polyneuropathy
133.	C109C00	Read	Non-insulin dependent diabetes mellitus with nephropathy
134.	C109C11	Read	Type II diabetes mellitus with nephropathy
135.	C109C12	Read	Type 2 diabetes mellitus with nephropathy
136.	C109D00	Read	Non-insulin dependent diabetes mellitus with hypoglyca coma
137.	C109D11	Read	Type II diabetes mellitus with hypoglycaemic coma
138.	C109D12	Read	Type 2 diabetes mellitus with hypoglycaemic coma
139.	C109E00	Read	Non-insulin depend diabetes mellitus with diabetic cataract
140.	C109E11	Read	Type II diabetes mellitus with diabetic cataract
141.	C109E12	Read	Type 2 diabetes mellitus with diabetic cataract
142.	C109F00	Read	Non-insulin-dependent d m with peripheral angiopath
143.	C109F11	Read	Type II diabetes mellitus with peripheral angiopathy
144.	C109F12	Read	Type 2 diabetes mellitus with peripheral angiopathy
145.	C109G00	Read	Non-insulin dependent diabetes mellitus with arthropathy
146.	C109G11	Read	Type II diabetes mellitus with arthropathy
147.	C109G12	Read	Type 2 diabetes mellitus with arthropathy

For peer review only - http://bmjopen.bmj.com/site/about/guidelines.xhtml

Supplementary data to "Clinical code usage in UK general practice: a cohort study exploring 18 conditions over 14 years", Zghebi et al. 2021.

148.	C109H00	Read	Non-insulin dependent d m with neuropathic arthropathy
149.	C109H11	Read	Type II diabetes mellitus with neuropathic arthropathy
150.	C109H12	Read	Type 2 diabetes mellitus with neuropathic arthropathy
151.	C109J00	Read	Insulin treated Type 2 diabetes mellitus
152.	C109J11	Read	Insulin treated non-insulin dependent diabetes mellitus
153.	C109J12	Read	Insulin treated Type II diabetes mellitus
154.	C109K00	Read	Hyperosmolar non-ketotic state in type 2 diabetes mellitus
155.	C10C.00	Read	Diabetes mellitus autosomal dominant
156.	C10C.11	Read	Maturity onset diabetes in youth
157.	C10D.00	Read	Diabetes mellitus autosomal dominant type 2
158.	C10D.11	Read	Maturity onset diabetes in youth type 2
159.	C10E.00	Read	Type 1 diabetes mellitus
160.	C10E000	Read	Type 1 diabetes mellitus with renal complications
161.	C10E100	Read	Type 1 diabetes mellitus with ophthalmic complications
162.	C10E.11	Read	Type I diabetes mellitus
163.	C10E.12	Read	Insulin dependent diabetes mellitus
164.	C10E200	Read	Type 1 diabetes mellitus with neurological complications
165.	C10E300	Read	Type 1 diabetes mellitus with multiple complications
166.	C10E312	Read	Insulin dependent diabetes mellitus with multiple complicat
167.	C10E400	Read	Unstable type 1 diabetes mellitus
168.	C10E411	Read	Unstable type I diabetes mellitus
169.	C10E412	Read	Unstable insulin dependent diabetes mellitus
170.	C10E500	Read	Type 1 diabetes mellitus with ulcer
171.	C10E600	Read	Type 1 diabetes mellitus with gangrene
172.	C10E700	Read	Type 1 diabetes mellitus with retinopathy
173.	C10E800	Read	Type 1 diabetes mellitus - poor control
174.	C10E812	Read	Insulin dependent diabetes mellitus - poor control
175.	C10E900	Read	Type 1 diabetes mellitus maturity onset
176.	C10EA00	Read	Type 1 diabetes mellitus without complication
177.	C10EA11	Read	Type I diabetes mellitus without complication
178.	C10EB00	Read	Type 1 diabetes mellitus with mononeuropathy
179.	C10EC00	Read	Type 1 diabetes mellitus with polyneuropathy
180.	C10ED00	Read	Type 1 diabetes mellitus with nephropathy
181.	C10EE00	Read	Type 1 diabetes mellitus with hypoglycaemic coma
182.	C10EF00	Read	Type 1 diabetes mellitus with diabetic cataract
183.	C10EG00	Read	Type 1 diabetes mellitus with peripheral angiopathy
184.	C10EH00	Read	Type 1 diabetes mellitus with arthropathy
185.	C10EJ00	Read	Type 1 diabetes mellitus with neuropathic arthropathy
186.	C10EK00	Read	Type 1 diabetes mellitus with persistent proteinuria
187.	C10EL00	Read	Type 1 diabetes mellitus with persistent microalbuminuria
188.	C10EM00	Read	Type 1 diabetes mellitus with ketoacidosis
189.	C10EM11	Read	Type I diabetes mellitus with ketoacidosis
190.	C10EN00	Read	Type 1 diabetes mellitus with ketoacidotic coma
191.	C10EN11	Read	Type I diabetes mellitus with ketoacidotic coma
192.	C10EP00	Read	Type 1 diabetes mellitus with exudative maculopathy
193.	C10EQ00	Read	Type 1 diabetes mellitus with gastroparesis
194.	C10F.00	Read	Type 2 diabetes mellitus
195.	C10F000	Read	Type 2 diabetes mellitus with renal complications
196.	C10F011	Read	Type II diabetes mellitus with renal complications
197.	C10F100	Read	Type 2 diabetes mellitus with ophthalmic complications

For peer review only - http://bmjopen.bmj.com/site/about/guidelines.xhtml

Supplementary data to "Clinical code usage in UK general practice: a cohort study exploring 18 conditions over 14 years", Zghebi et al. 2021.

198.	C10F.11	Read	Type II diabetes mellitus
199.	C10F200	Read	Type 2 diabetes mellitus with neurological complications
200.	C10F300	Read	Type 2 diabetes mellitus with multiple complications
201.	C10F311	Read	Type II diabetes mellitus with multiple complications
202.	C10F400	Read	Type 2 diabetes mellitus with ulcer
203.	C10F500	Read	Type 2 diabetes mellitus with gangrene
204.	C10F511	Read	Type II diabetes mellitus with gangrene
205.	C10F600	Read	Type 2 diabetes mellitus with retinopathy
206.	C10F611	Read	Type II diabetes mellitus with retinopathy
207.	C10F700	Read	Type 2 diabetes mellitus - poor control
208.	C10F711	Read	Type II diabetes mellitus - poor control
209.	C10F900	Read	Type 2 diabetes mellitus without complication
210.	C10F911	Read	Type II diabetes mellitus without complication
211.	C10FA00	Read	Type 2 diabetes mellitus with mononeuropathy
212.	C10FB00	Read	Type 2 diabetes mellitus with polyneuropathy
213.	C10FB11	Read	Type II diabetes mellitus with polyneuropathy
214.	C10FC00	Read	Type 2 diabetes mellitus with nephropathy
215.	C10FC11	Read	Type II diabetes mellitus with nephropathy
216.	C10FD00	Read	Type 2 diabetes mellitus with hypoglycaemic coma
217.	C10FE00	Read	Type 2 diabetes mellitus with diabetic cataract
218.	C10FF00	Read	Type 2 diabetes mellitus with peripheral angiopathy
219.	C10FG00	Read	Type 2 diabetes mellitus with arthropathy
220.	C10FH00	Read	Type 2 diabetes mellitus with neuropathic arthropathy
221.	C10FJ00	Read	Insulin treated Type 2 diabetes mellitus
222.	C10FJ11	Read	Insulin treated Type II diabetes mellitus
223.	C10FK00	Read	Hyperosmolar non-ketotic state in type 2 diabetes mellitus
224.	C10FL00	Read	Type 2 diabetes mellitus with persistent proteinuria
225.	C10FL11	Read	Type II diabetes mellitus with persistent proteinuria
226.	C10FM00	Read	Type 2 diabetes mellitus with persistent microalbuminuria
227.	C10FN00	Read	Type 2 diabetes mellitus with ketoacidosis
228.	C10FP00	Read	Type 2 diabetes mellitus with ketoacidotic coma
229.	C10FQ00	Read	Type 2 diabetes mellitus with exudative maculopathy
230.	C10FR00	Read	Type 2 diabetes mellitus with gastroparesis
231.	C10G.00	Read	Secondary pancreatic diabetes mellitus
232.	C10G000	Read	Secondary pancreatic diabetes mellitus without complication
233.	C10y.00	Read	Diabetes mellitus with other specified manifestation
234.	C10y100	Read	Diabetes mellitus; adult; + other specified manifestation
235.	C10yy00	Read	Other specified diabetes mellitus with other spec comps
236.	C10yz00	Read	Diabetes mellitus NOS with other specified manifestation
237.	C10z.00	Read	Diabetes mellitus with unspecified complication
238.	C10z000	Read	Diabetes mellitus; juvenile type; + unspecified complication
239.	C10z100	Read	Diabetes mellitus; adult onset; + unspecified complication
240.	C10zy00	Read	Other specified diabetes mellitus with unspecified comps
241.	C10zz00	Read	Diabetes mellitus NOS with unspecified complication
242.	Cyu2.00	Read	[X]Diabetes mellitus
243.	Cyu2000	Read	[X]Other specified diabetes mellitus
244.	Cyu2300	Read	[X]Unspecified diabetes mellitus with renal complications
245.	L180500	Read	Pre-existing diabetes mellitus; insulin-dependent
246.	L180600	Read	Pre-existing diabetes mellitus; non-insulin-dependent
247.	L180X00	Read	Pre-existing diabetes mellitus; unspecified

For peer review only - http://bmjopen.bmj.com/site/about/guidelines.xhtml

Supplementary data to "Clinical code usage in UK general practice: a cohort study exploring 18 conditions over 14 years", Zghebi et al. 2021.

Figure S1 Canonical correlation using 1-year window of clinical code usage for 18 mental and physical conditions

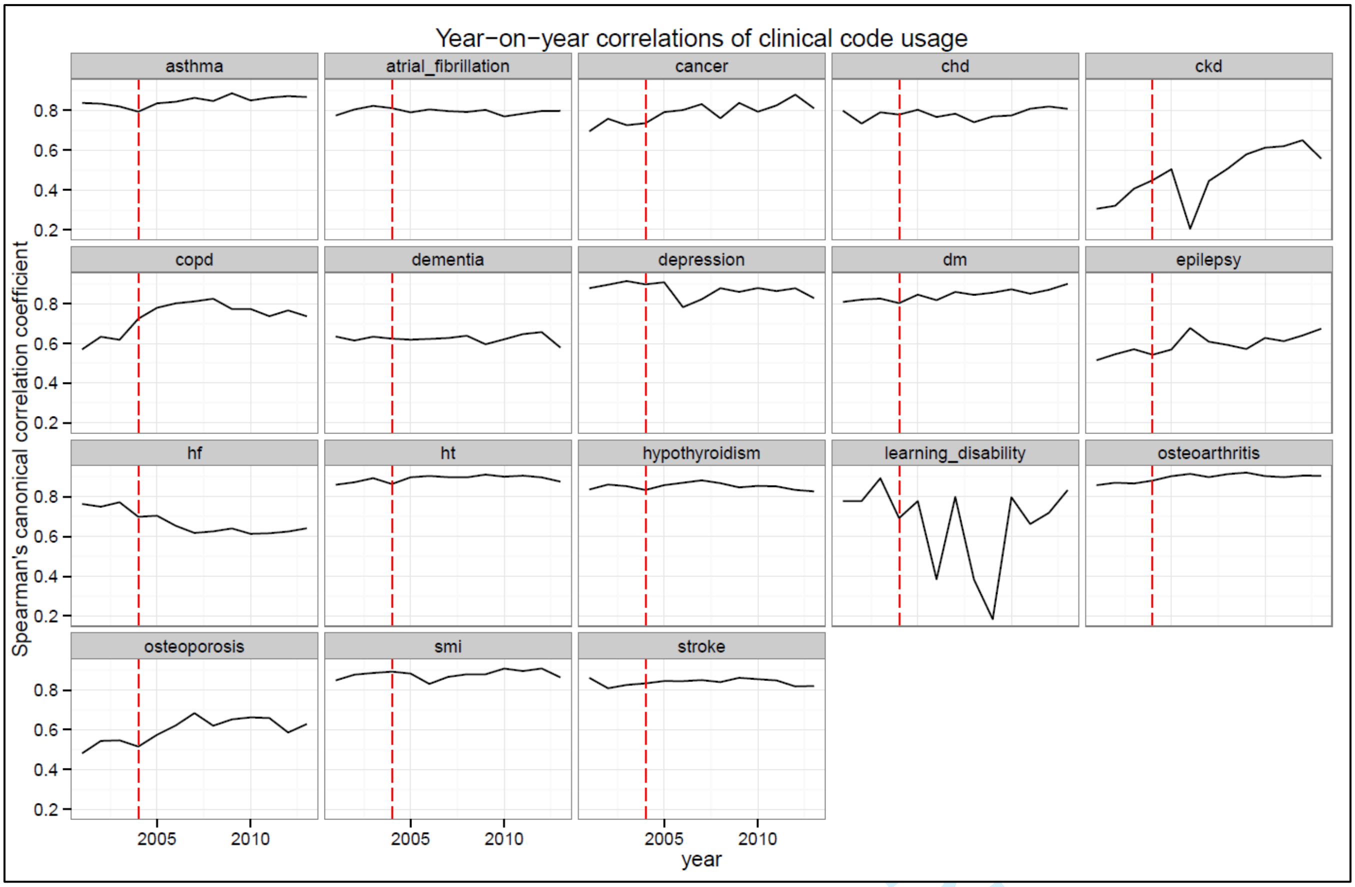

AF, atrial fibrillation; CHD, coronary heart disease; CKD, chronic kidney disease; COPD, chronic obstructive pulmonary disease; DM, diabetes mellitus; HF, heart failure; HT, hypertension; SMI, severe mental illness.

For peer review only - http://bmjopen.bmj.com/site/about/guidelines.xhtml

Supplementary data to "Clinical code usage in UK general practice: a cohort study exploring 18 conditions over 14 years", Zghebi et al. 2021.

Figure S2 Canonical correlation using 5-year window of clinical code usage for 18 mental and physical conditions

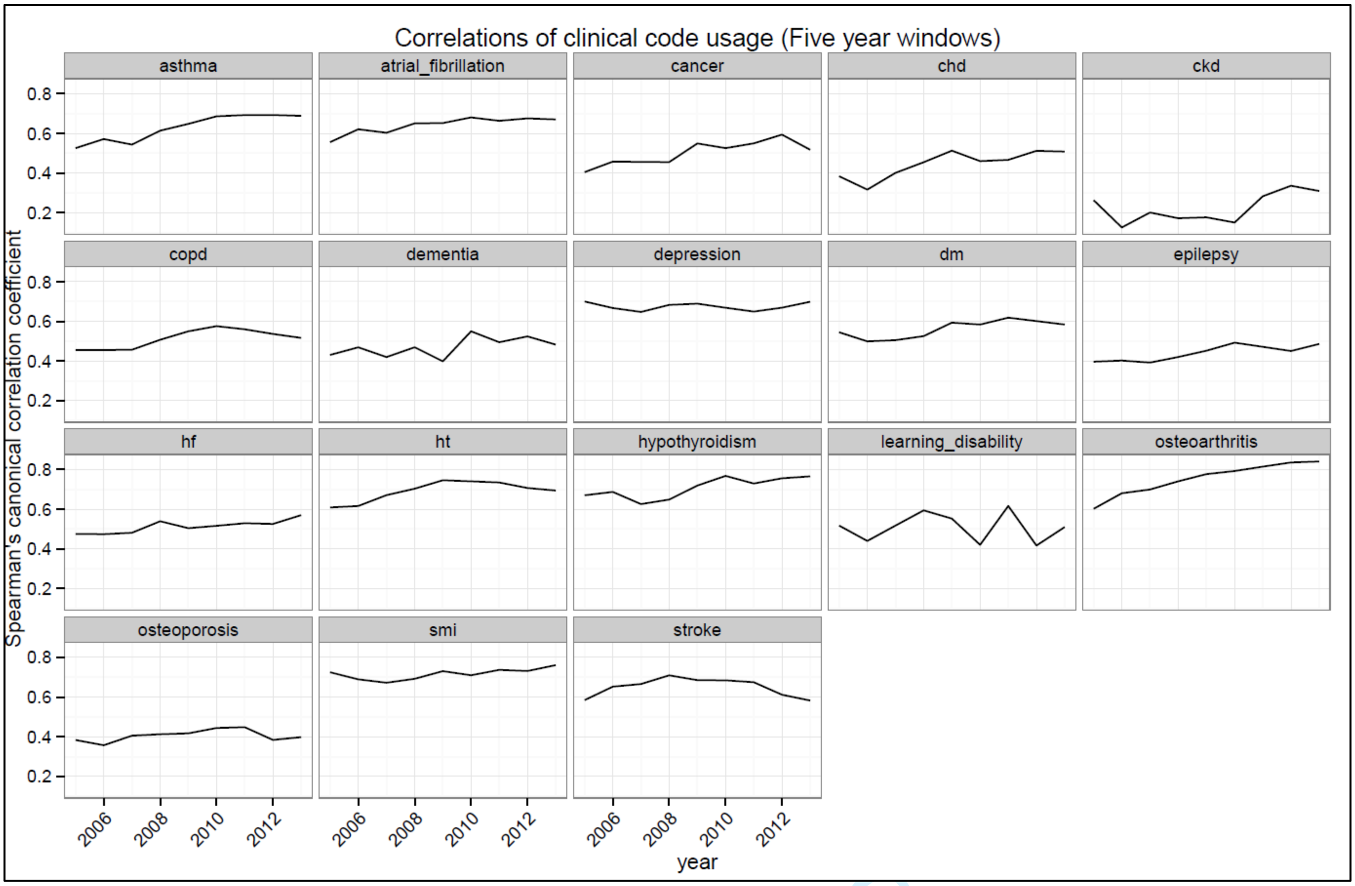

CHD, coronary heart disease; CKD, chronic kidney disease; COPD, chronic obstructive pulmonary disease; DM, diabetes mellitus; HF, heart failure; HT, hypertension; SMI, severe mental illness.

For peer review only - http://bmjopen.bmj.com/site/about/guidelines.xhtml

Supplementary data to "Clinical code usage in UK general practice: a cohort study exploring 18 conditions over 14 years", Zghebi et al. 2021.

Figure S3 Jackknife correlation using 1-year window (incident codes) for 18 mental and physical conditions

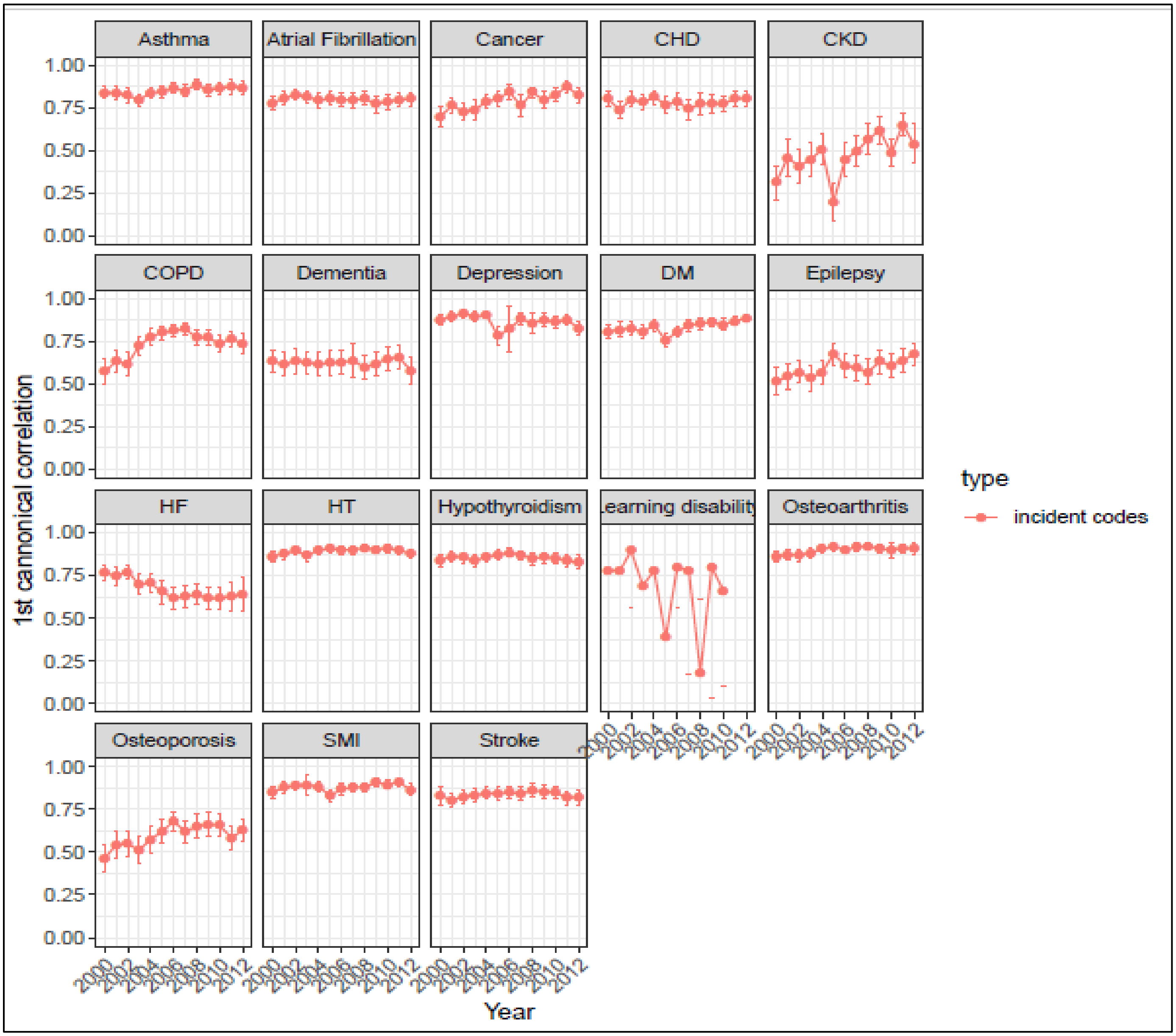

CHD, coronary heart disease; CKD, chronic kidney disease; COPD, chronic obstructive pulmonary disease; DM, diabetes mellitus; HF, heart failure; HT, hypertension; SMI, severe mental illness.

Incident code: a clinical code indicating new (incident) cases.

Supplementary data to "Clinical code usage in UK general practice: a cohort study exploring 18 conditions over 14 years", Zghebi et al. 2021.

Figure S4 Jackknife correlation using 5-year window (incident codes) for 18 mental and physical conditions

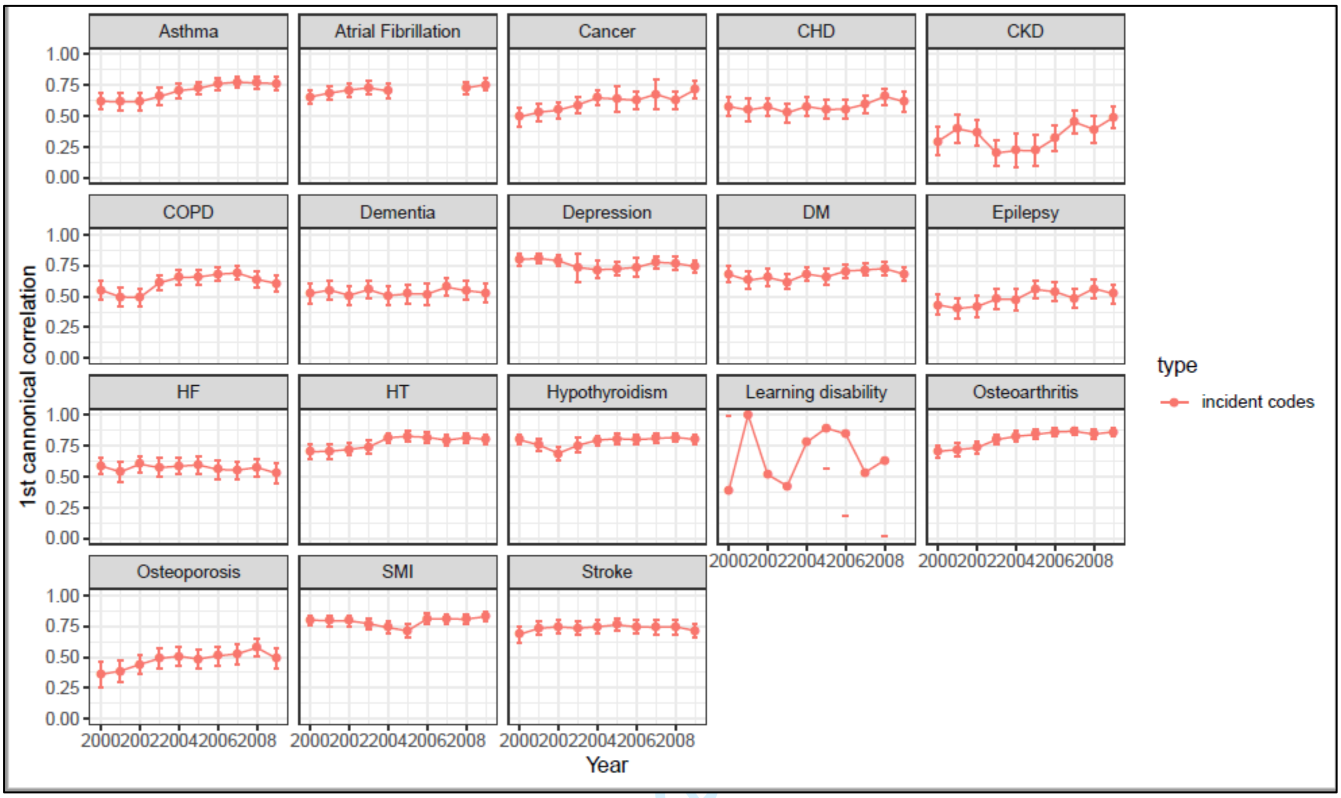

CHD, coronary heart disease; CKD, chronic kidney disease; COPD, chronic obstructive pulmonary disease; DM, diabetes mellitus; HF, heart failure; HT, hypertension; SMI, severe mental illness.

Incident code: a clinical code indicating new (incident) cases.

For peer review only - http://bmjopen.bmj.com/site/about/guidelines.xhtml

The RECORD statement – checklist of items, extended from the STROBE statement, that should be reported in observational studies using routinely collected health data.

	Item No.	STROBE items	Location in manuscript where items are reported	RECORD items	Location in manuscript where items are reported
**Title and abstract**					
	1	(a) Indicate the study's design with a commonly used term in the title or the abstract (b) Provide in the abstract an informative and balanced summary of what was done and what was found	**Title and Abstract**	RECORD 1.1: The type of data used should be specified in the title or abstract. When possible, the name of the databases used should be included.<br><br>RECORD 1.2: If applicable, the geographic region and timeframe within which the study took place should be reported in the title or abstract.<br><br>RECORD 1.3: If linkage between databases was conducted for the study, this should be clearly stated in the title or abstract.	**Abstract**<br><br>**Title and Abstract**<br><br>**Not applicable**
**Introduction**					
Background rationale	2	Explain the scientific background and rationale for the investigation being reported	**Introduction**		
Objectives	3	State specific objectives, including any prespecified hypotheses	**Abstract, Introduction**		
**Methods**					
Study Design	4	Present key elements of study design early in the paper	**Methods**		
Setting	5	Describe the setting, locations, and relevant dates, including	**Abstract, Methods**		

		periods of recruitment, exposure, follow-up, and data collection			
Participants	6	*(a)* ***Cohort study*** - Give the eligibility criteria, and the sources and methods of selection of participants. Describe methods of follow-up<br><br>*(b)* ***Cohort study*** - For matched studies, give matching criteria and number of exposed and unexposed<br>*Case-control study* - For matched studies, give matching criteria and the number of controls per case	**Methods**<br><br>**Not applicable**	RECORD 6.1: The methods of study population selection (such as codes or algorithms used to identify subjects) should be listed in detail. If this is not possible, an explanation should be provided.<br><br>RECORD 6.2: Any validation studies of the codes or algorithms used to select the population should be referenced. If validation was conducted for this study and not published elsewhere, detailed methods and results should be provided.<br><br>RECORD 6.3: If the study involved linkage of databases, consider use of a flow diagram or other graphical display to demonstrate the data linkage process, including the number of individuals with linked data at each stage.	**Methods**<br><br>**Not applicable**<br><br>**Not applicable**
Variables	7	Clearly define all outcomes, exposures, predictors, potential confounders, and effect modifiers. Give diagnostic criteria, if applicable.	**Methods**	RECORD 7.1: A complete list of codes and algorithms used to classify exposures, outcomes, confounders, and effect modifiers should be provided. If these cannot be reported, an explanation should be provided.	**Methods** (codes available from online repository: clinicalcodes.org)
Data sources/ measurement	8	For each variable of interest, give sources of data and details of methods of assessment (measurement).	**Methods**		

For peer review only

		Describe comparability of assessment methods if there is more than one group			
Bias	9	Describe any efforts to address potential sources of bias	**Methods**<br>Also, the used population (CPRD) is representative of the UK population		
Study size	10	Explain how the study size was arrived at	**Methods**		
Quantitative variables	11	Explain how quantitative variables were handled in the analyses. If applicable, describe which groupings were chosen, and why	**Methods**		
Statistical methods	12	(a) Describe all statistical methods, including those used to control for confounding<br>(b) Describe any methods used to examine subgroups and interactions<br>(c) Explain how missing data were addressed<br>(d) ***Cohort study*** - If applicable, explain how loss to follow-up was addressed<br>(e) Describe any sensitivity analyses	**Methods**		
Data access and cleaning methods		..		RECORD 12.1: Authors should describe the extent to which the investigators had access to the database population used to create the study population.<br><br>RECORD 12.2: Authors should provide information on the data cleaning methods used in the study.	**Methods**<br><br><br><br>**Not applicable**

For peer review only

Linkage		..		RECORD 12.3: State whether the study included person-level, institutional-level, or other data linkage across two or more databases. The methods of linkage and methods of linkage quality evaluation should be provided.	**Not applicable**
**Results**					
Participants	13	(a) Report the numbers of individuals at each stage of the study (*e.g.*, numbers potentially eligible, examined for eligibility, confirmed eligible, included in the study, completing follow-up, and analysed)<br>(b) Give reasons for non-participation at each stage.<br>(c) Consider use of a flow diagram	**Not applicable**	RECORD 13.1: Describe in detail the selection of the persons included in the study (*i.e.,* study population selection) including filtering based on data quality, data availability and linkage. The selection of included persons can be described in the text and/or by means of the study flow diagram.	**Methods, Results**
Descriptive data	14	(a) Give characteristics of study participants (*e.g.*, demographic, clinical, social) and information on exposures and potential confounders<br>(b) Indicate the number of participants with missing data for each variable of interest<br>(c) ***Cohort study*** - summarise follow-up time (*e.g.*, average and total amount)	**Not applicable**		
Outcome data	15	***Cohort study*** - Report numbers of outcome events or summary measures over time	**Results**		
Main results	16	(a) Give unadjusted estimates and, if applicable, confounder-adjusted estimates and their precision (e.g., 95% confidence	**Results, Figures 1-5, Figures S1 – S4.**		

For peer review only

		interval). Make clear which confounders were adjusted for and why they were included<br>(b) Report category boundaries when continuous variables were categorized<br>(c) If relevant, consider translating estimates of relative risk into absolute risk for a meaningful time period			
Other analyses	17	Report other analyses done—e.g., analyses of subgroups and interactions, and sensitivity analyses	**Not applicable**		
**Discussion**					
Key results	18	Summarise key results with reference to study objectives	**Discussion**		
Limitations	19	Discuss limitations of the study, taking into account sources of potential bias or imprecision. Discuss both direction and magnitude of any potential bias	**Discussion**	RECORD 19.1: Discuss the implications of using data that were not created or collected to answer the specific research question(s). Include discussion of misclassification bias, unmeasured confounding, missing data, and changing eligibility over time, as they pertain to the study being reported.	**Discussion / Conclusion**
Interpretation	20	Give a cautious overall interpretation of results considering objectives, limitations, multiplicity of analyses, results from similar studies, and other relevant evidence	**Discussion**		
Generalisability	21	Discuss the generalisability (external validity) of the study results	**Not applicable**		

For peer review only - http://bmjopen.bmj.com/site/about/guidelines.xhtml

**Other Information**					
Funding	22	Give the source of funding and the role of the funders for the present study and, if applicable, for the original study on which the present article is based	**Funding**		
Accessibility of protocol, raw data, and programming code		..		RECORD 22.1: Authors should provide information on how to access any supplemental information such as the study protocol, raw data, or programming code.	**Methods** (raw data can only by accessed via the CPRD)

*Reference: Benchimol EI, Smeeth L, Guttmann A, Harron K, Moher D, Petersen I, Sørensen HT, von Elm E, Langan SM, the RECORD Working Committee. The REporting of studies Conducted using Observational Routinely-collected health Data (RECORD) Statement. *PLoS Medicine* 2015; in press.

*Checklist is protected under Creative Commons Attribution (CC BY) license.

For peer review only

BMJ Open

Clinical code usage in UK general practice: a cohort study exploring 18 conditions over 14 years

Journal:	*BMJ Open*
Manuscript ID	bmjopen-2021-051456.R1
Article Type:	Original research
Date Submitted by the Author:	24-Oct-2021

Complete List of Authors:	Zghebi, Salwa; The University of Manchester, NIHR School for Primary Care Research, Centre for Primary Care and Health Services Research, Manchester Academic Health Science Centre (MAHSC); The University of Manchester, Division of Population Health, Health Services Research and Primary Care, School of Health Sciences, Faculty of Biology, Medicine and Health, Manchester Academic Health Science Centre (MAHSC)<br>Reeves, David; The University of Manchester, NIHR School for Primary Care Research, Centre for Primary Care and Health Services Research, Manchester Academic Health Science Centre (MAHSC); The University of Manchester, Centre for Biostatistics, School of Health Sciences, Faculty of Biology, Medicine and Health, Manchester Academic Health Science Centre (MAHSC)<br>Grigoroglou, Christos; The University of Manchester, Manchester Centre for Health Economics, Division of Population Health, Health Services Research and Primary Care, Manchester Academic Health Science Centre (MAHSC)<br>McMillan, Brian; The University of Manchester, NIHR School for Primary Care Research, Centre for Primary Care and Health Services Research, Manchester Academic Health Science Centre (MAHSC); The University of Manchester, Division of Population Health, Health Services Research and Primary Care, School of Health Sciences, Faculty of Biology, Medicine and Health, Manchester Academic Health Science Centre (MAHSC)<br>Ashcroft, Darren; The University of Manchester, NIHR School for Primary Care Research, Centre for Primary Care and Health Services Research, Manchester Academic Health Science Centre (MAHSC); The University of Manchester, Centre for Pharmacoepidemiology and Drug Safety, School of Health Sciences, Faculty of Biology, Medicine and Health, Manchester Academic Health Science Centre (MAHSC)<br>Parisi, Rosa; The University of Manchester, NIHR School for Primary Care Research, Centre for Primary Care and Health Services Research, Manchester Academic Health Science Centre (MAHSC); The University of Manchester, Division of Informatics, Imaging, and Data Sciences, School of Health Sciences, Faculty of Biology, Medicine and Health, Manchester Academic Health Science Centre (MAHSC)<br>Kontopantelis, Evangelos; The University of Manchester, NIHR School for Primary Care Research, Centre for Primary Care and Health Services Research, Manchester Academic Health Science Centre (MAHSC); The University of Manchester, Division of Informatics, Imaging, and Data Sciences, School of Health Sciences, Faculty of Biology, Medicine and Health, Manchester Academic Health Science Centre (MAHSC)

**Primary Subject Heading**:	General practice / Family practice
Secondary Subject Heading:	Health informatics, Public health
Keywords:	PRIMARY CARE, PUBLIC HEALTH, Change management < HEALTH SERVICES ADMINISTRATION & MANAGEMENT

SCHOLARONE™ Manuscripts

I, the Submitting Author has the right to grant and does grant on behalf of all authors of the Work (as defined in the below author licence), an exclusive licence and/or a non-exclusive licence for contributions from authors who are: i) UK Crown employees; ii) where BMJ has agreed a CC-BY licence shall apply, and/or iii) in accordance with the terms applicable for US Federal Government officers or employees acting as part of their official duties; on a worldwide, perpetual, irrevocable, royalty-free basis to BMJ Publishing Group Ltd ("BMJ") its licensees and where the relevant Journal is co-owned by BMJ to the co-owners of the Journal, to publish the Work in this journal and any other BMJ products and to exploit all rights, as set out in our licence.

The Submitting Author accepts and understands that any supply made under these terms is made by BMJ to the Submitting Author unless you are acting as an employee on behalf of your employer or a postgraduate student of an affiliated institution which is paying any applicable article publishing charge ("APC") for Open Access articles. Where the Submitting Author wishes to make the Work available on an Open Access basis (and intends to pay the relevant APC), the terms of reuse of such Open Access shall be governed by a Creative Commons licence – details of these licences and which Creative Commons licence will apply to this Work are set out in our licence referred to above.

Other than as permitted in any relevant BMJ Author's Self Archiving Policies, I confirm this Work has not been accepted for publication elsewhere, is not being considered for publication elsewhere and does not duplicate material already published. I confirm all authors consent to publication of this Work and authorise the granting of this licence.

For peer review only

Clinical code usage in UK general practice: a cohort study exploring 18 conditions over 14 years

Salwa S Zghebi,[1,2*] David Reeves,[1,2,3] Christos Grigoroglou,[4] Brian McMillan,[1,2] Darren M Ashcroft,[1,5] Rosa Parisi,[1,6] Evangelos Kontopantelis[1,2,6]

1 NIHR School for Primary Care Research, Centre for Primary Care and Health Services Research, Manchester Academic Health Science Centre (MAHSC), The University of Manchester, Manchester, UK.

2 Division of Population Health, Health Services Research and Primary Care, School of Health Sciences, Faculty of Biology, Medicine and Health, Manchester Academic Health Science Centre (MAHSC), The University of Manchester, Manchester, UK.

3 Centre for Biostatistics, School of Health Sciences, Faculty of Biology, Medicine and Health, Manchester Academic Health Science Centre (MAHSC), The University of Manchester, Manchester, UK.

4 Manchester Centre for Health Economics, Division of Population Health, Health Services Research and Primary Care, Manchester Academic Health Science Centre (MAHSC), The University of Manchester, Manchester, UK.

5 Centre for Pharmacoepidemiology and Drug Safety , School of Health Sciences, Faculty of Biology, Medicine and Health, Manchester Academic Health Science Centre (MAHSC), The University of Manchester, Manchester, UK.

6 Division of Informatics, Imaging, and Data Sciences, School of Health Sciences, Faculty of Biology, Medicine and Health, Manchester Academic Health Science Centre (MAHSC), The University of Manchester, Manchester, UK.

*Correspondence to:
Dr Salwa Zghebi, NIHR School for Primary Care Research, Centre for Primary Care and Health Services Research, Williamson Building, University of Manchester, Manchester M13 9PL, UK. E-mail: salwa.zghebi@manchester.ac.uk

Number of words (Abstract): 292
Number of words (Main text): 4,628
Number of Figures: 5

For peer review only

Abstract

Objectives

To assess the diagnostic Read code usage for 18 conditions by examining their frequency and diversity in UK primary care between 2000 and 2013.

Design

Population-based cohort study

Setting

684 UK general practices contributing data to the Clinical Practice Research Datalink (CPRD) GOLD.

Participants

Patients with clinical codes for at least one of: asthma, chronic obstructive pulmonary disease (COPD), diabetes, hypertension, coronary heart disease, atrial fibrillation, heart failure, stroke, hypothyroidism, chronic kidney disease, learning disability (LD), depression, dementia, epilepsy, severe mental illness (SMI), osteoarthritis, osteoporosis, and cancer.

Primary and secondary outcome measures

For the frequency ranking of clinical codes, canonical correlation analysis was applied to 1-, 3-, and 5-year correlations of clinical code usage. Three measures of diversity (Shannon entropy index of diversity, richness, and evenness) were used to quantify changes in incident and total clinical codes.

Results

Overall, all examined conditions except LD, showed positive monotonic correlation. Hypertension, hypothyroidism, osteoarthritis, and SMI codes' usage had high 5-year correlation. The codes' usage diversity remained stable overall throughout the study period. Cancer, diabetes, and SMI had the highest richness (code lists need time to define) unlike atrial fibrillation, hypothyroidism, and LD. SMI (high richness) and hypothyroidism (low richness) can last for 5 years, whereas, cancer and diabetes (high richness) and LD (low richness) only last for 2 years.

Conclusions

This is an underreported research area and the findings suggest the codes' usage diversity for most conditions remained overall stable throughout the study period. Generated mental health code lists can last for a long time unlike cardiometabolic conditions and cancer. Adopting more consistent and less diverse coding would help improve data quality in primary care. Future research is needed following the transfer to the Systematized Nomenclature of Medicine Clinical Terms (SNOMED CT) coding.

Keywords

Primary care, clinical codes, electronic health records, QOF, Quality and Outcomes Framework.

For peer review only - http://bmjopen.bmj.com/site/about/guidelines.xhtml

Strengths and limitations of this study

- Our study presents a contemporary longitudinal analysis of clinical code usage in UK primary care, addressing an underreported research area.
- Our findings are relevant to clinical practice as we examined 18 physical and mental conditions as recorded in primary care over 14 years, using data from a large nationally representative database.
- Given the design of the recorded electronic health records, we may have missed some patients with these 18 conditions (such as patients not registered with general practices) which may have affected the observed patterns of clinical code usage.
- Our analysis used CPRD GOLD data, which are obtained from clinical practices with the VISION clinical system, and EMIS and SystmOne practices will be using somewhat different diagnostic codes.

For peer review only

Introduction

The use of electronic health records (EHRs) has increased rapidly over the last three decades.[1] This has enabled researchers from various disciplines to examine cross-sectional and longitudinal trends of large population medical records to address many clinical research questions. EHRs are increasingly used for clinical management, clinical audits and research with real-world data, applying cross-sectional to longitudinal study designs to address descriptive epidemiology, pharmacoepidemiology, interventions evaluation, and risk prediction modelling.[2,3] The available routinely collected data are far from perfect, but they provide a wealth of high-quality information on patients' clinical conditions, referrals, and medication usage,[4] informing important components of clinical practice such as clinical decision-making.

Since the beginning of medical computing systems usage from early 1970s,[5,6] the UK's primary care systems became fully computerised by 2003.[7,8] This transition was facilitated by Read codes, a comprehensive computerised semi-hierarchical clinical classification system designed for use in EHRs, which are still in use in the UK.[9] These were originally developed by a clinician, Dr James Read, in the early 1980's and became the main coding system for clinical data in the UK from the mid-1990s, succeeding the Oxford Medical Information System (OXMIS) codes that were the most widely used system throughout the 1980s.[10-12] However, the Systematized Nomenclature of Medicine Clinical Terms (SNOMED CT), a systematically organised collection of medical terms, is being rolled out in general practices in a phased approach from April 2018 to replace Read codes, and it includes symptoms, diagnoses, procedures, family history, allergies, and devices.[13,14] With evident increasing complexity of most healthcare disciplines,[15] such clinical terminologies make collated patient records more manageable in clinical practice settings.[16,17] To support users, national standards and guidelines are available on the use of clinical coding.[14,18] Several UK primary care electronic databases exist and are managed by different and varying computer software systems (EMIS, Vision and SystmOne), with Read codes still being the most common system through which to capture primary care clinical information. In the UK, the largest primary care databases available for research purposes include the Clinical Practice Research Datalink (CPRD), The Health Improvement Network (THIN), ResearchOne, and QResearch.[2,8]

For peer review only - http://bmjopen.bmj.com/site/about/guidelines.xhtml

Despite the fact that clinical coding is a key point in the daily functionality of routine clinical practice, studies investigating their usage in real-world electronic databases are limited, although the observed variation in coding practice between clinicians.[19] The use of codes is a fundamental aspect of analyses of EHRs, involving a considerable amount of work, through which researchers extract a final dataset to analyse. Clinical codes are commonly used and disseminated in the form of code lists which are compiled according to the purpose, such as diagnostic codes, or family history codes (Table S1). Accurate (high specificity and sensitivity) code lists are imperative in obtaining reliable data on exposures, covariates, and outcomes. Previous systematic reviews have reported overall high accuracy of discharge coding (completed by clinical and/or administrative staff) in UK EHRs data that is improving over time, where in one review accuracy was defined as the agreement between the codes allocated after independently assessing clinical notes (acting as a 'gold' standard) and those recorded on EHRs.[20,21] However, clinical practice changes over time, at varying degrees for different conditions, which is reflected in coding practice with new codes being introduced and others made redundant.

Thus, examining and quantifying the changes in clinical code usage over time is important, since alterations in usage that have not been considered, can have important implications for the analysis of EHRs, resource allocation and may inform public health policy. An example for EHR analysis implication, the use of a two-year old code-list for a given medical condition may or may not be a problem, depending on how much clinical practice has changed over time for that condition. This change in clinical practice may be driven by policy changes, such as better reimbursement for keeping a register of certain conditions. A study examining the variation in clinical code use in UK primary care using six clinical terms, found that searches for the same clinical term across four different computer systems resulted in different results e.g. the mean number of codes per list ranging between 12.7-35.2 codes.[22] This highlighted the need for a more consistent system of code usage, with a recommendation to replace primary care code lists with shorter lists and fewer number of coding choices.[22] Importantly, the UK National Health Service (NHS) introduced the Quality and Outcomes Framework (QOF) in April 2004, a voluntary reward and incentive programme to reward UK general practices providing high-quality care based on a range of evidence-based clinical indicators, for example, management of common chronic conditions such as diabetes and asthma.[23,24,25] Furthermore,

For peer review only - http://bmjopen.bmj.com/site/about/guidelines.xhtml

Important revisions were introduced to QOF in April 2006 (covering up to March 2007)[26,27] including adding new indicators for diabetes, amending diabetes clinical indicator sets, and redefining the diabetes register so general practitioners required to identify patients with diabetes as either having type 1 or type 2 diabetes, which have potentially increased the capture of diabetes cases on that period. In this study, we used data from the UK CPRD GOLD database to examine the: 1) frequency ranking of diagnostic clinical codes for 18 physical and mental health conditions; 2) changes in the usage of individual clinical codes (incident vs. total codes) for these conditions between 2000-2013 covering the period before and after the launch of the QOF.

Methods

Data source and study design

We used data from the GOLD database of the UK Clinical Practice Research Datalink (CPRD), which comprises of data from contributing anonymised general practices using the VISION clinical computer system.[28] The CPRD is one of the world's largest longitudinal electronic medical databases providing anonymised data from primary care, and is broadly representative of the UK population.[8,29] The CPRD is structured to provide data on clinical information, referrals, consultations, immunisation, tests and prescribed therapies. Up to July 2013, the CPRD held data for 11.3 million patients registered in 674 general practices. Of these, 4.4 million were active patients (representing 6.9% of the total UK population), and 6.9 million records represent inactive patients (people who have died or are no longer registered with a participating general practice).[29]

Using financial year intervals between 01/04/2000-31/03/2013, we examined the changes in the use of diagnostic clinical codes for 18 exemplar medical conditions in UK practices: asthma, chronic obstructive pulmonary disease (COPD), diabetes (DM) both types, hypertension (HT), coronary heart disease (CHD), atrial fibrillation (AF), heart failure (HF), stroke, hypothyroidism, chronic kidney disease (CKD), learning disability (LD), depression, dementia, epilepsy, severe mental illness (SMI), osteoarthritis, osteoporosis, and cancer. The diabetes codes included those with complications if clearly linked to diabetes, such as 'type 2

For peer review only - http://bmjopen.bmj.com/site/about/guidelines.xhtml

diabetes mellitus with nephropathy' (Table S1). The selected conditions, apart from osteoarthritis, were included in the QOF scheme from 2004, whereas AF, CKD, dementia, depression, and LD were incentivised from 2006, and osteoporosis incentivised from 2012. This allowed us to examine and compare QOF conditions (incentivised at different stages) plus a condition not part of the QOF (osteoarthritis).

The clinical codes used to define the examined conditions are listed in the Clinical Codes online repository.[30] Each condition was examined as an incident code (using codes to identify new cases) and total codes (incident and prevalent cases) for each year during the study period.

Data analysis

To examine the consistency of clinical code use across time, we applied canonical correlation analysis (CAA)[31,32] to estimate 1-year (e.g. 2006 to 2007), 3-year (e.g. 2006 to 2009), and 5-yearly canonical correlations (e.g. 2006 to 2011) for code usage for each of the 18 conditions based on ranking the percentage frequency use of codes. CCA is a descriptive multivariable method that provides a measure of the canonical correlation (CC) between two groups of variables or two data matrices that should be numerically complete and non-missing. CCA finds the best linear combinations maximizing the correlation (γ_1) between p variables in group 1 and q variables in group 2, where the variables are measured across a common set of units (e.g. general practices): [33]

$$Y^1 = (Y_1^1, \cdots, Y_p^1) \quad (1)$$

$$Y^2 = (Y_1^2, \cdots, Y_q^2) \quad (2)$$

Where Y^1 represents the set of p outcomes in group 1, and Y^2 the set of q outcomes in group 2. Consider the two linear combinations $\alpha' Y^1$ and $b' Y^2$, where α' is a $p \; x \; 1$ vector of weighting coefficients and b' is likewise a $q \; x \; 1$ vector; the CC (γ_1) is given by the choice of α' and b' that maximises the correlation between $\alpha' Y^1$ and $b' Y^2$: [33]

$$\gamma_1 = \max_{a,b} Corr(a' Y^1, b' Y^2) = \max_{a,b} \frac{a' \Sigma_{12} b}{\sqrt{a' \Sigma_{11} a b' \Sigma_{22} b}} \quad (3)$$

Where Σ_{11} = $Cov(Y^{(1)}, Y^{(1)})$; Σ_{12} = $Cov(Y^{(1)}, Y^{(2)})$; and Σ_{22} = $Cov(Y^{(2)}, Y^{(2)})$. (4)

For peer review only - http://bmjopen.bmj.com/site/about/guidelines.xhtml

In the present study, for a given practice the Y's represent the relative use of each clinical code for a particular condition, expressed as a percentage of the total use across all codes for that condition. For example, for the 2006-2007 year-on-year diabetes correlation, the Y's represents the relative use of each diabetes code expressed as a percentage of the total use across all diabetes codes, where group 1 (represented by Y^1) would be the percentage frequency use of each clinical code for diabetes recorded in year 2006; whereas group 2 (represented by Y^2) would be the corresponding percentage frequency use for each corresponding diabetes code recorded in year 2007, at the general practice level. The same applies for the 3-year and 5-year correlations.

We analysed percentage frequencies rather than frequency counts so as to remove any effects of variations in practice size or disease prevalence from the estimated CCs. CCs were calculated using the R statistical software ccaPP package[34] with the "Spearman" method, by which the weighted linear combinations $\alpha' Y^1$ and $b' Y^2$ for each year are ranked across practices prior to computation of the correlation. This method produces estimates that are more robust against model misspecification.[35]

Numbers of incident clinical codes could be small for some conditions and practices, which can lead to biased estimates of the CCs. To adjust for this, we applied the Jackknife bias correction to the estimation of CCs for the incidence of clinical codes.[33]

For each of the 18 conditions, we also quantified changes in incident and total clinical code usage applying three measures of diversity. First, the Shannon entropy (H), an equitability and popular index of diversity. The index is interchangeably referred to as Shannon entropy or Shannon index where the term 'entropy' indicates the uncertainty or variability of information in a variable whose diversity is assessed by the Shannon index. The Shannon entropy index (H) was calculated as:

$$H = -\sum_i (p_i \ln p_i)$$

Where p_i is the proportion of a clinical code *i* usage in a given year.

Second, we examined the richness (S) of clinical code usage by calculating the annual total number of incident and all codes used in a given year. Third, we estimated the evenness (J)

For peer review only - http://bmjopen.bmj.com/site/about/guidelines.xhtml

of incident and total codes' usage, a measure of the relative usage of codes within a given year, in other words evenness will be high if all codes have a similar distribution (e.g. 100 diabetes records based on using 4 different diabetes codes, 25 times each) whereas it will be low if a few codes dominate the code usage (e.g. 100 diabetes records based on using one code 70 times and another code 30 times). J ranges between zero and one, with $J = 0$ indicating no evenness, and $J = 1$ indicating complete evenness. Evenness was calculated annually by dividing Shannon index (H) over the natural logarithm of richness (S).

$$J = \frac{H}{\ln(S)}$$

To simplify what these diversity measures imply, we describe a hypothetical example: if diabetes was represented using three diagnostic codes: code A (used 100 times), code B (used 175 times), and code C (used 350 times), then the proportions of codes would be 0.16, 0.28, and 0.56, respectively. Shannon's entropy index (H) will be = -1*((0.16*In0.16) + (0.28*In0.28) + (0.56*In0.56)) = 0.97; richness (S) = 3; and evenness (J) = 0.97/ln (3) = 0.88. All analyses were conducted using R software,[36] and were visualised using the ggplots2 package. A copy of the R code is presented in Table S2.

Patient and Public Involvement

No patients or members of the public were involved in this study.

Results

Clinical code frequency ranking

Correlation of code usage over a 3-year period showed a positive association for most conditions (Figure 1). Strong, overall positive, and monotonic correlation (CC >0.7) was observed for depression, hypertension, hypothyroidism, osteoarthritis, SMI, and stroke. Positive, monotonic but weaker associations were observed for CKD, epilepsy, and osteoporosis. Learning disability (LD) showed a non-monotonic function with fluctuations ranging between 0.4-1.0, and a notable decline after 2004 before increasing again from 2007.

For peer review only - http://bmjopen.bmj.com/site/about/guidelines.xhtml

For peer review only

The 1-year and 5-year windows correlations showed similar overall trends, but the association was slightly decreasing as the window increases. Clinical conditions with the highest correlation levels were asthma, AF, cancer, CHD, depression, diabetes, hypertension, hypothyroidism, osteoarthritis, SMI, and stroke for the 1-year window (Figure S1). For the 5-year window, hypertension, hypothyroidism, osteoarthritis, and SMI codes' usage was overall highly correlated mainly in recent years (Figure S2). On the other hand, conditions with the lowest correlations (CC ≤0.6) were CKD and LD (for most years) for the 1-year window; and cancer, CHD, CKD, COPD, dementia, diabetes, epilepsy, HF, LD, and osteoporosis for the 5-year window.

Over a 3-year window, strong correlation for incident code usage (Jackknife bias corrected CC≥0.6) were observed for all examined conditions except CKD, epilepsy, and osteoporosis (Figure 2). Similarly, the 1-year and 5-year windows correlations showed similar trends but lower coefficients with longer windows (Figures S3 and S4, respectively).

Clinical code usage diversity

Data from 684 UK general practices contributing to the Clinical Practice Research Datalink (CPRD) GOLD were used. Overall, the diversity indices of code usage were stable over the study period for most conditions, but with wide confidence intervals. Higher entropy (H) indices were observed with cancer, diabetes, and SMI (H between 2-4), while the lowest levels were observed with LD and osteoporosis (H between 0-2) (Figure 3). Over time, the entropy index of code usage remained stable for most conditions, but increased gradually for asthma, COPD, diabetes, heart failure, and osteoporosis (primarily incident codes). Fluctuations and/or a separation between the incident and total codes trends were observed around 2006, mainly for AF, dementia, depression, CKD, and LD. The Shannon index (H) for incident codes had a similar trend to that for total codes for most conditions over time, except for cardiovascular disease (CVD) and diabetes where it exceeded total codes.

Across the examined conditions, the richness (S) of incident and total code usage (number of codes used) was the highest for cancer (>500 codes), diabetes and SMI (≥250 codes each) and the lowest for AF, hypothyroidism, and LD (S <100) (Figure 4). The trends however remained stable throughout the study period, except a small decrease for SMI codes and a decrease in

For peer review only - http://bmjopen.bmj.com/site/about/guidelines.xhtml

cancer after a brief rise between 2000-2005. The difference between the number of incident and total codes for SMI, diabetes and cancer were evident (total codes more than incident codes), unlike in the other conditions where the S index was similar for both code categories.

The evenness (J) of both incident and total codes was overall stable and almost identical at least up to 2006, before total codes surpassed incident codes for most conditions except for depression and dementia where the J index for incident codes exceeded that of total codes (Figure 5). The two exceptions to this observation were LD and CKD. For LD, evenness was stable ~0.75 between 2000-2003, declined in 2004 before re-increasing from 2007 and returning to pre-2004 levels from 2011 onwards. For CKD, evenness dipped briefly around 2006-2007 and started to increase again from 2008 until the end of the study period (2013). Given the calculation formula, it is worth noting that the trends of entropy were similar to that of evenness for conditions with low richness, namely for AF, dementia, heart failure, hypertension, hypothyroidism, LD, osteoarthritis, and osteoporosis.

Discussion

Main findings

We assessed the clinical code usage for 18 conditions recorded in a large nationally representative UK EHR between 2000 and 2013. The results show overall strong positive monotonic correlation for all examined conditions except LD, that showed a fluctuating pattern during the study period. The CCs diminished over longer windows (5-year vs. 1-year window). Hypertension, hypothyroidism, osteoarthritis, and SMI had the highest 5-year correlation, mainly in later years of the study period.

The codes' usage entropy and evenness diversity measures remained overall stable throughout the study period for most conditions, except gradual increases over time for respiratory conditions, diabetes, HF, and osteoporosis. This increase in diversity may be partially due to the regular addition of new diagnostic codes and domains over time. For example, major revisions were introduced to the QOF in April 2006 resulting in the addition of new clinical areas and indicators.[27] As a consequence, CKD is among the conditions that it has been acknowledged to have benefited from these revisions as the CKD domain was added in 2006 reflected in improved recording in primary care from that year onwards.[37] For most

For peer review only - http://bmjopen.bmj.com/site/about/guidelines.xhtml

conditions (except LD), evenness (indicating the abundance of codes in a sample) was overall ≥0.5 suggesting a uniform distribution of the codes. Cancer, diabetes, and SMI had the highest richness indices among all examined conditions.

Comparison with previous studies

Observational studies examining variations in clinical code usage are limited. A recent study examined the code usage of CVD between 2001-2015 in primary and secondary care records in England.[38] The study aimed to examine if temporal variability methods can identify changes in CVD recording by quantifying the differences of monthly distributions of the variables of interest: CVD status and sociodemographic variables. The study found variability in the frequency of CVD codes across time, potentially due to non-medical causes such as changes in coding used and coding guidelines e.g. changes in ICD (international classification of disease) coding in hospital records. Despite relevance, their approach (examining the prevalence of CVD stratified by patient demographic variables) differs from our methods and hence the results are not directly comparable. In addition, we examined code usage for 18 conditions, including CVD, from UK general practices.

A study by Tai et al. (2007) examined the diversity of data entry screens in four clinical computer systems available in UK general practices and assessed its impact on the variation and quality of recorded clinical data for six exemplar conditions (sore throat, tired all the time, depression, cystitis, type 2 diabetes, and myocardial infarction).[22] In agreement with what we report on the large number of available codes for some conditions (high richness), Tai and colleagues found that searches for the same clinical term across the systems resulted in different results and found long code lists where the mean number of codes ranged between 12.7 and 35.2 codes per list.[22] Their study concluded that the systems may contribute towards a diverse coding in primary care, suggesting the need to standardise clinical coding across systems and to adopt shorter and more restricted code lists to help improve data quality. This is an important issue for UK primary care, since the semi-structured and dynamic nature of Read codes often results in diverse and long clinical code lists. Their findings highlight the need for analyses as ours, which are lacking in current literature, investigating the real-world abundance and trends of code usage over time derived from routine clinical data. Additionally, some general practice systems use CTV3 clinical codes and not Read codes

For peer review only - http://bmjopen.bmj.com/site/about/guidelines.xhtml

resulting in the availability of two versions of clinical codes. SNOMED CT system, which is gradually being implemented across UK primary care from 2018, aimed to provide a single clinical terminology for effective and consistent exchange of clinical data across all NHS settings to help improve patient care and data analysis.[39] Being an international clinical terminology, SNOMED will allow the UK to participate in global health care research.

Our results showed that diabetes codes usage (types 1 and 2) had one of the highest richness index levels (number of codes used), while the diversity entropy index was steadily increasing over the study period, highlighting the increasing variety of diabetes codes used in primary care over time. This observation agrees with a previous study that examined the Read codes used to identify diabetes management in people with diabetes registered with 17 general practices in one locality in London.[11] That study concluded that a wide range of diabetes codes were used and that the number of people assigned each code differed across practices. This again indicates that an approach is required to standardise clinical code lists and thereby coding usage as much as possible, minimise clinical recording errors, and improve research robustness.

Implication of findings

Our findings shed additional light on the use of clinical codes in research. We found that hypertension, hypothyroidism, osteoarthritis, and SMI codes' usage are highly correlated over the 5-year window (i.e. the codes' usage was similar across years), whereas cancer, CHD, CKD, COPD, dementia, diabetes, epilepsy, HF, LD, and osteoporosis had lowest correlation over the same window. In terms of clinical code lists' size required to define a condition (richness), we found that conditions with the highest richness across the study period were cancer, diabetes, and SMI (between 250-875 codes), whereas AF, hypothyroidism, and LD had the lowest richness (<100 codes). Collectively, these findings indicate that diabetes, cancer, and SMI codes have high richness and need to be defined carefully and then they can either last for 5 years (SMI), or only to 2 years (diabetes and cancer). Whereas hypothyroidism has low code usage richness and can last for 5 years. This might be due to that diabetes is often a target of government initiatives, unlike hypothyroidism which is rarely a focus of such interventions.

For peer review only - http://bmjopen.bmj.com/site/about/guidelines.xhtml

The results suggest that defining cohorts of people with mental health conditions (SMI, depression) over time was less sensitive to the changes of code usage (up to 5 years old) compared with most cardiometabolic conditions and cancer.

The observed findings also suggest the need to adopt a more consistent and less diverse coding in primary care, as this will help improve data quality. Inconsistent use of clinical coding may result in people with the same condition not being flagged as having the condition,[19] which may have implications on searching and identifying these people for clinical and research purposes, or to identify people for shielding measures or those who are a priority for a vaccination as in the current COVID-19 pandemic. While acknowledging that SNOMED CT is gradually replacing Read codes in general practice care since April 2018, our findings are still relevant in documenting the clinical code usage over a long period where Read codes were the main UK coding system. There is potential for SNOMED CT terminology to improve coding consistency, mainly through the plan to implement it in both primary care and secondary care systems.[40] However, a possible issue with SNOMED terminology is the need for specialist browser and reference sets, such as the general practice reference set to handle the long hierarchies of SNOMED system.[40] A reference set is a mechanism that can be employed to represent value sets of SNOMED CT components.[41]

Also, the rapidly increasing complexity of healthcare systems[15] might play a role on the observed trends in code usage over time. In other words, code usage practices (e.g. the tendency of data enterer to use easily accessed and well-known codes) may be partially driven by personal and work factors in the complex healthcare systems such as limited time and organisational factors. A possible relationship between clinical code selection and epidemiology of chronic conditions has been reported previously, for example, for diabetes.[19,42] From general practitioners (GPs) stance, one thing that may have changed in recent years is the coding of people being at "High risk of diabetes mellitus" and it is something that GPs are increasingly aware of (i.e. people with HbA_{1c} 42-47 mmol/mol).

Strengths and limitations of the study

Our study has several strengths. Using a range of frequency and diversity measures, we present a contemporary longitudinal analysis of clinical code usage in UK primary care, while only a few existing studies have addressed this research area. We used data from a large nationally representative database, where the validity of recorded diagnostic coding has been

For peer review only - http://bmjopen.bmj.com/site/about/guidelines.xhtml

acknowledged previously.[43] Additionally, the data quality is assumed to be high, as it is based on QOF clinical codes lists (except osteoarthritis). Our findings are relevant to clinical practice as we examined a broad range of prevalent physical and mental illnesses as recorded in primary care and considered the clinical implications of variations in clinical coding over 14 years.

Our study has also several limitations. Given the design of the recorded EHRs, we may have missed some patients with the examined conditions due to some unusual circumstances or settings, such as patients not registered with general practices (e.g. homeless people), which may have affected the observed patterns of clinical code usage. Also, analyses were not extended to examine ICD-10 clinical codes in secondary care setting (only available in England), as our aim was to focus on the usage of Read codes recorded in UK primary care visits as the main point of clinical care. CCA provides a single multivariate measure of correlation, thus simplifying interpretation compared to analysing each clinical code separately. However, the measure represents the maximum possible correlation between frequencies of code use at two different time points and does not account for the code set being the same at both times, hence may over-represent actual agreement to a degree. This is an intrinsic limitation of CCA is that it does not consider the 'code' per se but its frequency, so it would return high correlation for possible scenarios such as if a code merges at a time point with another code into a single code, or if, hypothetically, all practices transition from one code to another at the same time. As CCA is based on the correlation of two positive-definite matrices of data that should be numerically complete, problematic quality and levels of recording as observed with AF and LD recording on some time points results in missing values as observed in e.g. Figures 1, 2 and S4. In addition, although the clinical relevance of the examined conditions which are also prevalent outside UK, such as diabetes, CVD, and cancer, and that our findings highlight the need of consistent coding lists applies for non-UK national health system with established or aiming to develop electronic clinical coding, our study may have limited generalisability to non-UK systems. Finally, we used CPRD GOLD which collects data from general practices using the VISION clinical system, and code usage will vary to some extent in general practices using EMIS or SystmOne. However, we would expect such variation to be low in chronic conditions incentivised through the QOF, with specific common code lists used by practices to ensure remuneration eligibility.

For peer review only - http://bmjopen.bmj.com/site/about/guidelines.xhtml

Conclusions

The code usage in UK primary care was overall stable for most of the examined chronic conditions managed in general practice between 2000 and 2013, but, as would be expected, the changes were higher over longer time windows. Diabetes, cancer, and SMI code lists have high richness and need to be defined carefully by researchers and/or clinicians which might be considerably time-consuming, but once defined SMI codes can last up to 5 years, while diabetes and cancer codes only for 2 years. On the other hand, hypothyroidism has low richness but also can last up for 5 years. Our study addresses an underreported research area and the findings suggest the need to adopt a more consistent and less diverse coding in primary care to help improve data quality and the use of recent codes for cardiometabolic conditions and cancer. More research is needed in this area following the full transfer to the SNOMED CT coding and to examine the code usage in secondary care settings.

Acknowledgments

The authors would like to thank Dr David A. Springate (DAS) for extracting and analysing the data.

Funding

This study is funded by the National Institute for Health Research (NIHR) School for Primary Care Research (grant number 211). The views expressed are those of the authors and not necessarily those of the NIHR or the Department of Health and Social Care. The funder of the study had no role in study design, data collection, data analysis, data interpretation, or writing of the report. The lead author had full access to the data in the study, takes responsibility for its integrity and the data analysis, and had final responsibility for the decision to submit for publication.

Competing interests

DMA reports research grants from Abbvie, Almirall, Celgene, Eli Lilly, Novartis, UCB and the Leo Foundation. Other co-authors declare no competing interests.

For peer review only

For peer review only - http://bmjopen.bmj.com/site/about/guidelines.xhtml

Author contribution

EK and DAS designed the study. DAS extracted the data from all sources and performed the initial analyses. RP and SSZ validated the analyses; RP developed the final figures. SSZ wrote the manuscript and EK, RP, DR, and CG critically edited the initial drafts; DMA and BM contributed to interpretation of data and revised the paper for important intellectual content. All authors agreed on the final version of the paper before submission. SSZ is the guarantor of this work and, as such, had full access to all the data in the study and takes responsibility for the integrity of the data and the accuracy of the data analysis.

Patient consent

Not applicable.

Data sharing

Clinical code lists are available from clinicalcodes.org. Electronic health records are, by definition, considered sensitive data in the UK by the Data Protection Act and cannot be shared via public deposition because of information governance restriction in place to protect patient confidentiality. Access to data is available only once approval has been obtained through the individual constituent entities controlling access to the data. The data can be requested via application to the Clinical Practice Research Datalink.

Transparency declaration

SSZ affirms that the manuscript is an honest, accurate, and transparent account of the study being reported; that no important aspects of the study have been omitted; and that any discrepancies from the study as planned (and, if relevant, registered) have been explained.

Ethical approval

This study is based on data from Clinical Practice Research Datalink (CPRD) obtained under licence from the UK Medicines and Healthcare products Regulatory Agency. The study was approved by the Independent Scientific Advisory Committee (ISAC) for MHRA Database Research (protocol number: 16_115). The data are provided by patients and collected by the NHS as part of their care and support. Generic ethical approval for observational research

For peer review only - http://bmjopen.bmj.com/site/about/guidelines.xhtml

using CPRD with approval from ISAC has been granted by a Health Research Authority (HRA) Research Ethics Committee (East Midlands—Derby, REC reference number 05/MRE04/87).

Exclusive licence

The Corresponding Author has the right to grant on behalf of all authors and does grant on behalf of all authors, a worldwide licence (http://www.bmj.com/sites/default/files/BMJ%20Author%20Licence%20March%202013.doc) to the Publishers and its licensees in perpetuity, in all forms, formats and media (whether known now or created in the future).

For peer review only

For peer review only - http://bmjopen.bmj.com/site/about/guidelines.xhtml

References

1. Shephard E, Stapley S, Hamilton W. The use of electronic databases in primary care research. Family Practice 2011;**28**:352-54 doi: doi:10.1093/fampra/cmr039.
2. Casey JA, Schwartz BS, Stewart WF, et al. Using Electronic Health Records for Population Health Research: A Review of Methods and Applications. Annu Rev Public Health 2016;**37**:61–81 doi: 10.1146/annurev-publhealth-032315-021353.
3. Hippisley-Cox J, Coupland C, Brindle P. Development and validation of QRISK3 risk prediction algorithms to estimate future risk of cardiovascular disease: prospective cohort study. BMJ 2017;**357**:j2099 doi: doi: https://doi.org/10.1136/bmj.j2099
4. West-Strum D. Introduction to Pharmacoepidemiology. In: Yang Y, West-Strum D, eds. Understanding Pharmacoepidemiology. New York: McGraw Hill, 2011:7.
5. McMillan B ER, Brown B, Fitton R, Dickinson D. Primary Care Patient Records in the United Kingdom: Past, Present, and Future Research Priorities. J Med Internet Res 2018;**20**(12):e11293 doi: 10.2196/11293.
6. Benson T. Why general practitioners use computers and hospital doctors do not—Part 1: incentives. BMJ 2002;**325** (7372):1086–9 doi: 10.1136/bmj.325.7372.1086.
7. Millman A, Lee N, Brooke A. ABC of Medical Computing: Computers in general practice--I. BMJ 1995;**311**(800) doi: doi: https://doi.org/10.1136/bmj.311.7008.800
8. Kontopantelis E, Stevens R, Helms P, et al. Spatial distribution of clinical computer systems in primary care in England in 2016 and implications for primary care electronic medical record databases: a cross sectional population study. BMJ Open 2018;**8**:e020738 doi: 10.1136/bmjopen-2017-020738.
9. NHS Digital. Read Coded Clinical Terms. Secondary Read Coded Clinical Terms. 30 May 2019. https://www.datadictionary.nhs.uk/web_site_content/supporting_information/clinical_coding/read_coded_clinical_terms.asp?shownav=1.
10. Booth N. What are the Read Codes? Health Libraries Review 1994;**11**(3):177-82.
11. Gray J, Orr D, Majeed A. Use of Read codes in diabetes management in a south London primary care group: implications for establishing disease registers. BMJ 2003;**326**(1130) doi: https://doi.org/10.1136/bmj.326.7399.1130.
12. Benson T. The history of the Read codes: the inaugural James Read Memorial Lecture 2011. Informatics in Primary Care 2011;**19**:173–82.
13. NHS Digital. SNOMED CT. Secondary SNOMED CT 17 January 2020 2020. https://digital.nhs.uk/services/terminology-and-classifications/snomed-ct.
14. NHS Digital. Terminology and Classifications. Secondary Terminology and Classifications. 23 May 2019. https://digital.nhs.uk/services/terminology-and-classifications.
15. Plsek PE, Greenhalgh T. The challenge of complexity in health care. BMJ 2001;**323**(625).
16. Williams R, Brown B, Kontopantelis E, et al. Term sets: A transparent and reproducible representation of clinical code sets. Plos One 2019;**14**(2):e0212291 doi: https://doi.org/10.1371/journal.pone.0212291.
17. Watson N. Using clinical coding systems to best effect in electronic records. Secondary Using clinical coding systems to best effect in electronic records. 2001.

For peer review only - http://bmjopen.bmj.com/site/about/guidelines.xhtml

https://www.guidelinesinpractice.co.uk/using-clinical-coding-systems-to-best-effect-in-electronic-records/305085.article.

18. The Joint Computing Group of the General Practitioners Committee and the Royal College of General Practitioners. Good practice guidelines for general practice electronic patient records (version 3) - Guidance for GPs, 2003.
19. Tate AR, Dungey S, Glew S, et al. Quality of recording of diabetes in the UK: how does the GP's method of coding clinical data affect incidence estimates? Cross-sectional study using the CPRD database. BMJ Open 2017;**7**:e012905 doi: doi: 10.1136/bmjopen-2016-012905.
20. Burns EM, Rigby E, Mamidanna R, et al. Systematic review of discharge coding accuracy. Journal of Public Health 2012;**34**(1):138–48.
21. Campbell SE, Campbell MK, Grimshaw JM, et al. Systematic review of discharge coding accuracy. Journal of Public Health 2001;**23**(3):205–11.
22. Tai TW, Anandarajah S, Dhoul N, et al. Variation in clinical coding lists in UK general practice: a barrier to consistent data entry? Informatics in Primary Care 2007;**15**:143–50.
23. Lester H, Campbell S. Developing Quality and Outcomes Framework (QOF) indicators and the concept of 'QOFability'. Quality in Primary Care 2010;**18**:103-9.
24. NHS Digital. Quality and Outcome Framework business rules. Secondary Quality and Outcome Framework business rules 31 May 2019. https://digital.nhs.uk/data-and-information/data-collections-and-data-sets/data-collections/quality-and-outcomes-framework-qof.
25. Campbell SM, Reeves D, Kontopantelis E, et al. Effects of Pay for Performance on the Quality of Primary Care in England. NEJM 2009;**361**:368-78 doi: DOI: 10.1056/NEJMsa0807651
26. Health and Social Care Information Centre. National Quality and Outcomes Framework Statistics for England 2006/07. Secondary National Quality and Outcomes Framework Statistics for England 2006/07 2007. https://files.digital.nhs.uk/publicationimport/pub05xxx/pub05997/qof-eng-06-07-bull-rep.pdf.
27. Calvert M, Shankar A, McManus RJ, et al. Effect of the quality and outcomes framework on diabetes care in the United Kingdom: retrospective cohort study. BMJ 2009;**338**:b1870.
28. Medicines & Healthcare products Regulatory Agency (MHRA). Clinical Practice Research Datalink. Secondary Clinical Practice Research Datalink 2021. https://www.cprd.com/.
29. Herrett E, Gallagher AM, Bhaskaran K, et al. Data Resource Profile: Clinical Practice Research Datalink (CPRD). International Journal of Epidemiology 2015;**44**(3):827-36 doi: 10.1093/ije/dyv098.
30. Springate DA, Kontopantelis E, Ashcroft DM, et al. ClinicalCodes: An Online Clinical Codes Repository to Improve the Validity and Reproducibility of Research Using Electronic Medical Records. PLoS ONE 2014;**9**(6):e99825 doi: DOI: 10.1371/journal.pone.0099825.
31. Yang X, Liu W, Liu W, et al. A Survey on Canonical Correlation Analysis. IEEE Transactions on Knowledge and Data Engineering 2021;**33**(6):2349-68 doi: 10.1109/TKDE.2019.2958342.
32. Canonical Correlation Analysis. Applied Multivariate Statistical Analysis. Berlin, Heidelberg: Springer, Berlin, Heidelberg, 2007:321-30.
33. Lee H-S. Canonical Correlation Analysis Using Small Number of Samples. Communications in Statistics - Simulation and Computation 2007;**36**(5):973-85 doi: 10.1080/03610910701539443.
34. Alfons A, Croux C, Filzmoser P. Robust maximum association between data sets: The R Package ccaPP. Austrian Journal of Statistics 2016;**45**(1):71–79 doi: https://doi.org/10.17713/ajs.v45i1.90.

For peer review only

For peer review only - http://bmjopen.bmj.com/site/about/guidelines.xhtml

35. Alfons A, Croux C, Filzmoser P. Robust Maximum Association Estimators. 2017;**112**(515):436-45 doi: https://doi.org/10.1080/01621459.2016.1148609.
36. R Core Team. R: A language and environment for statistical computing. R Foundation for Statistical Computing. Secondary R: A language and environment for statistical computing. R Foundation for Statistical Computing 2017. https://www.r-project.org/.
37. Nihat A, de Lusignan S, Thomas N, et al. What drives quality improvement in chronic kidney disease (CKD) in primary care: process evaluation of the Quality Improvement in Chronic Kidney Disease (QICKD) trial. BMJ Open 2016;**6**(e008480) doi: 10.1136/bmjopen-2015-008480.
38. Rockenschaub P, Nguyen V, Aldridge RW, et al. Data-driven discovery of changes in clinical code usage over time: a case-study on changes in cardiovascular disease recording in two English electronic health records databases (2001–2015). BMJ Open 2020;**10**(e034396) doi: doi:10.1136/bmjopen-2019-034396.
39. NHS Digital. SNOMED CT implementation in primary care. Secondary SNOMED CT implementation in primary care 21/03/2019 2019. https://digital.nhs.uk/services/terminology-and-classifications/snomed-ct/snomed-ct-implementation-in-primary-care.
40. Meek T. SNOMED to replace Read Codes by 2020. Secondary SNOMED to replace Read Codes by 2020 2015.
41. SNOMED International. Reference Set. Secondary Reference Set 2020. https://confluence.ihtsdotools.org/display/DOCRFSPG/2.3.+Reference+Set.
42. Zghebi SS, Steinke DT, Carr MJ, et al. Examining Trends in Type 2 Diabetes Incidence, Prevalence and Mortality in the UK between 2004 and 2014. Diab, Obes, and Metab 2017 doi: 10.1111/dom.12964.
43. Khan NF, Harrison SE, Rose PW. Validity of diagnostic coding within the General Practice Research Database: a systematic review. British Journal of General Practice 2010;**60**(572):e128-36 doi: 10.3399/bjgp10X483562.

For peer review only

For peer review only - http://bmjopen.bmj.com/site/about/guidelines.xhtml

Figure legends

Figure 1 Canonical correlations using 3-year window of clinical code usage for 18 mental and physical conditions

CHD, coronary heart disease; CKD, chronic kidney disease; COPD, chronic obstructive pulmonary disease; DM, diabetes mellitus; HF, heart failure; HT, hypertension; SMI, severe mental illness. The red line represents the launching year of the QOF in 2004.

Figure 2 Bias-corrected canonical correlations (95%CI) using 3-year window for incident clinical code usage for 18 mental and physical conditions

CHD, coronary heart disease; CKD, chronic kidney disease; COPD, chronic obstructive pulmonary disease; DM, diabetes mellitus; HF, heart failure; HT, hypertension; SMI, severe mental illness.
Incident code: a clinical code indicating new (incident) cases. The red line represents the launching year of the QOF in 2004.

Figure 3 Entropy (95% CI) of incident and all clinical code usage for 18 mental and physical conditions

CHD, coronary heart disease; CKD, chronic kidney disease; COPD, chronic obstructive pulmonary disease; DM, diabetes mellitus; HF, heart failure; HT, hypertension; SMI, severe mental illness.
Incident code: a clinical code indicating new (incident) cases. All codes: any diagnostic clinical code for the condition incident and prevalent cases). The red line represents the launching year of the QOF in 2004. The 95% CIs were calculated as the mean +/- 1.96*SE (SE has been estimated using Jackknife approach).

Figure 4 Richness of incident and all clinical code usage for 18 mental and physical conditions

CHD, coronary heart disease; CKD, chronic kidney disease; COPD, chronic obstructive pulmonary disease; DM, diabetes mellitus; HF, heart failure; HT, hypertension; SMI, severe mental illness.
Incident code: a clinical code indicating new (incident) cases. All codes: any diagnostic clinical code for the condition incident and prevalent cases). The red line represents the launching year of the QOF in 2004.

Figure 5 Evenness (95% CI) of incident and all clinical code usage for 18 mental and physical conditions

CHD, coronary heart disease; CKD, chronic kidney disease; COPD, chronic obstructive pulmonary disease; DM, diabetes mellitus; HF, heart failure; HT, hypertension; SMI, severe mental illness.
Incident code: a clinical code indicating new (incident) cases. All codes: any diagnostic clinical code for the condition incident and prevalent cases). The red line represents the launching year of the QOF in 2004. The 95% CIs were calculated as the mean +/- 1.96*SE (SE has been estimated using Jackknife approach).

For peer review only - http://bmjopen.bmj.com/site/about/guidelines.xhtml

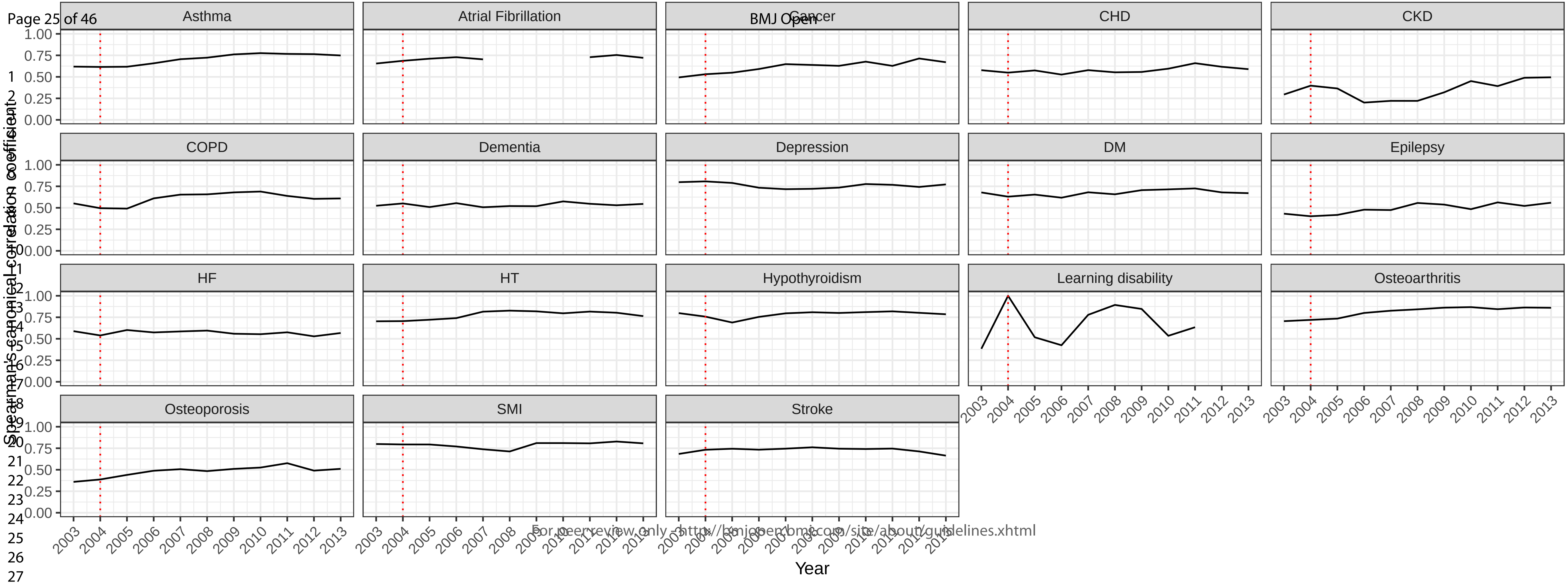

For peer review only - http://bmjopen.bmj.com/site/about/guidelines.xhtml

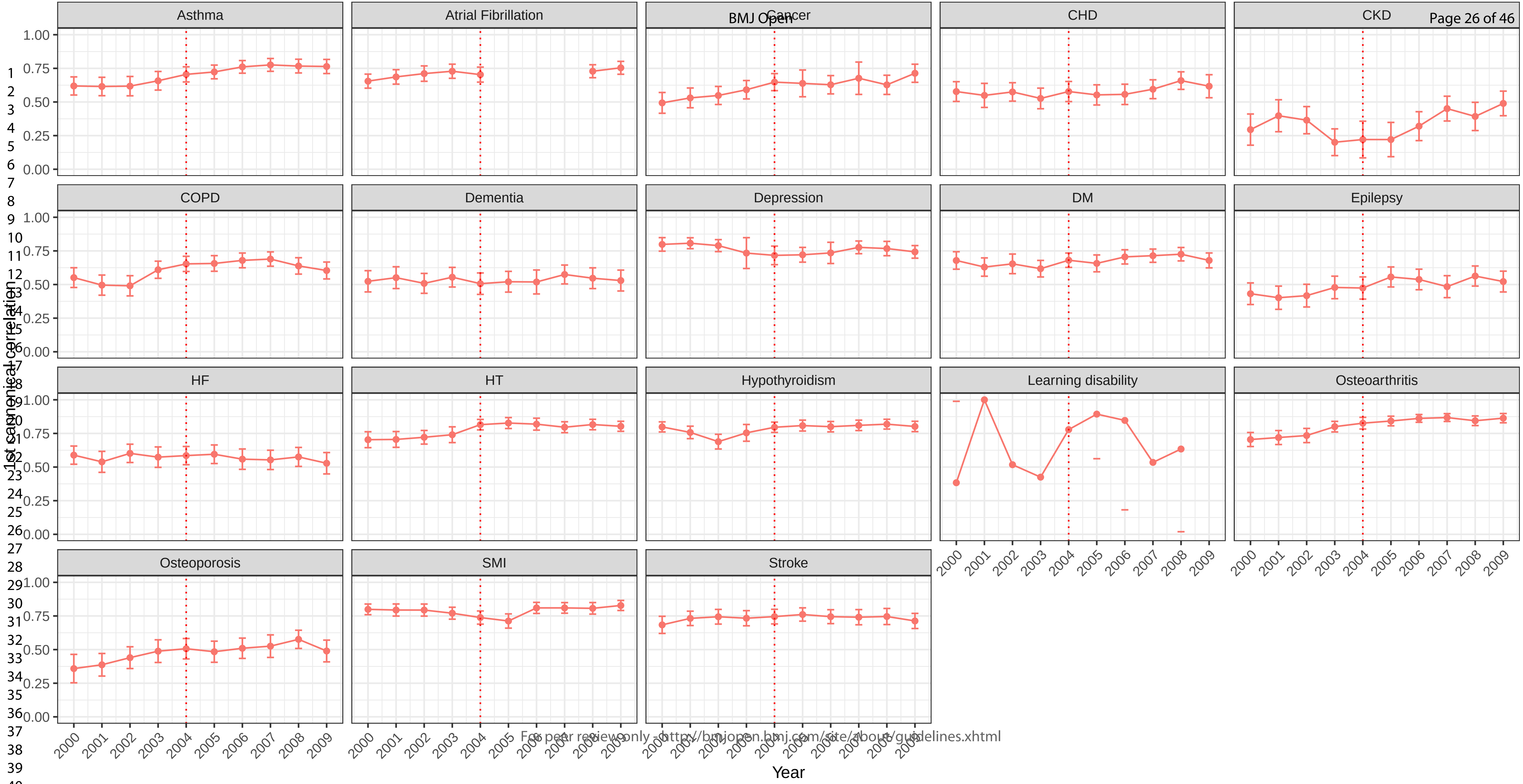
Asthma
Atrial Fibrillation
Cancer
CHD
CKD
COPD
Dementia
Depression
DM
Epilepsy
HF
HT
Hypothyroidism
Learning disability
Osteoarthritis
Osteoporosis
SMI
Stroke
1st canonical correlation
Year
0.00
0.25
0.50
0.75
1.00
2000
2001
2002
2003
2004
2005
2006
2007
2008
2009

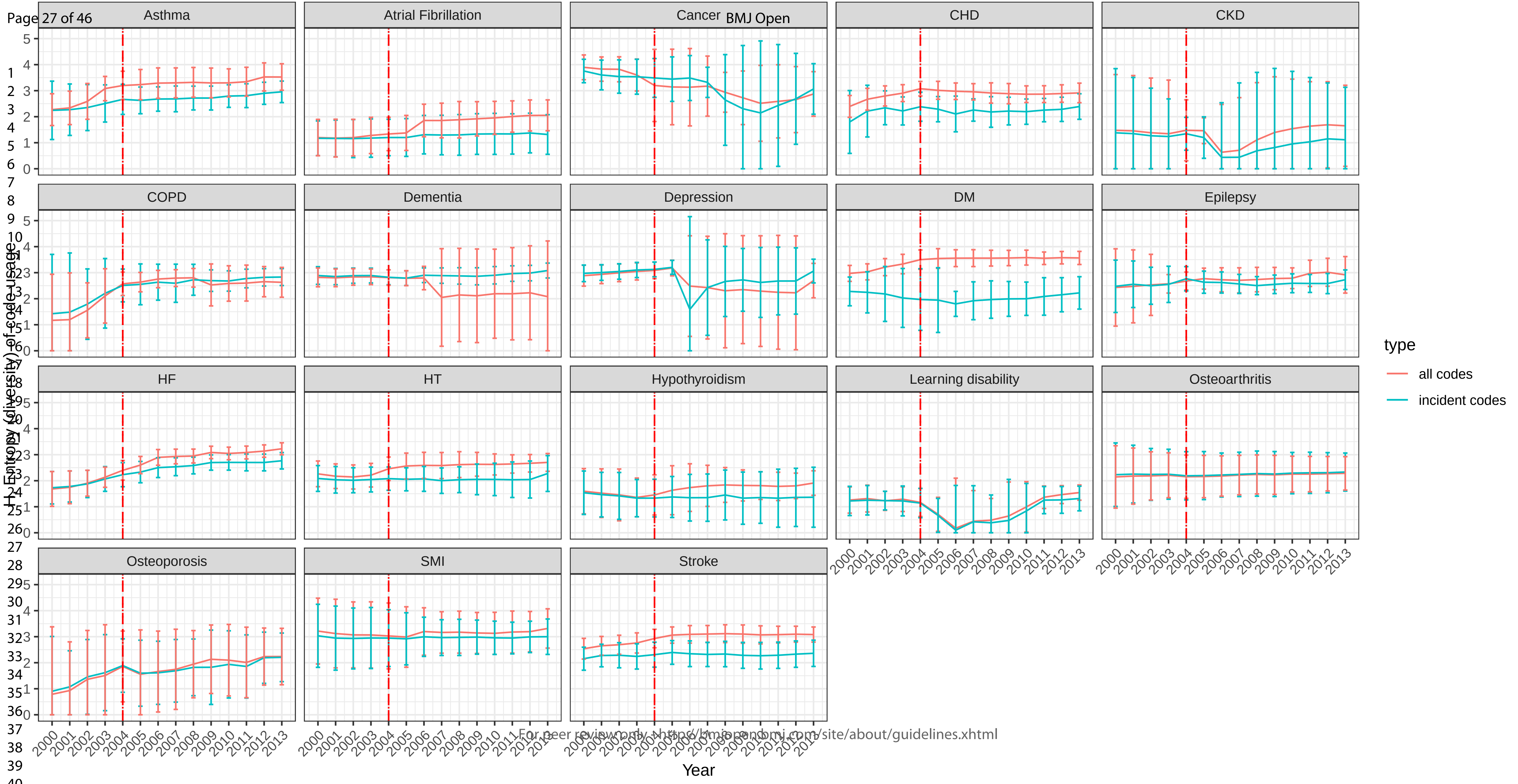
Asthma
Atrial Fibrillation
Cancer
CHD
CKD
COPD
Dementia
Depression
DM
Epilepsy
HF
HT
Hypothyroidism
Learning disability
Osteoarthritis
Osteoporosis
SMI
Stroke
H: Entropy (diversity) of code usage
Year
type
all codes
incident codes
0 1 2 3 4 5
2000 2001 2002 2003 2004 2005 2006 2007 2008 2009 2010 2011 2012 2013

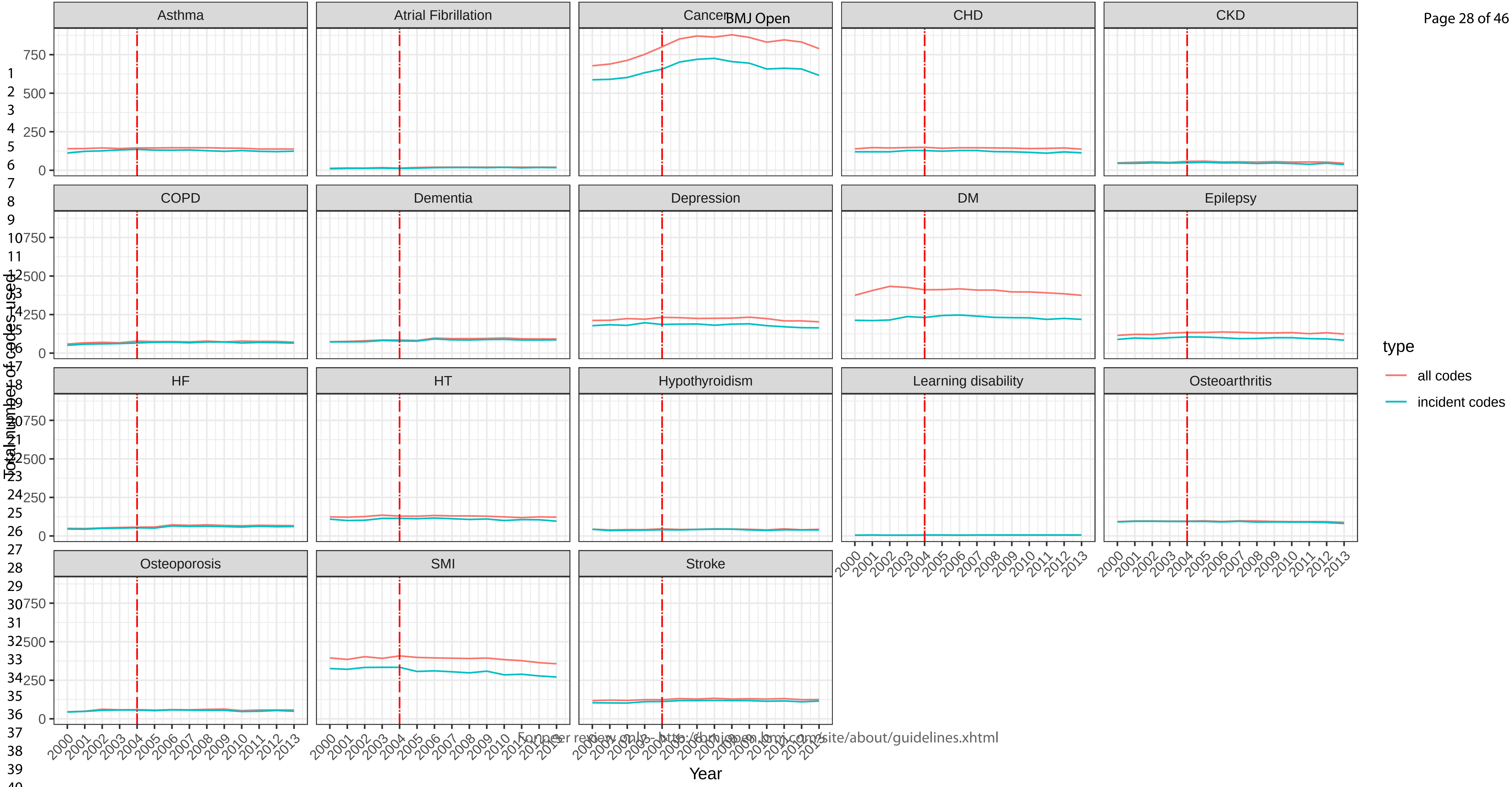
Asthma
Atrial Fibrillation
Cancer
CHD
CKD
COPD
Dementia
Depression
DM
Epilepsy
HF
HT
Hypothyroidism
Learning disability
Osteoarthritis
Osteoporosis
SMI
Stroke
0
250
500
750
2000
2001
2002
2003
2004
2005
2006
2007
2008
2009
2010
2011
2012
2013
Total number of codes used
Year
type
all codes
incident codes

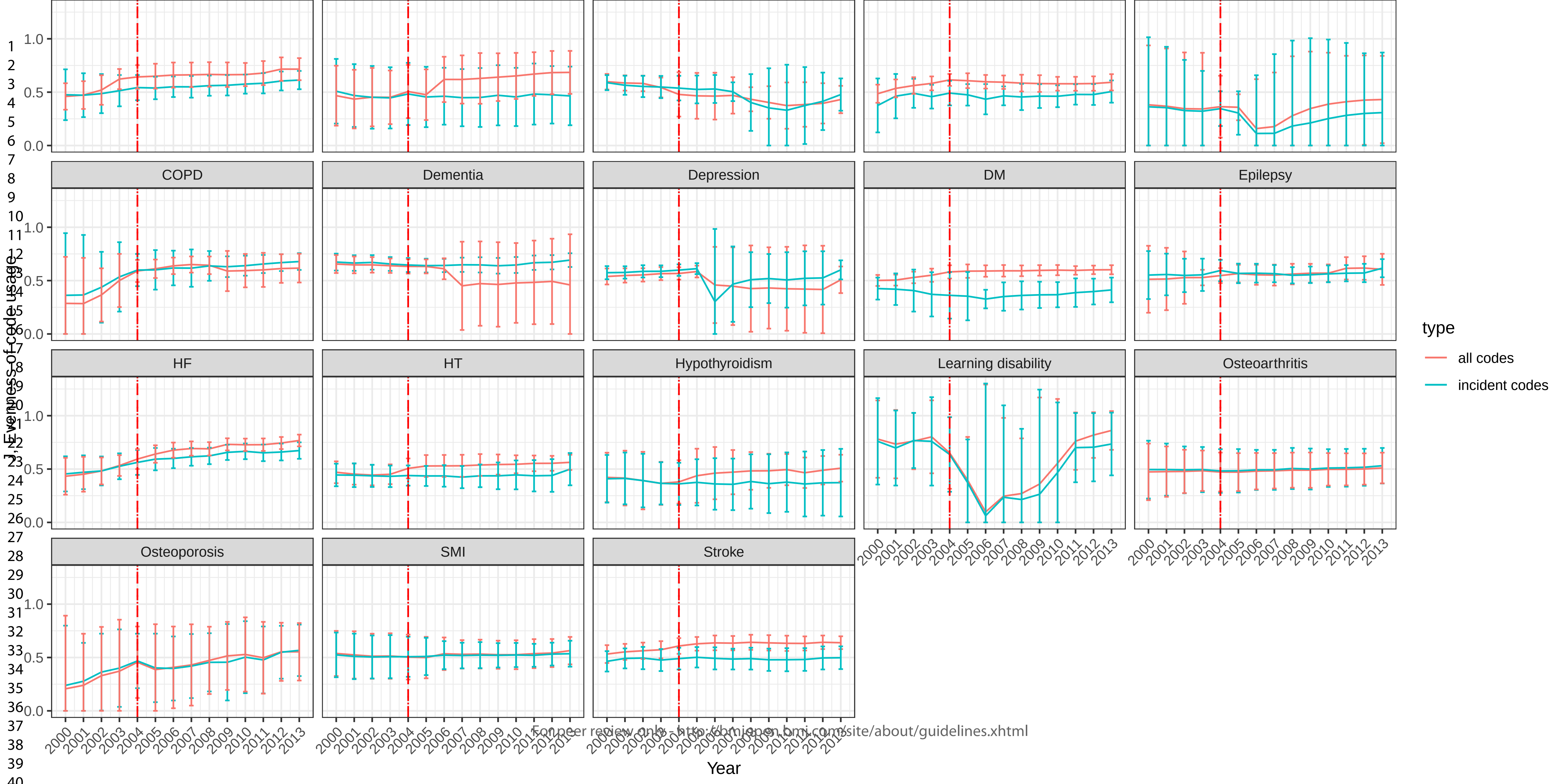
Asthma
Atrial Fibrillation
Cancer
CHD
CKD
COPD
Dementia
Depression
DM
Epilepsy
HF
HT
Hypothyroidism
Learning disability
Osteoarthritis
Osteoporosis
SMI
Stroke
J. Evenness of code usage
Year
type
all codes
incident codes

Supplementary data to "Clinical code usage in UK general practice: a cohort study exploring 18 conditions over 14 years", Zghebi et al. 2021.

For peer review only

Supplementary data

For peer review only - http://bmjopen.bmj.com/site/about/guidelines.xhtml

Supplementary data to "Clinical code usage in UK general practice: a cohort study exploring 18 conditions over 14 years", Zghebi et al. 2021.

Table S1 Diabetes Read code list

	Code	Coding system	Description
1.	66A3.00	Read	Diabetic on diet only
2.	66A4.00	Read	Diabetic on oral treatment
3.	66A5.00	Read	Diabetic on insulin
4.	66AI.00	Read	Diabetic - good control
5.	66AJ.00	Read	Diabetic - poor control
6.	66AJ100	Read	Brittle diabetes
7.	66AJ.11	Read	Unstable diabetes
8.	66AJz00	Read	Diabetic - poor control NOS
9.	66AK.00	Read	Diabetic - cooperative patient
10.	66AL.00	Read	Diabetic-uncooperative patient
11.	66AV.00	Read	Diabetic on insulin and oral treatment
12.	C10..00	Read	Diabetes mellitus
13.	C100.00	Read	Diabetes mellitus with no mention of complication
14.	C100000	Read	Diabetes mellitus; juvenile type; no mention of complication
15.	C100011	Read	Insulin dependent diabetes mellitus
16.	C100100	Read	Diabetes mellitus; adult onset; no mention of complication
17.	C100111	Read	Maturity onset diabetes
18.	C100112	Read	Non-insulin dependent diabetes mellitus
19.	C100z00	Read	Diabetes mellitus NOS with no mention of complication
20.	C101.00	Read	Diabetes mellitus with ketoacidosis
21.	C101000	Read	Diabetes mellitus; juvenile type; with ketoacidosis
22.	C101100	Read	Diabetes mellitus; adult onset; with ketoacidosis
23.	C101y00	Read	Other specified diabetes mellitus with ketoacidosis
24.	C101z00	Read	Diabetes mellitus NOS with ketoacidosis
25.	C102.00	Read	Diabetes mellitus with hyperosmolar coma
26.	C102000	Read	Diabetes mellitus; juvenile type; with hyperosmolar coma
27.	C102100	Read	Diabetes mellitus; adult onset; with hyperosmolar coma
28.	C102z00	Read	Diabetes mellitus NOS with hyperosmolar coma
29.	C103.00	Read	Diabetes mellitus with ketoacidotic coma
30.	C103000	Read	Diabetes mellitus; juvenile type; with ketoacidotic coma
31.	C103100	Read	Diabetes mellitus; adult onset; with ketoacidotic coma
32.	C103y00	Read	Other specified diabetes mellitus with coma
33.	C103z00	Read	Diabetes mellitus NOS with ketoacidotic coma
34.	C104.00	Read	Diabetes mellitus with renal manifestation
35.	C104000	Read	Diabetes mellitus; juvenile type; with renal manifestation
36.	C104100	Read	Diabetes mellitus; adult onset; with renal manifestation
37.	C104y00	Read	Other specified diabetes mellitus with renal complications
38.	C104z00	Read	Diabetes mellitis with nephropathy NOS
39.	C105.00	Read	Diabetes mellitus with ophthalmic manifestation
40.	C105000	Read	Diabetes mellitus; juvenile type; + ophthalmic manifestation
41.	C105100	Read	Diabetes mellitus; adult onset; + ophthalmic manifestation
42.	C105y00	Read	Other specified diabetes mellitus with ophthalmic complicatn
43.	C105z00	Read	Diabetes mellitus NOS with ophthalmic manifestation
44.	C106.00	Read	Diabetes mellitus with neurological manifestation
45.	C106000	Read	Diabetes mellitus; juvenile; + neurological manifestation
46.	C106100	Read	Diabetes mellitus; adult onset; + neurological manifestation
47.	C106.12	Read	Diabetes mellitus with neuropathy

For peer review only - http://bmjopen.bmj.com/site/about/guidelines.xhtml

Supplementary data to "Clinical code usage in UK general practice: a cohort study exploring 18 conditions over 14 years", Zghebi et al. 2021.

48.	C106.13	Read	Diabetes mellitus with polyneuropathy
49.	C106y00	Read	Other specified diabetes mellitus with neurological comps
50.	C106z00	Read	Diabetes mellitus NOS with neurological manifestation
51.	C107.00	Read	Diabetes mellitus with peripheral circulatory disorder
52.	C107000	Read	Diabetes mellitus; juvenile +peripheral circulatory disorder
53.	C107100	Read	Diabetes mellitus; adult; + peripheral circulatory disorder
54.	C107.11	Read	Diabetes mellitus with gangrene
55.	C107.12	Read	Diabetes with gangrene
56.	C107200	Read	Diabetes mellitus; adult with gangrene
57.	C107300	Read	IDDM with peripheral circulatory disorder
58.	C107400	Read	NIDDM with peripheral circulatory disorder
59.	C107z00	Read	Diabetes mellitus NOS with peripheral circulatory disorder
60.	C108.00	Read	Insulin dependent diabetes mellitus
61.	C108000	Read	Insulin-dependent diabetes mellitus with renal complications
62.	C108011	Read	Type I diabetes mellitus with renal complications
63.	C108012	Read	Type 1 diabetes mellitus with renal complications
64.	C108100	Read	Insulin-dependent diabetes mellitus with ophthalmic comps
65.	C108.11	Read	IDDM-Insulin dependent diabetes mellitus
66.	C108.12	Read	Type 1 diabetes mellitus
67.	C108.13	Read	Type I diabetes mellitus
68.	C108200	Read	Insulin-dependent diabetes mellitus with neurological comps
69.	C108211	Read	Type I diabetes mellitus with neurological complications
70.	C108212	Read	Type 1 diabetes mellitus with neurological complications
71.	C108300	Read	Insulin dependent diabetes mellitus with multiple complicatn
72.	C108400	Read	Unstable insulin dependent diabetes mellitus
73.	C108411	Read	Unstable type I diabetes mellitus
74.	C108500	Read	Insulin dependent diabetes mellitus with ulcer
75.	C108511	Read	Type I diabetes mellitus with ulcer
76.	C108600	Read	Insulin dependent diabetes mellitus with gangrene
77.	C108700	Read	Insulin dependent diabetes mellitus with retinopathy
78.	C108711	Read	Type I diabetes mellitus with retinopathy
79.	C108712	Read	Type 1 diabetes mellitus with retinopathy
80.	C108800	Read	Insulin dependent diabetes mellitus - poor control
81.	C108811	Read	Type I diabetes mellitus - poor control
82.	C108812	Read	Type 1 diabetes mellitus - poor control
83.	C108900	Read	Insulin dependent diabetes maturity onset
84.	C108911	Read	Type I diabetes mellitus maturity onset
85.	C108A00	Read	Insulin-dependent diabetes without complication
86.	C108B00	Read	Insulin dependent diabetes mellitus with mononeuropathy
87.	C108B11	Read	Type I diabetes mellitus with mononeuropathy
88.	C108C00	Read	Insulin dependent diabetes mellitus with polyneuropathy
89.	C108D00	Read	Insulin dependent diabetes mellitus with nephropathy
90.	C108D11	Read	Type I diabetes mellitus with nephropathy
91.	C108E00	Read	Insulin dependent diabetes mellitus with hypoglycaemic coma
92.	C108E11	Read	Type I diabetes mellitus with hypoglycaemic coma
93.	C108E12	Read	Type 1 diabetes mellitus with hypoglycaemic coma
94.	C108F00	Read	Insulin dependent diabetes mellitus with diabetic cataract
95.	C108F11	Read	Type I diabetes mellitus with diabetic cataract
96.	C108G00	Read	Insulin dependent diab mell with peripheral angiopathy
97.	C108H00	Read	Insulin dependent diabetes mellitus with arthropathy

For peer review only - http://bmjopen.bmj.com/site/about/guidelines.xhtml

Supplementary data to "Clinical code usage in UK general practice: a cohort study exploring 18 conditions over 14 years", Zghebi et al. 2021.

98.	C108H11	Read	Type I diabetes mellitus with arthropathy
99.	C108J00	Read	Insulin dependent diab mell with neuropathic arthropathy
100.	C108J12	Read	Type 1 diabetes mellitus with neuropathic arthropathy
101.	C108y00	Read	Other specified diabetes mellitus with multiple comps
102.	C108z00	Read	Unspecified diabetes mellitus with multiple complications
103.	C109.00	Read	Non-insulin-dependent diabetes mellitus
104.	C109000	Read	Non-insulin-dependent diabetes mellitus with renal comps
105.	C109011	Read	Type II diabetes mellitus with renal complications
106.	C109012	Read	Type 2 diabetes mellitus with renal complications
107.	C109100	Read	Non-insulin-dependent diabetes mellitus with ophthalm comps
108.	C109.11	Read	NIDDM - Non-insulin dependent diabetes mellitus
109.	C109111	Read	Type II diabetes mellitus with ophthalmic complications
110.	C109112	Read	Type 2 diabetes mellitus with ophthalmic complications
111.	C109.12	Read	Type 2 diabetes mellitus
112.	C109.13	Read	Type II diabetes mellitus
113.	C109200	Read	Non-insulin-dependent diabetes mellitus with neuro comps
114.	C109211	Read	Type II diabetes mellitus with neurological complications
115.	C109212	Read	Type 2 diabetes mellitus with neurological complications
116.	C109300	Read	Non-insulin-dependent diabetes mellitus with multiple comps
117.	C109400	Read	Non-insulin dependent diabetes mellitus with ulcer
118.	C109411	Read	Type II diabetes mellitus with ulcer
119.	C109412	Read	Type 2 diabetes mellitus with ulcer
120.	C109500	Read	Non-insulin dependent diabetes mellitus with gangrene
121.	C109511	Read	Type II diabetes mellitus with gangrene
122.	C109600	Read	Non-insulin-dependent diabetes mellitus with retinopathy
123.	C109611	Read	Type II diabetes mellitus with retinopathy
124.	C109612	Read	Type 2 diabetes mellitus with retinopathy
125.	C109700	Read	Non-insulin dependant diabetes mellitus - poor control
126.	C109711	Read	Type II diabetes mellitus - poor control
127.	C109712	Read	Type 2 diabetes mellitus - poor control
128.	C109900	Read	Non-insulin-dependent diabetes mellitus without complication
129.	C109A00	Read	Non-insulin dependent diabetes mellitus with mononeuropathy
130.	C109A11	Read	Type II diabetes mellitus with mononeuropathy
131.	C109B00	Read	Non-insulin dependent diabetes mellitus with polyneuropathy
132.	C109B11	Read	Type II diabetes mellitus with polyneuropathy
133.	C109C00	Read	Non-insulin dependent diabetes mellitus with nephropathy
134.	C109C11	Read	Type II diabetes mellitus with nephropathy
135.	C109C12	Read	Type 2 diabetes mellitus with nephropathy
136.	C109D00	Read	Non-insulin dependent diabetes mellitus with hypoglyca coma
137.	C109D11	Read	Type II diabetes mellitus with hypoglycaemic coma
138.	C109D12	Read	Type 2 diabetes mellitus with hypoglycaemic coma
139.	C109E00	Read	Non-insulin depend diabetes mellitus with diabetic cataract
140.	C109E11	Read	Type II diabetes mellitus with diabetic cataract
141.	C109E12	Read	Type 2 diabetes mellitus with diabetic cataract
142.	C109F00	Read	Non-insulin-dependent d m with peripheral angiopath
143.	C109F11	Read	Type II diabetes mellitus with peripheral angiopathy
144.	C109F12	Read	Type 2 diabetes mellitus with peripheral angiopathy
145.	C109G00	Read	Non-insulin dependent diabetes mellitus with arthropathy
146.	C109G11	Read	Type II diabetes mellitus with arthropathy
147.	C109G12	Read	Type 2 diabetes mellitus with arthropathy

For peer review only - http://bmjopen.bmj.com/site/about/guidelines.xhtml

Supplementary data to "Clinical code usage in UK general practice: a cohort study exploring 18 conditions over 14 years", Zghebi et al. 2021.

148.	C109H00	Read	Non-insulin dependent d m with neuropathic arthropathy
149.	C109H11	Read	Type II diabetes mellitus with neuropathic arthropathy
150.	C109H12	Read	Type 2 diabetes mellitus with neuropathic arthropathy
151.	C109J00	Read	Insulin treated Type 2 diabetes mellitus
152.	C109J11	Read	Insulin treated non-insulin dependent diabetes mellitus
153.	C109J12	Read	Insulin treated Type II diabetes mellitus
154.	C109K00	Read	Hyperosmolar non-ketotic state in type 2 diabetes mellitus
155.	C10C.00	Read	Diabetes mellitus autosomal dominant
156.	C10C.11	Read	Maturity onset diabetes in youth
157.	C10D.00	Read	Diabetes mellitus autosomal dominant type 2
158.	C10D.11	Read	Maturity onset diabetes in youth type 2
159.	C10E.00	Read	Type 1 diabetes mellitus
160.	C10E000	Read	Type 1 diabetes mellitus with renal complications
161.	C10E100	Read	Type 1 diabetes mellitus with ophthalmic complications
162.	C10E.11	Read	Type I diabetes mellitus
163.	C10E.12	Read	Insulin dependent diabetes mellitus
164.	C10E200	Read	Type 1 diabetes mellitus with neurological complications
165.	C10E300	Read	Type 1 diabetes mellitus with multiple complications
166.	C10E312	Read	Insulin dependent diabetes mellitus with multiple complicat
167.	C10E400	Read	Unstable type 1 diabetes mellitus
168.	C10E411	Read	Unstable type I diabetes mellitus
169.	C10E412	Read	Unstable insulin dependent diabetes mellitus
170.	C10E500	Read	Type 1 diabetes mellitus with ulcer
171.	C10E600	Read	Type 1 diabetes mellitus with gangrene
172.	C10E700	Read	Type 1 diabetes mellitus with retinopathy
173.	C10E800	Read	Type 1 diabetes mellitus - poor control
174.	C10E812	Read	Insulin dependent diabetes mellitus - poor control
175.	C10E900	Read	Type 1 diabetes mellitus maturity onset
176.	C10EA00	Read	Type 1 diabetes mellitus without complication
177.	C10EA11	Read	Type I diabetes mellitus without complication
178.	C10EB00	Read	Type 1 diabetes mellitus with mononeuropathy
179.	C10EC00	Read	Type 1 diabetes mellitus with polyneuropathy
180.	C10ED00	Read	Type 1 diabetes mellitus with nephropathy
181.	C10EE00	Read	Type 1 diabetes mellitus with hypoglycaemic coma
182.	C10EF00	Read	Type 1 diabetes mellitus with diabetic cataract
183.	C10EG00	Read	Type 1 diabetes mellitus with peripheral angiopathy
184.	C10EH00	Read	Type 1 diabetes mellitus with arthropathy
185.	C10EJ00	Read	Type 1 diabetes mellitus with neuropathic arthropathy
186.	C10EK00	Read	Type 1 diabetes mellitus with persistent proteinuria
187.	C10EL00	Read	Type 1 diabetes mellitus with persistent microalbuminuria
188.	C10EM00	Read	Type 1 diabetes mellitus with ketoacidosis
189.	C10EM11	Read	Type I diabetes mellitus with ketoacidosis
190.	C10EN00	Read	Type 1 diabetes mellitus with ketoacidotic coma
191.	C10EN11	Read	Type I diabetes mellitus with ketoacidotic coma
192.	C10EP00	Read	Type 1 diabetes mellitus with exudative maculopathy
193.	C10EQ00	Read	Type 1 diabetes mellitus with gastroparesis
194.	C10F.00	Read	Type 2 diabetes mellitus
195.	C10F000	Read	Type 2 diabetes mellitus with renal complications
196.	C10F011	Read	Type II diabetes mellitus with renal complications
197.	C10F100	Read	Type 2 diabetes mellitus with ophthalmic complications

For peer review only - http://bmjopen.bmj.com/site/about/guidelines.xhtml

Supplementary data to "Clinical code usage in UK general practice: a cohort study exploring 18 conditions over 14 years", Zghebi et al. 2021.

198.	C10F.11	Read	Type II diabetes mellitus
199.	C10F200	Read	Type 2 diabetes mellitus with neurological complications
200.	C10F300	Read	Type 2 diabetes mellitus with multiple complications
201.	C10F311	Read	Type II diabetes mellitus with multiple complications
202.	C10F400	Read	Type 2 diabetes mellitus with ulcer
203.	C10F500	Read	Type 2 diabetes mellitus with gangrene
204.	C10F511	Read	Type II diabetes mellitus with gangrene
205.	C10F600	Read	Type 2 diabetes mellitus with retinopathy
206.	C10F611	Read	Type II diabetes mellitus with retinopathy
207.	C10F700	Read	Type 2 diabetes mellitus - poor control
208.	C10F711	Read	Type II diabetes mellitus - poor control
209.	C10F900	Read	Type 2 diabetes mellitus without complication
210.	C10F911	Read	Type II diabetes mellitus without complication
211.	C10FA00	Read	Type 2 diabetes mellitus with mononeuropathy
212.	C10FB00	Read	Type 2 diabetes mellitus with polyneuropathy
213.	C10FB11	Read	Type II diabetes mellitus with polyneuropathy
214.	C10FC00	Read	Type 2 diabetes mellitus with nephropathy
215.	C10FC11	Read	Type II diabetes mellitus with nephropathy
216.	C10FD00	Read	Type 2 diabetes mellitus with hypoglycaemic coma
217.	C10FE00	Read	Type 2 diabetes mellitus with diabetic cataract
218.	C10FF00	Read	Type 2 diabetes mellitus with peripheral angiopathy
219.	C10FG00	Read	Type 2 diabetes mellitus with arthropathy
220.	C10FH00	Read	Type 2 diabetes mellitus with neuropathic arthropathy
221.	C10FJ00	Read	Insulin treated Type 2 diabetes mellitus
222.	C10FJ11	Read	Insulin treated Type II diabetes mellitus
223.	C10FK00	Read	Hyperosmolar non-ketotic state in type 2 diabetes mellitus
224.	C10FL00	Read	Type 2 diabetes mellitus with persistent proteinuria
225.	C10FL11	Read	Type II diabetes mellitus with persistent proteinuria
226.	C10FM00	Read	Type 2 diabetes mellitus with persistent microalbuminuria
227.	C10FN00	Read	Type 2 diabetes mellitus with ketoacidosis
228.	C10FP00	Read	Type 2 diabetes mellitus with ketoacidotic coma
229.	C10FQ00	Read	Type 2 diabetes mellitus with exudative maculopathy
230.	C10FR00	Read	Type 2 diabetes mellitus with gastroparesis
231.	C10G.00	Read	Secondary pancreatic diabetes mellitus
232.	C10G000	Read	Secondary pancreatic diabetes mellitus without complication
233.	C10y.00	Read	Diabetes mellitus with other specified manifestation
234.	C10y100	Read	Diabetes mellitus; adult; + other specified manifestation
235.	C10yy00	Read	Other specified diabetes mellitus with other spec comps
236.	C10yz00	Read	Diabetes mellitus NOS with other specified manifestation
237.	C10z.00	Read	Diabetes mellitus with unspecified complication
238.	C10z000	Read	Diabetes mellitus; juvenile type; + unspecified complication
239.	C10z100	Read	Diabetes mellitus; adult onset; + unspecified complication
240.	C10zy00	Read	Other specified diabetes mellitus with unspecified comps
241.	C10zz00	Read	Diabetes mellitus NOS with unspecified complication
242.	Cyu2.00	Read	[X]Diabetes mellitus
243.	Cyu2000	Read	[X]Other specified diabetes mellitus
244.	Cyu2300	Read	[X]Unspecified diabetes mellitus with renal complications
245.	L180500	Read	Pre-existing diabetes mellitus; insulin-dependent
246.	L180600	Read	Pre-existing diabetes mellitus; non-insulin-dependent
247.	L180X00	Read	Pre-existing diabetes mellitus; unspecified

For peer review only - http://bmjopen.bmj.com/site/about/guidelines.xhtml

Supplementary data to "Clinical code usage in UK general practice: a cohort study exploring 18 conditions over 14 years", Zghebi et al. 2021.

Table S2 R code

1, 3, 5, year Jackknife correlations (Figures 2, S3, S4)

```
library(dplyr)
library(tidyr)
library(ggplot2)
library(stringr)

setwd("Q:/code_usage")
rm(list=ls())
conditions <- c("asthma", "atrial_fibrillation", "cancer", "chd", "ckd",
            "copd", "dementia", "depression", "dm", "epilepsy", "hf", "ht","hypothyroidism" ,
            "learning_disability", "osteoarthritis", "osteoporosis", "smi", "stroke")


jack_incidence <- new.env()
jack_total <- new.env()

for(condition in conditions){
  if(file.exists(myfile <- paste0("data/", condition, "_year_cors.rda"))){
    message("Loading ", myfile)
    load(myfile, envir = jack_incidence)
  }
  if(file.exists(myfile <- paste0("data/", condition, "_total_cors.rda"))){
    message("Loading ", myfile)
    load(myfile, envir = jack_total)
  }

}

incid_data <- bind_rows(lapply(ls(jack_incidence), function(condition){
  bind_rows(jack_incidence[[condition]]) %>%
    mutate(condition = str_replace(condition, "_year_cors", ""),
         type = "incident codes")
}))

total_data <- bind_rows(lapply(ls(jack_total), function(condition){
  bind_rows(jack_total[[condition]]) %>%
    mutate(condition = str_replace(condition, "_total_cors", ""),
         type = "all codes")
}))

jack_data <- bind_rows(incid_data, total_data) %>%
  mutate(year1 = as.numeric(year1))


## Year-on-year correlations:
p <- ggplot(jack_data %>% filter(num < 14),
        aes(x = year1, y = cor, colour = type))
p + geom_point() +
  geom_line() +
```


For peer review only - http://bmjopen.bmj.com/site/about/guidelines.xhtml

Supplementary data to "Clinical code usage in UK general practice: a cohort study exploring 18 conditions over 14 years", Zghebi et al. 2021.

```
    geom_errorbar(aes(ymin = lower, ymax = upper), width = 0.25) +
    facet_wrap( ~ condition) +
    theme_bw() +
    scale_x_continuous("Year", breaks = c(2000, 2002, 2004, 2006, 2008, 2010, 2012)) +
    scale_y_continuous("1st cannonical correlation", limits = c(0,1)) +
    theme(axis.text.x = element_text(angle = 45, hjust = 1)) +
    #labs(title = "")
ggsave("analysis/1-jack_corr_1yearwindow.pdf")


## Bi-yearly correlations:
p <- ggplot(jack_data %>% filter(num >= 14, num < 24),
         aes(x = year1, y = cor, colour = type))
p + geom_point() +
    geom_line() +
    geom_errorbar(aes(ymin = lower, ymax = upper), width = 0.25) +
    facet_wrap( ~ condition) +
    theme_bw() +
    scale_x_continuous("Year", breaks = c(2000, 2002, 2004, 2006, 2008, 2010, 2012)) +
    scale_y_continuous("1st cannonical correlation",
                 limits = c(0,1)) +
    theme(axis.text.x = element_text(angle = 45, hjust = 1)) +
    #labs(title = "")
ggsave("analysis/2-jack_corr_3yearwindow.pdf")

## 5-yearly correlations:
p <- ggplot(jack_data %>% filter(num >= 14, num < 24),
         aes(x = year1, y = cor, colour = type))
p + geom_point() +
    geom_line() +
    geom_errorbar(aes(ymin = lower, ymax = upper), width = 0.25) +
    facet_wrap( ~ condition) +
    theme_bw() +
    scale_x_continuous("Year", breaks = c(2000, 2002, 2004, 2006, 2008, 2010, 2012)) +
    scale_y_continuous("1st cannonical correlation", limits = c(0,1)) +
    #labs(title = "")
ggsave("analysis/3-jack_corr_5yearwindow.pdf")
```

Canonical correlations 5-year windows (for Figure S2)

```
library(rEHR)
library(dplyr)
library(tidyr)
library(ggplot2)

rm(list=ls())
conditions <- c("asthma", "atrial_fibrillation", "cancer", "chd", "ckd",
            "copd", "dementia", "depression", "dm", "epilepsy", "hf", "ht","hypothyroidism" ,
            "learning_disability", "osteoarthritis", "osteoporosis", "smi", "stroke")
correlations <- new.env()
```


For peer review only - http://bmjopen.bmj.com/site/about/guidelines.xhtml

Supplementary data to "Clinical code usage in UK general practice: a cohort study exploring 18 conditions over 14 years", Zghebi et al. 2021.

```
for(condition in conditions){
   load(paste0("data/", condition, "_year_cors.rda"), envir = correlations)
   my_corr <- get(paste0(condition, "_year_cors"),  envir = correlations)
   my_corr <-  lapply(my_corr, function(x) {
        x$condition <- condition
        x
   })
   assign(paste0(condition, "_year_cors"), my_corr, envir = correlations)
}
rm(my_corr)

all_corrs <- bind_rows(lapply(correlations, function(corr){
   data_frame(year = 2005:2013,
            cor = unlist(lapply(corr, function(x) x$cor)),
            condition = corr[[1]]$condition)
}))


p <- ggplot(all_corrs, aes(x = year, y = cor))
p + geom_line() +
   facet_wrap( ~ condition) +
#    geom_vline(x = 2004, linetype  = "longdash", colour = "red", width = 4) +
   theme_bw() +
   labs(title  = "Correlations of clinical code usage (Five year windows)") +
   scale_y_continuous("Spearman's canonical correlation coefficient") +
   scale_x_continuous(breaks = c(2004, 2006, 2008, 2010, 2012)) +
   theme(axis.text.x = element_text(angle = 45, hjust = 1))

ggsave("analysis/canonical_correlations_5yearwindows.pdf")
```

Three diversity measures (Figures 3, 4, 5)

```
library(rEHR)
library(dplyr)
library(tidyr)
library(parallel)
library(ggplot2)
library(bootstrap)

setwd("Q:/code_usage")
#' calculates the Shannon index of diversity/entropy
shannon <- function(x)
{
   x <- drop(as.matrix(x))
   if (length(dim(x)) > 1) {
      total <- apply(x, 1, sum)
      x <- sweep(x, 1, total, "/")
   }
   else {
      x <- x/sum(x)
   }
```

For peer review only

Supplementary data to "Clinical code usage in UK general practice: a cohort study exploring 18 conditions over 14 years", Zghebi et al. 2021.

```
    x <- -x * log(x)

    if (length(dim(x)) > 1)
        H <- apply(x, sum, na.rm = TRUE)
    else H <- sum(x, na.rm = TRUE)
    H
}

evenness_fn <- function(x){
    H <- shannon(x)
    R <- length(x)
    H / log(R)
}


jack_stats <- function(x, thetahat){
    n <- length(x)
    bias <- (n - 1) * (mean(x) - thetahat)
    se <- sqrt(((n - 1)/n) * sum((x - mean(x))^2))
    list(param = thetahat, bias = bias, se = se)
}

conditions <- c("asthma", "atrial_fibrillation", "blood_pressure", "cancer", "chd", "ckd",
                "copd", "dementia", "depression", "dm", "epilepsy", "hf", "ht","hypothyroidism" ,
                "learning_disability", "osteoarthritis", "osteoporosis", "smi", "stroke")
frequencies <- new.env()

for(condition in conditions){
    load(paste0("data/", condition, "_frequencies.rda"), envir = frequencies)
    incid <- get(paste0(condition, "_incidence_freqs"),  envir = frequencies)
    incid$type <- "incident codes"
    assign(paste0(condition, "_incidence_freqs"), incid, envir = frequencies)

    total <- get(paste0(condition, "_total_freqs"),  envir = frequencies)
    total$type <- "all codes"
    assign(paste0(condition, "_total_freqs"), total, envir = frequencies)

}

all_freqs <- bind_rows(lapply(frequencies, function(x) x))

years <- 2000:2013
jacks <- bind_rows(lapply(frequencies, function(condition){
    bind_rows(mclapply(years, function(this_year){
        dat <- condition %>%
            filter(year == this_year)
        H <- shannon(dat$freq)
        H_jack <- jackknife(dat$freq, theta = shannon)
        richness <- nrow(dat)
        evenness <- evenness_fn(dat$freq)
        evenness_jack <- jackknife(dat$freq, theta = evenness_fn)
        data_frame(year = rep(this_year, 3),
                   condition = rep(dat$condition[1], 3),
```


For peer review only - http://bmjopen.bmj.com/site/about/guidelines.xhtml

Supplementary data to "Clinical code usage in UK general practice: a cohort study exploring 18 conditions over 14 years", Zghebi et al. 2021.

```
          type = rep(dat$type[1], 3),
          index = c("H", "richness", "evenness"),
          value = c(H, richness, evenness),
          upper = c(H + 1.96 * H_jack$jack.se,
               NA,
               evenness + 1.96 * evenness_jack$jack.se),
          lower = c(H - 1.96 * H_jack$jack.se,
               NA,
               evenness - 1.96 * evenness_jack$jack.se),
          bias = c(H_jack$jack.bias, NA, evenness_jack$jack.bias))

  }, mc.cores = 1))
}))

p <- ggplot(jacks %>% filter(index == "H", condition != "blood_pressure"),
      aes(x = year, y = value, colour = type))
p + geom_line() +
  facet_wrap( ~ condition) +
  geom_errorbar(aes(ymin = lower, ymax = upper), width = 0.25) +
  #geom_vline(x = 2004, linetype  = "longdash", colour = "red", width = 4) +
  theme_bw() +
  labs(title  = "Entropy of clinical code usage") +
  scale_y_continuous("H, Entropy (diversity) of code useage")
ggsave("analysis/4-entropy.pdf")
ggsave("analysis/4-entropy.png")

p <- ggplot(jacks %>% filter(index == "richness", condition != "blood_pressure"),
      aes(x = year, y = value, colour = type))
p + geom_line() +
  facet_wrap( ~ condition) +
  geom_errorbar(aes(ymin = lower, ymax = upper), width = 0.25) +
  #geom_vline(x = 2004, linetype  = "longdash", colour = "red", width = 4) +
  theme_bw() +
  labs(title  = "Richness of clinical code usage") +
  scale_y_continuous("Total number of codes used")
ggsave("analysis/5-richness.pdf")
ggsave("analysis/5-richness.png")


p <- ggplot(jacks %>% filter(index == "evenness", condition != "blood_pressure"),
      aes(x = year, y = value, colour = type))
p + geom_line() +
  facet_wrap( ~ condition) +
  geom_errorbar(aes(ymin = lower, ymax = upper), width = 0.25) +
  #geom_vline(x = 2004, linetype  = "longdash", colour = "red", width = 4) +
  theme_bw() +
  labs(title  = "Evenness of clinical code usage") +
  scale_y_continuous("J, Evenness of code useage")
ggsave("analysis/6-evenness.pdf")
ggsave("analysis/6-evenness.png")

save(jacks, file = "data/jackknife_entropy.rda")
```


For peer review only
For peer review only - http://bmjopen.bmj.com/site/about/guidelines.xhtml

Supplementary data to "Clinical code usage in UK general practice: a cohort study exploring 18 conditions over 14 years", Zghebi et al. 2021.

Figure S1 Canonical correlation using 1-year window of clinical code usage for 18 mental and physical conditions

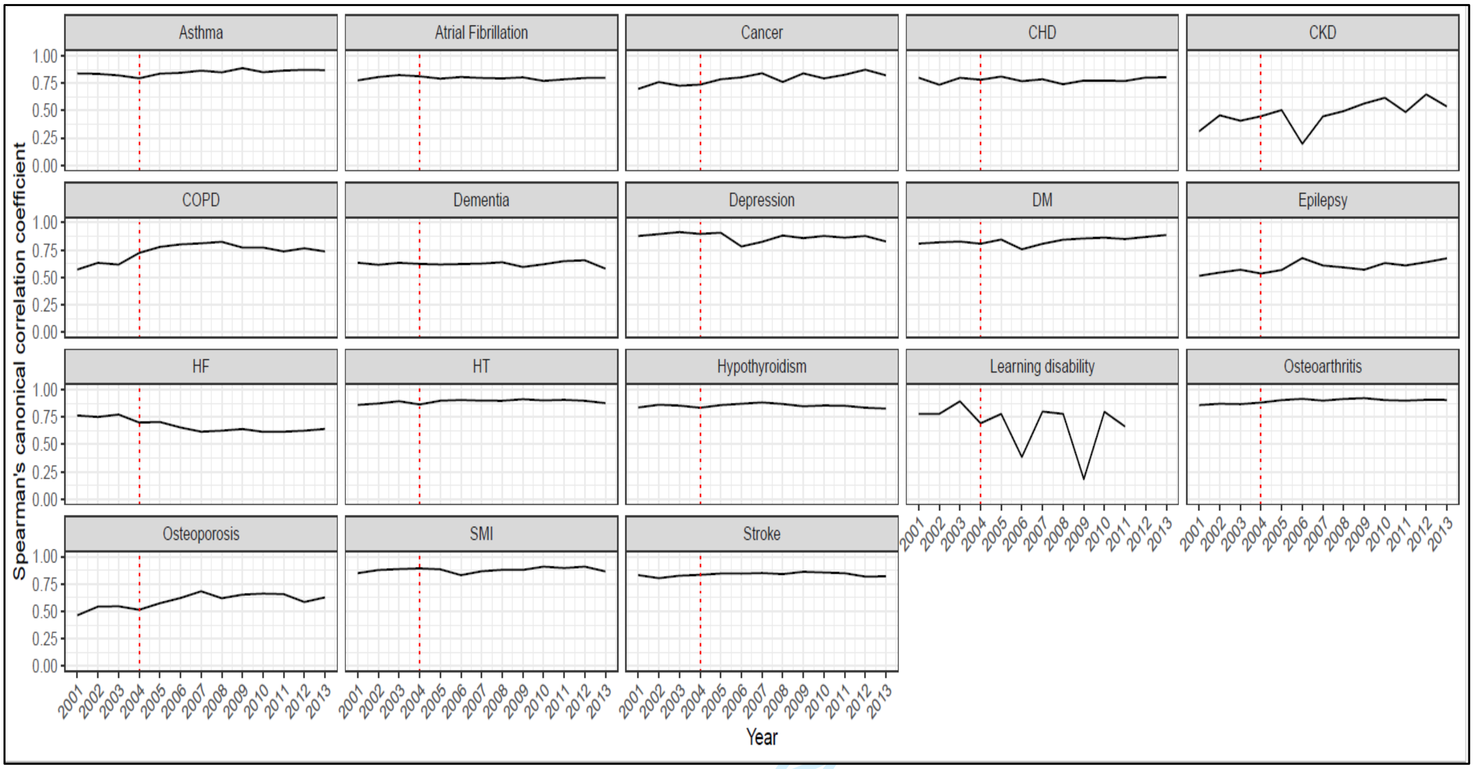

CHD, coronary heart disease; CKD, chronic kidney disease; COPD, chronic obstructive pulmonary disease; DM, diabetes mellitus; HF, heart failure; HT, hypertension; SMI, severe mental illness. The red line represents the launching year of the QOF in 2004.

For peer review only - http://bmjopen.bmj.com/site/about/guidelines.xhtml

Supplementary data to "Clinical code usage in UK general practice: a cohort study exploring 18 conditions over 14 years", Zghebi et al. 2021.

Figure S2 Canonical correlation using 5-year window of clinical code usage for 18 mental and physical conditions

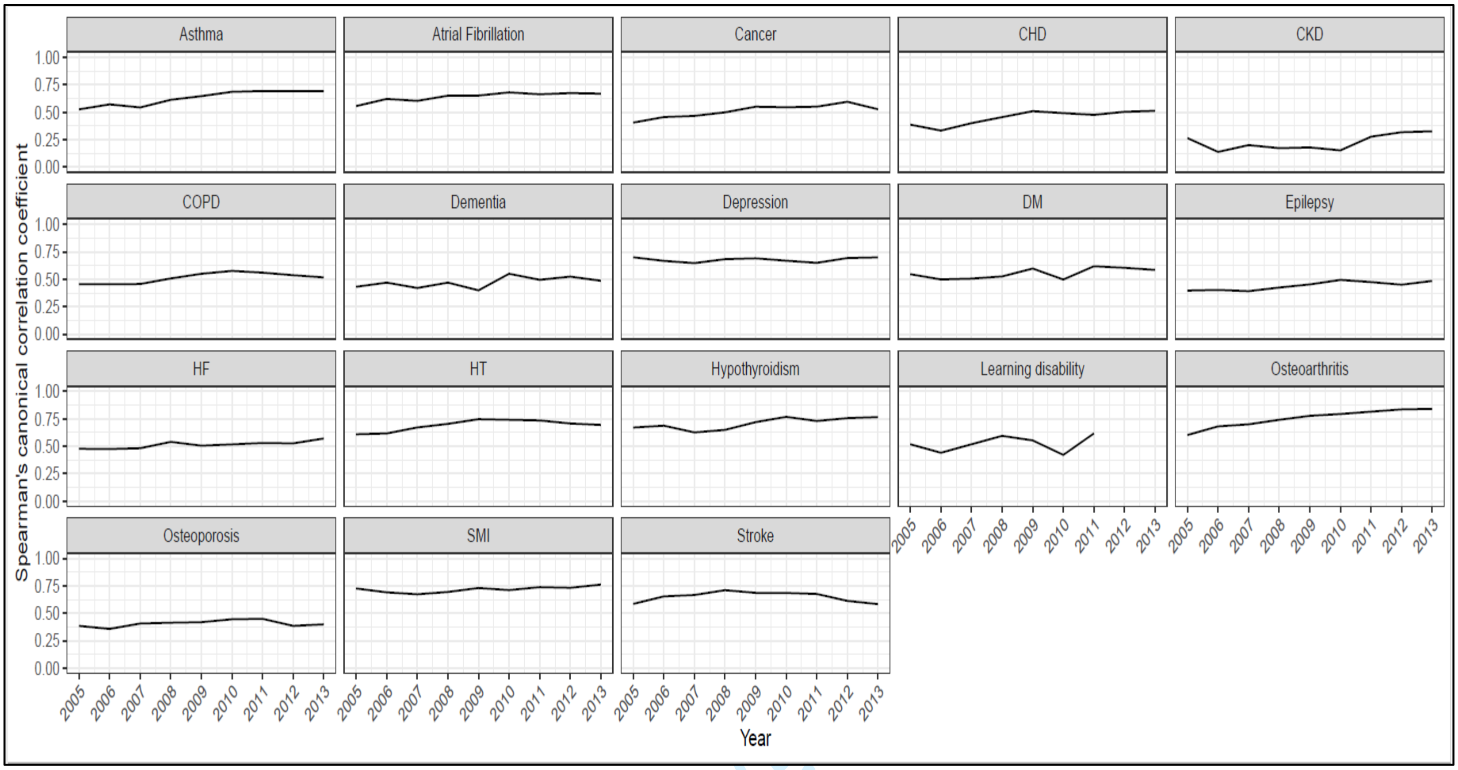

CHD, coronary heart disease; CKD, chronic kidney disease; COPD, chronic obstructive pulmonary disease; DM, diabetes mellitus; HF, heart failure; HT, hypertension; SMI, severe mental illness. The red line represents the launching year of the QOF in 2004.

For peer review only - http://bmjopen.bmj.com/site/about/guidelines.xhtml

Supplementary data to "Clinical code usage in UK general practice: a cohort study exploring 18 conditions over 14 years", Zghebi et al. 2021.

Figure S3 Bias-corrected canonical correlations (95%CI) using 1-year window for incident clinical code usage for 18 mental and physical conditions

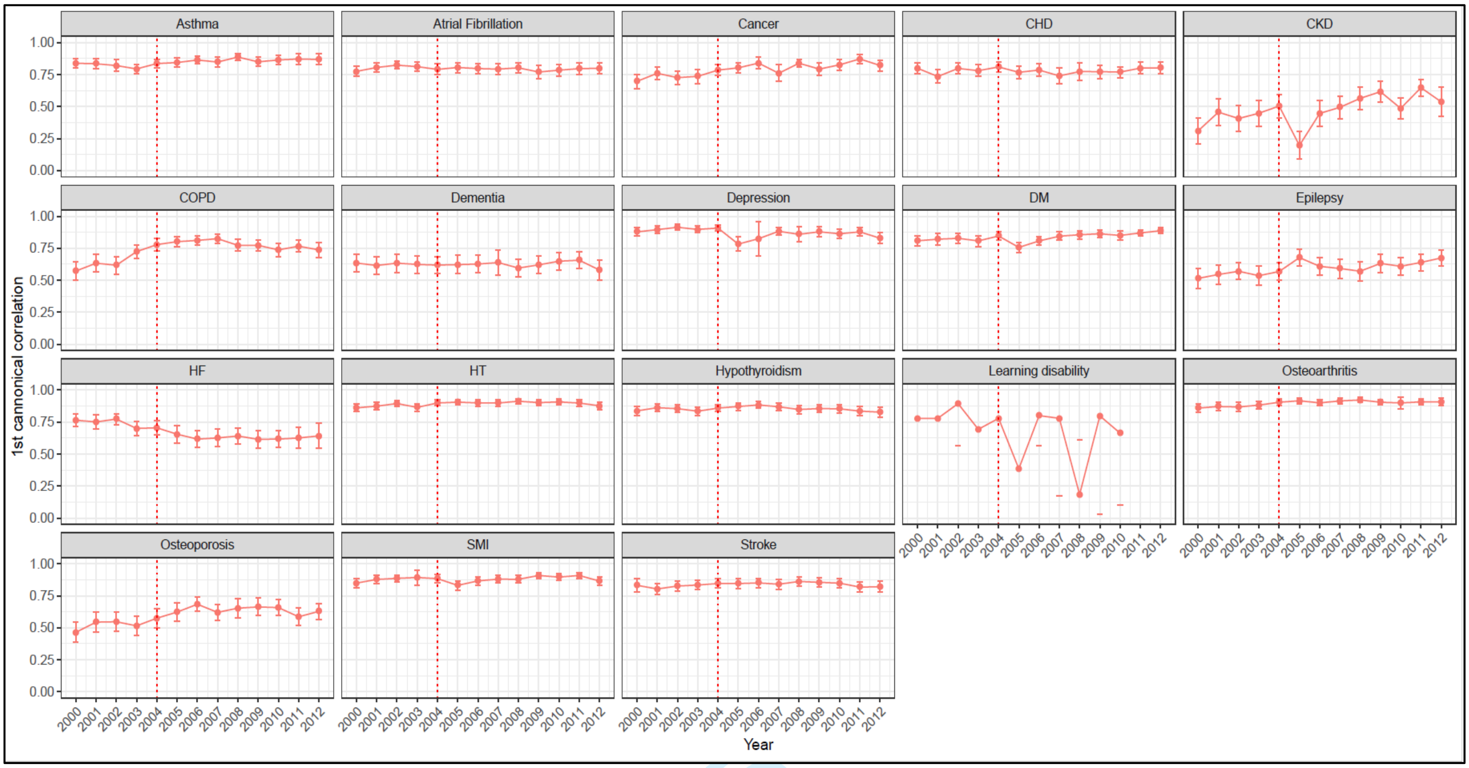

CHD, coronary heart disease; CKD, chronic kidney disease; COPD, chronic obstructive pulmonary disease; DM, diabetes mellitus; HF, heart failure; HT, hypertension; SMI, severe mental illness.

Incident code: a clinical code indicating new (incident) cases. The red line represents the launching year of the QOF in 2004.

For peer review only - http://bmjopen.bmj.com/site/about/guidelines.xhtml

Supplementary data to "Clinical code usage in UK general practice: a cohort study exploring 18 conditions over 14 years", Zghebi et al. 2021.

Figure S4 Bias-corrected canonical correlations using 5-year window for incident clinical code usage for 18 mental and physical conditions

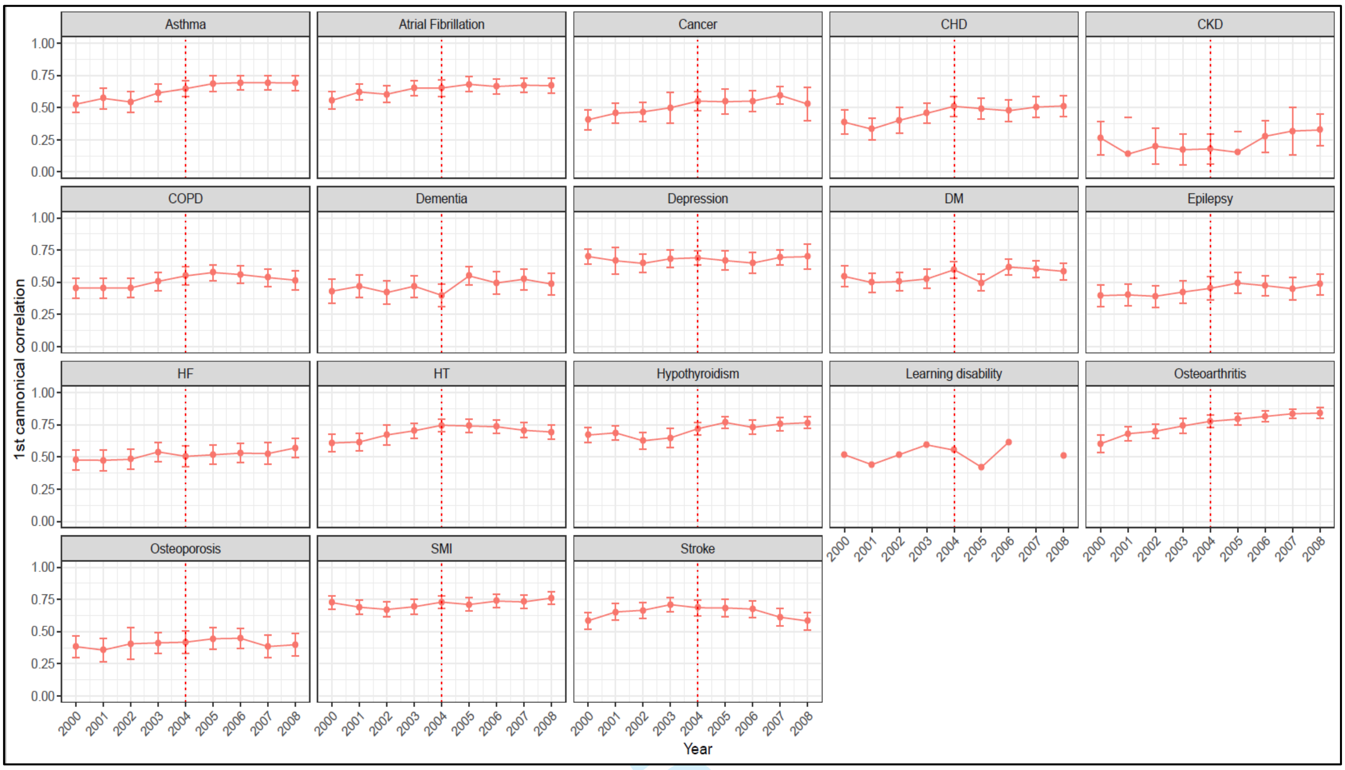

CHD, coronary heart disease; CKD, chronic kidney disease; COPD, chronic obstructive pulmonary disease; DM, diabetes mellitus; HF, heart failure; HT, hypertension; SMI, severe mental illness.

Incident code: a clinical code indicating new (incident) cases. The red line represents the launching year of the QOF in 2004.

For peer review only - http://bmjopen.bmj.com/site/about/guidelines.xhtml

The RECORD statement – checklist of items, extended from the STROBE statement, that should be reported in observational studies using routinely collected health data.

	Item No.	STROBE items	Location in manuscript where items are reported	RECORD items	Location in manuscript where items are reported
**Title and abstract**					
	1	(a) Indicate the study's design with a commonly used term in the title or the abstract (b) Provide in the abstract an informative and balanced summary of what was done and what was found	**Title and Abstract**	RECORD 1.1: The type of data used should be specified in the title or abstract. When possible, the name of the databases used should be included.<br><br>RECORD 1.2: If applicable, the geographic region and timeframe within which the study took place should be reported in the title or abstract.<br><br>RECORD 1.3: If linkage between databases was conducted for the study, this should be clearly stated in the title or abstract.	**Abstract**<br><br>**Title and Abstract**<br><br>**Not applicable**
**Introduction**					
Background rationale	2	Explain the scientific background and rationale for the investigation being reported	**Introduction**		
Objectives	3	State specific objectives, including any prespecified hypotheses	**Abstract, Introduction**		
**Methods**					
Study Design	4	Present key elements of study design early in the paper	**Methods**		
Setting	5	Describe the setting, locations, and relevant dates, including periods of recruitment, exposure, follow-up, and data collection	**Abstract, Methods**		
Participants	6	*(a)* ***Cohort study*** - Give the eligibility criteria, and the sources and methods of selection of participants. Describe methods of follow-up	**Methods**	RECORD 6.1: The methods of study population selection (such as codes or algorithms used to identify subjects) should be listed in detail. If this is not possible, an explanation should be provided.<br><br>RECORD 6.2: Any validation studies of the codes or algorithms used to select the population should be referenced. If validation was conducted for this study and not	**Methods**<br><br>**Not applicable**

For peer review only

		(b) ***Cohort study*** - For matched studies, give matching criteria and number of exposed and unexposed<br>*Case-control study* - For matched studies, give matching criteria and the number of controls per case	**Not applicable**	published elsewhere, detailed methods and results should be provided.<br><br>RECORD 6.3: If the study involved linkage of databases, consider use of a flow diagram or other graphical display to demonstrate the data linkage process, including the number of individuals with linked data at each stage.	**Not applicable**
Variables	7	Clearly define all outcomes, exposures, predictors, potential confounders, and effect modifiers. Give diagnostic criteria, if applicable.	**Methods**	RECORD 7.1: A complete list of codes and algorithms used to classify exposures, outcomes, confounders, and effect modifiers should be provided. If these cannot be reported, an explanation should be provided.	**Methods** (codes available from online repository: clinicalcodes.org)
Data sources/ measurement	8	For each variable of interest, give sources of data and details of methods of assessment (measurement). Describe comparability of assessment methods if there is more than one group	**Methods**		
Bias	9	Describe any efforts to address potential sources of bias	**Methods**<br>Also, the used population (CPRD) is representative of the UK population		
Study size	10	Explain how the study size was arrived at	**Methods**		
Quantitative variables	11	Explain how quantitative variables were handled in the analyses. If applicable, describe which groupings were chosen, and why	**Methods**		
Statistical methods	12	(a) Describe all statistical methods, including those used to control for confounding<br>(b) Describe any methods used to examine subgroups and interactions<br>(c) Explain how missing data were addressed<br>(d) ***Cohort study*** - If applicable, explain how loss to follow-up was addressed<br>(e) Describe any sensitivity analyses	**Methods**		
Data access and cleaning methods		**Not applicable in STROBE**		RECORD 12.1: Authors should describe the extent to which the investigators had access to	**Methods**

For peer review only

				the database population used to create the study population.<br><br>RECORD 12.2: Authors should provide information on the data cleaning methods used in the study.	 <br>**Not applicable**
Linkage		**Not applicable in STROBE**		RECORD 12.3: State whether the study included person-level, institutional-level, or other data linkage across two or more databases. The methods of linkage and methods of linkage quality evaluation should be provided.	**Not applicable**
**Results**					
Participants	13	(a) Report the numbers of individuals at each stage of the study (*e.g.*, numbers potentially eligible, examined for eligibility, confirmed eligible, included in the study, completing follow-up, and analysed)<br>(b) Give reasons for non-participation at each stage.<br>(c) Consider use of a flow diagram	**Not applicable**	RECORD 13.1: Describe in detail the selection of the persons included in the study (*i.e.,* study population selection) including filtering based on data quality, data availability and linkage. The selection of included persons can be described in the text and/or by means of the study flow diagram.	**Methods / Results**
Descriptive data	14	(a) Give characteristics of study participants (*e.g.*, demographic, clinical, social) and information on exposures and potential confounders<br>(b) Indicate the number of participants with missing data for each variable of interest<br>(c) ***Cohort study*** - summarise follow-up time (*e.g.*, average and total amount)	**Not applicable**		
Outcome data	15	***Cohort study*** - Report numbers of outcome events or summary measures over time	**Results**		
Main results	16	(a) Give unadjusted estimates and, if applicable, confounder-adjusted estimates and their precision (e.g., 95% confidence interval). Make clear which confounders were adjusted for and why they were included<br>(b) Report category boundaries when continuous variables were categorized	**Results, Figures 1-5, Figures S1 – S4.**		

For peer review only

		(c) If relevant, consider translating estimates of relative risk into absolute risk for a meaningful time period			
Other analyses	17	Report other analyses done—e.g., analyses of subgroups and interactions, and sensitivity analyses	**Not applicable**		
**Discussion**					
Key results	18	Summarise key results with reference to study objectives	**Discussion**		
Limitations	19	Discuss limitations of the study, taking into account sources of potential bias or imprecision. Discuss both direction and magnitude of any potential bias	**Discussion**	RECORD 19.1: Discuss the implications of using data that were not created or collected to answer the specific research question(s). Include discussion of misclassification bias, unmeasured confounding, missing data, and changing eligibility over time, as they pertain to the study being reported.	**Discussion / Conclusion**
Interpretation	20	Give a cautious overall interpretation of results considering objectives, limitations, multiplicity of analyses, results from similar studies, and other relevant evidence	**Discussion**		
Generalisability	21	Discuss the generalisability (external validity) of the study results	**Not applicable**		
**Other Information**					
Funding	22	Give the source of funding and the role of the funders for the present study and, if applicable, for the original study on which the present article is based	**Funding**		
Accessibility of protocol, raw data, and programming code		..		RECORD 22.1: Authors should provide information on how to access any supplemental information such as the study protocol, raw data, or programming code.	**Methods** (raw data can only by accessed via the CPRD)

*Reference: Benchimol EI, Smeeth L, Guttmann A, Harron K, Moher D, Petersen I, Sørensen HT, von Elm E, Langan SM, the RECORD Working Committee. The REporting of studies Conducted using Observational Routinely-collected health Data (RECORD) Statement. *PLoS Medicine* 2015; in press.

*Checklist is protected under Creative Commons Attribution (CC BY) license.

For peer review only
